# Response of Coastal *Shewanella* and *Duganella* Bacteria to Planktonic and Terrestrial Food Substrates

**DOI:** 10.3389/fmicb.2021.726844

**Published:** 2022-02-16

**Authors:** Li Zhao, Sonia Brugel, Kesava Priyan Ramasamy, Agneta Andersson

**Affiliations:** ^1^Department of Ecology and Environmental Science, Umeå University, Umeå, Sweden; ^2^Umeå Marine Sciences Centre, Umeå University, Hörnefors, Sweden

**Keywords:** coastal bacteria, *Duganella* sp., *Shewanella baltica*, river organic carbon, plankton organic carbon, bioavailability, bacterial growth efficiency, response

## Abstract

Global warming scenarios indicate that in subarctic regions, the precipitation will increase in the future. Coastal bacteria will thus receive increasing organic carbon sources from land runoff. How such changes will affect the function and taxonomic composition of coastal bacteria is poorly known. We performed a 10-day experiment with two isolated bacteria: *Shewanella baltica* from a seaside location and *Duganella* sp. from a river mouth, and provided them with a plankton and a river extract as food substrate. The bacterial growth and carbon consumption were monitored over the experimental period. *Shewanella* and *Duganella* consumed 40% and 30% of the plankton extract, respectively, while the consumption of the river extract was low for both bacteria, ∼1%. *Shewanella* showed the highest bacterial growth efficiency (BGE) (12%) when grown on plankton extract, while when grown on river extract, the BGE was only 1%. *Duganella* showed low BGE when grown on plankton extract (< 1%) and slightly higher BGE when grown on river extract (2%). The cell growth yield of *Duganella* was higher than that of *Shewanella* when grown on river extract. These results indicate that *Duganella* is more adapted to terrestrial organic substrates with low nutritional availability, while *Shewanella* is adapted to eutrophied conditions. The different growth performance of the bacteria could be traced to genomic variations. A closely related genome of *Shewanella* was shown to harbor genes for the sequestration of autochthonously produced carbon substrates, while *Duganella* contained genes for the degradation of relatively refractive terrestrial organic matter. The results may reflect the influence of environmental drivers on bacterial community composition in natural aquatic environments. Elevated inflows of terrestrial organic matter to coastal areas in subarctic regions would lead to increased occurrence of bacteria adapted to the degradation of complex terrestrial compounds with a low bioavailability.

## Introduction

Heterotrophic bacteria are important players in the biogeochemical cycles of the major elements on Earth. They have a dual role in the ecosystems as being the major mineralizers of organic matter into inorganic elemental forms and the contributors to the food web production (del Giorgio and Cole, 1998). Understanding how bacterial growth is regulated is of crucial importance, for example, to be able to construct models of the cycling of carbon, nitrogen, and phosphorus. In aquatic systems, the bacterial carbon biomass production, in general, follows the net primary production (e.g., del Giorgio and Cole, 1998), suggesting that organic matter produced by phytoplankton is the major substrate for heterotrophic bacteria. However, this view has been questioned, since bacterial carbon demand often exceeds phytoplankton primary production especially in low productive systems (Fouilland and Mostajir, 2010). In northerly estuaries profoundly influenced by river inflow from boreal regions, the bacterial production can even exceed the primary production (Figueroa et al., 2016; Andersson et al., 2018b). Thus, both autochthonous and allochthonous dissolved organic matter can constitute food sources for aquatic bacteria.

Dissolved organic carbon (DOC) is a major component of the dissolved organic matter in aquatic systems. The easily available fraction of the DOC is, however, quite low, 1–20% (Zweifel et al., 1993; Søndergaard and Middelboe, 1995; Pedler et al., 2014; Figueroa et al., 2016), due to the refractive character of old DOC (Bianchi, 2011). In contrast, the bioavailability of newly produced autochthonous organic carbon is generally considered to be high, and bacteria are quickly consuming this fraction. However, in some cases, the reactivity of autochthonous organic carbon sources has been shown to be relatively low (Catalán et al., 2013). Furthermore, it is likely that the availability of nitrogen and phosphorus also affects the assimilation of DOC by bacteria due to their stoichiometric demands.

A key variable for understanding ecosystem effects of bacterial activity is the bacterial growth efficiency (BGE), which indicates how much of the consumed organic carbon is channeled into new biomass and the proportion that is being remineralized. Earlier studies have shown that the BGE correlates positively with the bacterial growth rates and the net primary productivity in the ecosystem (del Giorgio and Cole, 1998; Alonso-Sáez et al., 2008). When the bacterial production decreases, a larger proportion of the consumed carbon is allocated for maintenance energy and the growth efficiency decreases (del Giorgio and Cole, 1998; Eiler et al., 2003; Vikström and Wikner, 2019). Thus, a larger fraction of the consumed carbon would be respired, and a smaller fraction used for building new biomass. BGE can, however, be affected by various factors, such as nutrients, temperature, substrate quality, and the adaptation of the bacterial community to the environment (Vázquez-Domínguez et al., 2007; Lønborg et al., 2011; González-Benítez et al., 2019; Baña et al., 2020; Yeh et al., 2020).

Field studies have shown that specific bacterial taxa dominate in different aquatic habitats (e.g., Teira et al., 2009; Herlemann et al., 2011; Broman et al., 2019). Betaproteobacteria and Actinobacteria are common in low salinity waters, while Gammaproteobacteria and Alphaproteobacteria are more abundant in high saline waters (e.g., Herlemann et al., 2011; Broman et al., 2019). This distribution may be explained not only by salinity variation but also by co-varying factors, like terrestrial organic carbon. Earlier studies have shown a link between high concentrations of humic substances in the environment and dominance of Betaproteobacteria (Teira et al., 2009), which might be explained by the presence of genes for the degradation of aromatic compounds in this bacterial group (Pérez-Pantoja et al., 2012). It is, however, so far unknown whether the Betaproteobacteria are utilizing terrestrial DOC more efficiently than other types of bacteria, and if that is coupled to specific genomic properties. Likewise, we lack information about the genetic and functional adaptation of bacteria that are known to thrive in eutrophied conditions, like Gammaproteobacteria (Andersson et al., 2018a). One way to approach that knowledge gap is to use single bacterial species isolated from different habitats, test these for growth on habitat-related substrates, and link to their genomic properties. If the bacteria are carefully isolated using relevant substrates, they may serve as models to study bacterial ecophysiology and biogeochemistry (Hagström et al., 2017).

Climate change is projected to cause ∼30% increased precipitation in the European subarctic region within the next 100 years, which will cause increased inflows of brown-colored humic-rich river water to the northern Baltic Sea (Andersson et al., 2018b). The coastal bacteria may thus experience increased availability of allochthonous terrestrial organic matter as food source, while the autochthonous substrate decreases as primary production diminishes due to light shading (Andersson et al., 2015, 2018b). Here we aimed to elucidate how such a change in food source would affect different coastal bacteria. An experiment was performed where two bacteria isolated from the northern Baltic Sea, *Duganella* sp. (Betaproteobacteria) and *Shewanella baltica* (Gammaproteobacteria), were provided two different types of substrates. One substrate consisted of ultrafiltered river water; the other was a marine plankton lysate. We hypothesized that the plankton extract would be more bioavailable than the river extract, and that the bacterial abundance yield should follow the bioavailability of the added food substrate. The BGE was expected to increase with the amount of added food substrate, but we assumed that quality should be even more important, i.e., that the high-quality plankton extract should result in high BGE and that low qualitative river extract should result in low BGE. Furthermore, we expected *Shewanella*, isolated from a seaside coastal site, to be more adapted to the utilization of the plankton extract and *Duganella*, isolated from a river mouth, to be more adapted to the utilization of the river extract. Finally, we anticipated that differences could be linked to genomic differences between the bacteria. This would imply that studies of isolated species can be ecologically relevant and assist in the understanding of ecosystem processes.

## Materials and Methods

### Media for Isolation of Bacteria

Bacteria were isolated on agar plates using two different media: a plankton extract and a river extract-based medium. Sterile aged seawater was used as the base of both media. Seawater was collected at 1.5-m depth at a coastal site in the northern Baltic Sea. The water was 0.22-μm-filtered and autoclaved, stored in the dark at 4°C for four weeks before use. Agar plates with sterile-filtered plankton and river extract were mixed with sterile aged seawater and 1.5% agar. The organic carbon concentration in both types of agar plates was 350 μmolC.l^–1^, which is similar to the DOC concentration in the Baltic Sea (e.g., Andersson et al., 2018b).

#### Plankton and River Extracts

To obtain the plankton extract, about 750,000 l of coastal water was pumped from 5-m depth during the spring period, and the plankton was collected on a 90-μm nylon mesh. The collected plankton was kept cold and dark until extraction. The plankton was extracted by adding glass beads to Eppendorf tubes and disintegrating the organisms using a tissue lyser (Tissue Lyser II, Qiagen). The extract was centrifuged for 10 min to remove cell debris, and the supernatant was collected, 0.2-μm-filtered, and stored at –20°C until analysis.

To analyze the plankton composition of the sample used for disintegration, a subsample was preserved with 2% Lugol’s solution and counted using the inverted microscope method (Utermöhl, 1958). Nanoplankton, microplankton, and mesozooplankton were counted at 400x, 100x, and 40x, respectively. Nano- and microplankton biomass was calculated from the geometric shape of the cells following the work of Olenina et al. (2006), and the cell carbon content was calculated according to Menden-Deuer and Lessard (2000). Zooplankton carbon biomass was calculated according to Hernroth (1985). The plankton extract was composed of high molecular weight biopolymers (data not shown).

To obtain a river extract, about 15 l of river water was collected 5 km upstream the Öre River mouth during spring. The river water was processed by tangential flow filtration using a Centramate cassette holder (PALL) fitted with a suspended-screen ultrafiltration cassette with an Omega membrane of 300 kDa (PALL) over 1 day to remove larger particles. The remaining filtrate (14 l) was then ultrafiltered using a cassette of 1 kDa (a suspended-screen ultrafiltration cassette with an Omega membrane, PALL) to a volume of 2.5 l. The river extract consequently consisted of high molecular weight organic compounds (>1kDa).

Both the plankton and river extracts were thus composed of high molecular weight organic compounds, representing dissolved organic matter (DOM) in the size range 1–200–300 nm (Benner and Amon, 2015).

#### Chemical Analyses

The carbon, nitrogen, and phosphorus concentrations in the plankton and river extracts were analyzed by measuring dissolved organic carbon (DOC), total N, and total P. DOC analyses were carried out on 0.2-μm-filtered (Supor Membrane Syringe Filter, non-pyrogenic, Acrodisc^®^) and acidified (8 mM HCl final concentration) extract samples on a Shimadzu TOC-5000 analyzer. Total phosphorus (TP) and total nitrogen (TN) were measured in unfiltered samples using a Seal QuAAtro39 autoanalyzer after an oxidation step using peroxodisulfate, according to standard analytical methods (Grasshoff et al., 1983).

#### Isolation of Bacteria

Heterotrophic bacteria were isolated at two sampling sites in the northern Baltic Sea in July 2017. One site was located in the Öre River mouth (63°30.8094, 19°43.80) and the other at the coastal seaside (63°30.3713, 19°49.14) (Supplementary Figure 1). Water was collected at 0.5-m depth using a Ruttner sampler. At the Öre River mouth, the salinity was 0.9 psu, the temperature was 14°C, and the DOC concentration was ∼667 μmolC.l^–1^. At the coastal site, the salinity was 4.4 psu, the temperature was 12.1°C, and the DOC concentration was ∼333 μmolC.l^–1^.

Triplicate 100-μl samples from the Öre River mouth and the coastal site were spread on river agar plates and plankton agar plates. The plates were incubated at 20°C in dark conditions for ∼10 days. Five colonies from each plate were re-streaked three times on the same type of agar plates to secure that single cell colonies were obtained. However, no bacteria was successfully isolated when samples from the coastal seaside were spread on river agar plates. Strains were then conserved in frozen stocks with 10% glycerol at −80°C until further processing. For the subsequent test, the strains were grown in liquid broth (peptone 10 g.l^–1^, yeast extract 5 g.l^–1^, aged seawater) at 20°C with shaking (50 rpm).

#### DNA Extraction and 16S rRNA Gene Sequencing

Ten bacteria isolated from the river mouth and the seaside location were randomly selected and analyzed. The strains were identified by 16S rRNA gene sequencing. DNA was extracted with a Powersoil DNA isolation kit (Qiagen) according to the manufacturer’s recommendations. The 16S rRNA gene was amplified with the general bacterial primers 27f (5′AGAGTTTGATCCTGGCTCAG-3′) and 1492r (5′-GGTTACCTTGTTACGACTT-3′) (Lane, 1991). The PCR mixture contained 0.2 μM of each primer and 40 ng of DNA in a total volume of 100 μl (HotStar Taq Master Mix Kit, Qiagen). The PCR cycling regime was (1) one cycle of 15-min denaturation and Taq activation at 95°C; (2) 30 cycles of 60 s at 95°C, 45 s at 50°C, and 90 s at 72°C; and (3) one final extension cycle of 10 min at 72°C. PCR products were verified by standard agarose gel electrophoresis, and about 300 ng of each amplified DNA was sent to Macrogen (Netherland) for paired-end sequencing. Nearly full-length 16S rDNA sequences were obtained. The sequences were deposited in GenBank under accession numbers MW032666 to MW032685.

The BLASTn program from the National Center for Biotechnology Information (NCBI) web pages^1^ was used to identify the isolated 20 bacterial strains based on 16S rDNA sequences. We searched for taxa with high sequence similarity using the EMBL/GenBank/DDBJ databases. Among the isolates from the seaside location, 60% were identified as *Shewanella baltica*, and among the isolates from the river mouth, 80% were identified as *Duganella* sp. (Supplementary Table 1). One *Shewanella baltica* (MW032670) and one *Duganella* sp. (MW032681) with the highest similarity were selected for the microcosm experiment.

#### Bioinformatic Analysis of Bacterial Carbon Metabolism

We performed *in silico* analysis to identify the protein homologs involved in C, N, and P metabolism by comparing with the closest publicly available genome in NCBI. The isolated bacterial strains showed closest similarities to *Shewanella baltica* OS117 (99.85%) and *Duganella* sp. AF9R3 (99.71%). The NCBI accession numbers are NC_017579.1 and NZ_CP051684.1. The completely annotated protein sequences are available following the below links.

https://www.ncbi.nlm.nih.gov/genome/browse/#!/proteins/434/166825%7CShewanella%20baltica%20OS117/

https://www.ncbi.nlm.nih.gov/genome/browse/#!/proteins/40889/888716%7CDuganella%20sp.%20AF9R3/

The NCBI accession number for each protein coding genes is included in Supplementary Table 2. Clusters of orthologous groups of proteins (COGs) functional categorization was performed using available tools on the JGI IMG database (Chen et al., 2021). The genomes of *Shewanella baltica* OS117 and *Duganella* sp. AF9R3 are available in the IMG database with the following genome IDs: 651053066 and 2884672076, respectively. The abundance profile tool was used to identify the presence/absence of specific genes for protein families in the genome.

### Microcosm Experiment

#### Experimental Design

One bacterium isolated from the coastal seaside location, *Shewanella baltica*, and one bacterium from the river mouth, *Duganella* sp., were used in a 10-day microcosm experiment, where their growth responses and functions were studied in relation to different food substrates.

Each bacterium was provided with four different food substrates: (1) plankton extract (final DOC concentration ∼667 μmolC.l^–1^), (2) river extract (final DOC concentration ∼583 μmolC.l^–1^), (3) river extract + phosphorus (∼583 μmolC.l^–1^), and (4) sterile seawater (Control, final DOC concentration ∼333 μmolC.l^–1^). The river extract addition followed a climate change scenario where the precipitation, and thus the river inflow, is estimated to increase 30–50% over the next coming 100 years (e.g., Eriksson-Hägg et al., 2014; Andersson et al., 2015). Furthermore, we added approximately a similar amount of carbon from the plankton extract as from the river extract in order to be able to test the effects of qualitative differences. To further test the effects of quality on the bacterial growth, we added phosphorus to one treatment with the river extract, since the river extract contained extremely little phosphorus. Sterile (autoclaved) seawater (0.2 μm filtered) served as the base medium in all treatments. The microcosms with solely sterile seawater served as a control. Each treatment had three replicates. The salinity in the experiment was 3.5–4 psu.

The microcosm experiment was performed in 2-l Erlenmeyer flasks with vented screw caps (3 replicates × 4 treatments × 2 bacteria = 24 microcosms). The water volume was 1 l, and the experiment was performed as a batch culture incubated at 15°C in the dark.

The bacteria were pre-grown in a liquid broth medium (aged seawater, tryptone 10 g.l^–1^, yeast extract 5 g.l^–1^), and the cell abundance was measured by flow cytometry. After washing the bacteria in sterile seawater, ∼10^5^ bacteria ml^–1^ were added to each microcosm. Samples were then taken from the microcosms on days 0, 3, 6, and 9. Bacterial abundance was monitored in order to measure bacterial growth yield. DOC was monitored in order to measure the bacterial consumption of DOC and the bioavailability of the different types of DOC. Bacterial growth yield and DOC consumption were used to estimate the bacterial growth efficiency. Nitrogen and phosphorus were measured to monitor bacteria-induced transformations.

#### Bacterial Abundance

Bacterial abundance was measured using flow cytometry. At each sampling day, 2 ml of sample was preserved with glutaraldehyde (0.1% final concentration) and stored at –80°C for subsequent analysis. Samples were stained with SYBR Green I (Invitrogen) to a final concentration of 1:10,000 (Marie et al., 2005). The samples were run at a flow rate of 60 μl.min^–1^ for 1 min using a BD FACSVerse™ flow cytometer (BD Biosciences) equipped with a 488-nm laser (20-mW output), with 1-μm microspheres (Fluoresbrite plain YG, Polysciences) as the internal standard. The average light side scatter was measured to follow the average cell volume of the bacterial population in each sample.

#### Bacterial Biomass

The bacteria were filtered onto 0.2-μm black polycarbonate filters, stained with SYBR Green I (Invitrogen) at a final concentration of 1: 10,000, and their size was measured under blue excitation with epifluorescence microscopy (magnification x 1000, Nikon Eclipse TiU). After calculating the cell volume, the biomass was calculated from Norland’s relationship: carbon biomass (mgC.cell^–1^) = 0.12 × 10^–9^ × CV^0.7^, where CV is the cell volume in μm^3^ (Norland, 1993).

#### Dissolved Organic Carbon, Total, and Inorganic Nitrogen and Phosphorus Tot N and Tot P

Dissolved organic carbon (DOC) was analyzed as described above. Inorganic phosphorus (phosphate, PO_4_^3–^) and dissolved inorganic nitrogen (DIN: nitrate + nitrite and ammonium) were measured using a Seal QuAAtro39 autoanalyzer according to standard analytical methods (Grasshoff et al., 1983). Total dissolved nitrogen (TDN) and phosphorus (TDP) were measured on 0.2-μm-filtered (Supor Membrane Syringe Filter, non-pyrogenic, Acrodisc^®^) samples as inorganic nutrients after an oxidation step using peroxodisulfate using a Seal QuAAtro39 autoanalyzer (Grasshoff et al., 1983).

#### Dissolved Organic Carbon Availability, Bacterial Cell Yield, and Growth Efficiency

The DOC bioavailability was calculated as the proportion of DOC that was consumed by bacteria at the end of the growth phase (day 6). Bacterial growth yield was calculated by subtracting the abundance/biomass at the end of the growth phase from the start abundance/biomass. The BGE was calculated by dividing the bacterial carbon biomass produced by the consumption of DOC for the corresponding period (del Giorgio and Cole, 1998).

### Statistical Analyses

The comparisons of treatments were analyzed using Kruskal–Wallis test followed by Mann–Whitney pair test, while the Epps–Singleton test with a small sample size correction was preferred for the comparison between bacterial taxa owing to the distribution of the data and the small sample size (Epps and Singleton, 1986). All statistical analyses were performed with the PAST 4.03 software (Hammer et al., 2001).

## Results

### Carbon Consumption and Bioavailability of the Plankton and River Extracts

**FIGURE 1:**
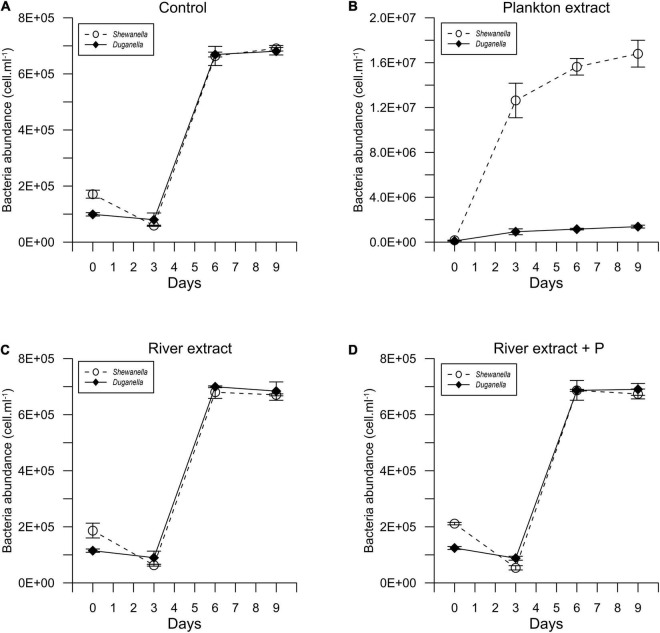
Temporal variation of the abundance of *Shewanella* and *Duganella* in the **(A)** control, **(B)** plankton extract, **(C)** river extract, and **(D)** river extract + P supplemented microcosms. Data points show average values, and error bars denote standard deviation.

**FIGURE 2:**
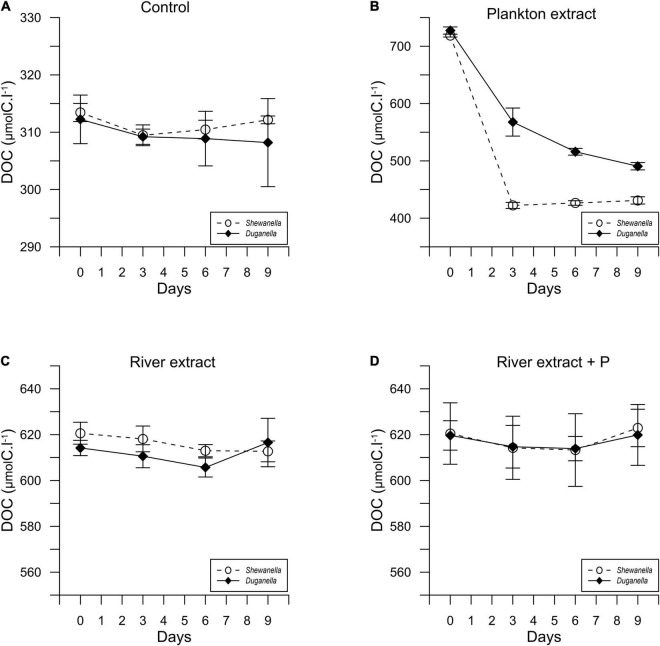
Temporal variation of the DOC concentration in *Shewanella* and *Duganella* incubations: **(A)** control, **(B)** plankton extract, **(C)** river extract, and **(D)** river extract + P. Data points show average values, and error bars denote standard deviation.

In most of the microcosms, the bacterial abundance increased from day 0 to day 6, whereby a plateau was reached (Figure 1). Concurrently, the DOC concentration decreased from day 0 to day 6 (Figure 2), as did also TDN and TDP concentrations in some of the incubations (Supplementary Figures 2, 3). The major reactivity time was thus identified to be from day 0 to day 6, and this period was used in further analysis of bacterial functions.

**FIGURE 3:**
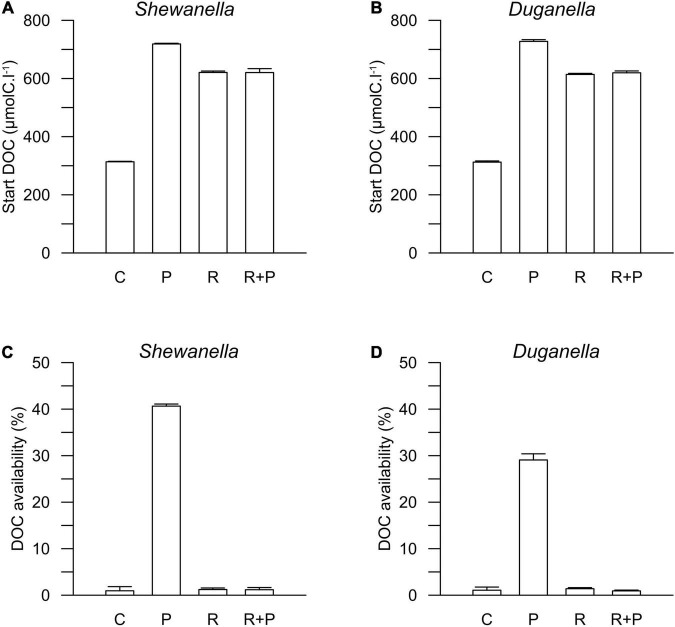
Start concentration of DOC in **(A)** *Shewanella* and **(B)** *Duganella* incubations, and DOC availability in **(C)** *Shewanella* and **(D)** *Duganella* incubations. C = control; P = plankton extract; R = river extract; R+P = river extract supplemented with phosphorus. Bar graphs show average values, and error bars denote standard deviation.

The DOC concentration at the start of the experiment was ∼316 μmolC.l^–1^ in the controls, 716 μmolC.l^–1^ in the plankton extract supplemented microcosms, and 616 μmolC.l^–1^ in the river extract supplemented microcosms (Figures 2, 3A,B). As described, our aim was to supply the two extract types in the same DOC concentration and according to a climate change scenario. Even though we did not supplement the extracts in the exact same concentrations, both were added within the range of the climate change scenario.

The plankton extract was mainly composed of lysed copepods (∼75%) and cladocerans (∼25%) (Supplementary Table 3). Additionally, the lysate also contained smaller shares of phytoplankton (diatoms, cyanobacteria, cryptophyceans, and chlorophyceans) and rotifers. The C:N molar ratio was 1,216, 825, and 825 in the plankton extract, the river extract, and the river extract + P, respectively (Table 1). Both the plankton and river extracts were composed of high molecular weight dissolved organic compounds. The plankton extract consisted of biopolymers, and the river extract consisted of terrestrial compounds. The C:P molar ratio was 78 and 58,947 in the plankton and river extracts, respectively (Table 1). To compensate for the extremely low P content in the river extract, PO_4_^3–^ was added to one set of the river extract supplemented microcosms. The start CNP stoichiometry in the different treatments showed similar trends as the extracts, but the values were adjusted as the extracts were diluted in sterile seawater, which constituted the experimental base medium (Table 1).

**TABLE 1:** C, N, and P stoichiometry (moles) of the plankton and river extracts, and start values in the Control, River, River + P, and Plankton incubations in the microcosm experiment.

Incubation	C:P	C:N	N:P
Plankton extract	71	1,216	0.1
River extract	58,947	825	71
Control	790	18	45
River	1,675	27	61
River + P	96	29	3
Plankton	78	5	16

The DOC availability, i.e., the DOC utilization, calculated from changes during the first 6 days of the experiment, ranged between 1 and 2% in the controls, the river extract, and the river extract + P supplemented microcosms, and the numbers were relatively similar for both the *Shewanella* and the *Duganella* bacterium (Figures 3C,D). The DOC bioavailability was the same in the control, based exclusively on seawater, than in the river extract treatments. Furthermore, the P addition to the river extract addition did not significantly increase the DOC availability. The bioavailability of the plankton extract treatment was significantly higher for *Shewanella* (40.6 ± 0.4%) compared to *Duganella* (29.1 ± 1.3%) (Epps-Singleton test, *p* < 0.01) (Figures 3C,D). *Shewanella* was thus more adapted to utilize the plankton extract than *Duganella*.

**FIGURE 4:**
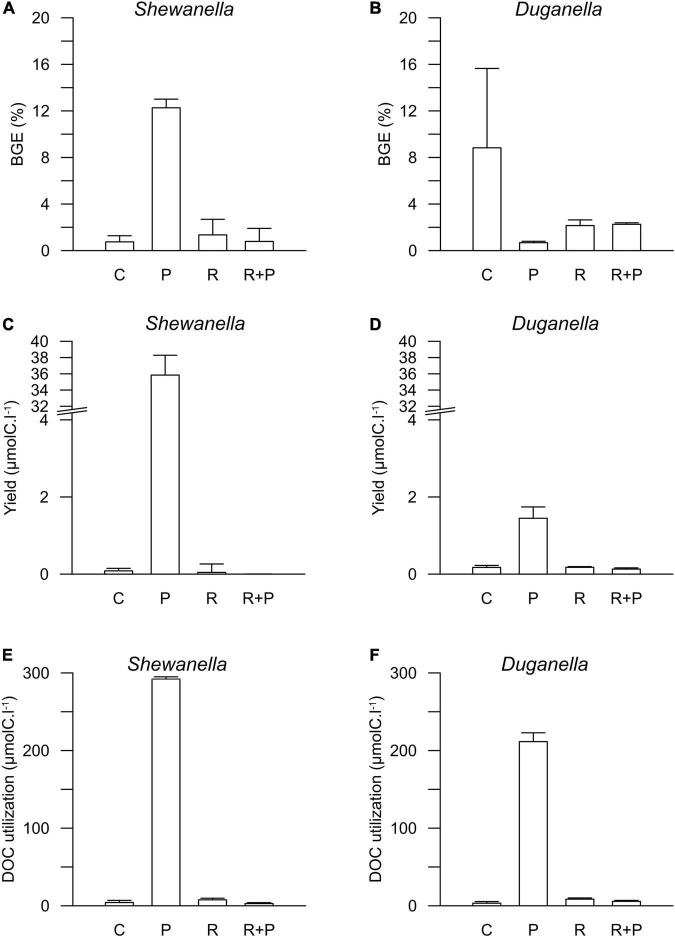
Bacterial growth efficiency (BGE) in **(A)** *Shewanella* and **(B)** *Duganella* incubations, bacterial biomass yield in **(C)** *Shewanella* and **(D)** *Duganella* incubations, and DOC utilization in **(E)** *Shewanella* and **(F)** *Duganella* incubations. C = control; P = plankton extract; R = river extract; R+P = river extract supplemented with phosphorus. Bar graphs show average values, and error bars denote standard deviation.

The DOC utilization was significantly higher in the plankton extract addition than in the river extract addition (Mann–Whitney test, *p* < 0.01; Figures 4E,F). The difference was large (30-fold) and could be explained not by the different amounts added to the microcosms but rather by quality differences.

### Variation in Nutrient Concentrations

The TDN concentration remained relatively constant in the control and river extract additions, while TDN was consumed in the plankton extract addition (Supplementary Figure 2), where *Shewanella* showed a higher consumption than *Duganella* (Epps–Singleton test, *p* < 0.01). This resulted in a high production of ammonium by both *Shewanella* and *Duganella* in the plankton extract addition (Supplementary Figure 3). The nitrate did not show large variations during the experiment in any of the treatments (Supplementary Figure 4), while the nitrite showed an increase in the plankton extract addition, which was slightly higher for *Duganella* (Supplementary Figure 5).

*Shewanella* was consuming TDP in all incubations, while for *Duganella*, a TDP consumption was observed only for the plankton extract amended microcosms (Supplementary Figure 6). In the plankton extract addition, the TDP consumption was higher for *Shewanella* than for *Duganella* (Epps–Singleton test, *p* < 0.01). In parallel, phosphate was taken up by *Shewanella* while it was produced by *Duganella* in the plankton extract addition (Supplementary Figure 7).

### Bacterial Abundance and Carbon Biomass and Cell Yield

At the start of the experiment, both bacteria were relatively large and had a high cell carbon content (Supplementary Table 4). However, over the time course of the experiment, when the cell abundances increased and the available carbon substrate was consumed, the bacteria decreased in size and lowered the cell carbon content. Such cell size decreases were observed for both bacteria and in all treatments.

The differences in DOC utilization mirrored the cell yield of bacteria (Figures 4C–F). The plankton extract triggered higher cell abundance and carbon biomass than the river extract and the control (Mann–Whitney test, *p* < 0.01; Figures 1, 4C,D and Supplementary Figure 8), especially for the *Shewanella* bacterium. At the start of the experiment, the bacterial abundance was ∼0.2 × 10^6^ cells.ml^–1^. In the control, the river extract addition, and the river extract + P addition, the end concentrations were ∼0.8 × 10^6^ cells.ml^–1^. However, in plankton extract supplemented microcosms, *Duganella* reached 1.5 × 10^6^ cells.ml^–1^, and *Shewanella* reached as much as 18 × 10^6^ cells.ml^–1^. The bacterial cell yield was relatively similar in the control, the river extract addition, and the river extract + P addition (Kruskal–Wallis, *p* < 0.01; Mann–Whitney, *p* > 0.1; Figures 4C,D). Nevertheless, the bacteria showed some differences when the river extract was the provided food source. The cell yields of *Duganella* were 5.8 × 10^5^ and 5.6 × 10^5^ cell.ml^–1^ in the river extract and the river extract + P, respectively, while the cell yields for *Shewanella* were 4.9 × 10^5^ and 4.8 × 10^5^ cell.ml^–1^ in the river extract and the river extract + P, respectively (Table 2). Taken together, the cell yield was significantly higher for *Duganella* in the river extracts compared to *Shewanella* (Epps–Singleton test, *p* < 0.01).

**TABLE 2:** Comparison of the DOC utilization, cell yield, carbon biomass yield, and growth efficiency (BGE) of the *Duganella* and the *Shewanella* bacterium in the Control, River extract, River extract + P and Plankton extract incubations in the microcosm experiment.

Treatment	Measure	*Duganella*	*Shewanella*
Control	DOC utilization (μmolC.l^–1^)	3.6 (± 2.1)	4.2 (± 2.7)
	Cell yield (cell.ml^–1^)	5.7 × 10^5^ (± 4 × 10^3^)	4.9 × 10^5^ (± 20 × 10^3^)
	Biomass yield (μmolC.l^–1^)	0.17 (± 0.05)	0.08 (± 0.07)
	BGE (%)	8.8 (± 6.8)	0.7 (± 0.5)
River extract	DOC utilization (μmolC.l^–1^)	8.5 (± 1.5)	7.6 (± 2.1)
	Cell yield (cell.ml^–1^)	5.8 × 10^5^ (± 9 × 10^3^)	4.9 × 10^5^ (± 48 × 10^3^)
	Biomass yield (μmolC.l^–1^)	0.18 (± 0.02)	0.05 (± 0.22)
	BGE (%)	2.2 (± 0.5)	1.4 (± 1.4)
River extract + P	DOC utilization (μmolC.l^–1^)	5.7 (± 1.1)	7.2 (± 2.8)
	Cell yield (cell.ml^–1^)	5.6 × 10^5^ (± 2 × 10^3^)	4.8 × 10^5^ (± 38 × 10^3^)
	Biomass yield (μmolC.l^–1^)	0.13 (± 0.03)	-0.11 (± 0.15)
	BGE (%)	2.3 (± 0.1)	0.8 (± 1.1)
Plankton extract	DOC utilization (μmolC.l^–1^)	211.5 (± 11.4)*	292.1 (± 2.9)*
	Cell yield (cell.ml^–1^)	11 × 10^5^ (± 76 × 10^3^)*	155 × 10^5^ (± 743 × 10^3^)*
	Biomass yield (μmolC.l^–1^)	1.45 (± 0.29)*	35.8 (± 2.4)*
	BGE (%)	0.7 (± 0.1)*	12.3 (± 0.7)*

Values are means ± standard deviation. *Denotes significant differences between taxa at p < 0.01 (Epps–Singleton test).

### Variation in Bacterial Growth Efficiency

The BGE values varied from < 1 to ∼12% in the different treatments (Figures 4A,B and Table 2). For *Shewanella*, the highest BGE (12%) was observed in the plankton extract addition (Figure 4A and Table 2), while the corresponding BGE for *Duganella* was only 0.7%. These BGE values were statistically different (Epps–Singleton test, *p* < 0.01; Table 2). *Duganella* showed slightly higher BGE values (1–8%) than *Shewanella* in the control, river, and river + P incubations, but these differences could not be statistically verified and therefore they should only be considered as indications. The addition of P to the river extract incubation did not lead to increased BGE of either of the two tested bacteria (Figures 4A,B and Table 2).

Furthermore, the results showed no indication that increased addition of carbon substrate, in general, caused elevated BGE. For *Duganella*, the highest BGE was observed in the control, where the DOC concentration was the lowest. Neither did *Shewanella* have consistently higher BGE in the river and river + P additions compared to the control.

### Bioinformatic Analysis of Carbon Metabolic Genes in the Closest Genome of *Shewanella baltica* sp. and *Duganella* sp.

*In silico* analysis of the closest genome of *Shewanella baltica* and *Duganella* sp. indicated that both studied bacteria have genes for the utilization of organic nitrogen compounds, nitrate assimilation, nutrient acquisition, and phosphate regulation (Table 3 and Supplementary Table 2). A total of 4,714 genes were assigned to functional COG categories in the *Duganella* genome, whereas in *Shewanella*, 3,115 genes were assigned to COG categories. Genes of functional category of carbohydrate transport and metabolism, inorganic ion transport and metabolism, signal transduction, and transcription are the most abundant group in the *Duganella* genome when compared to the *Shewanella* genome. The COG functional category and the distribution of the gene number in each category are listed in Supplementary Figure 9, and the complete comparative abundance list of predicted Pfam families is provided in Supplementary Table 5. The genome of *Shewanella baltica* harbors a gene involved in sucrose phosphorylase that is possibly involved in the regulation of sucrose metabolism, whereas this gene was absent from the genome of *Duganella* sp. In the genome of *Shewanella baltica*, we found a gene for glycogen phosphorylase, which is involved in the phosphorylase activity and degradation of glycogen. In addition, the *Shewanella baltica* genome includes a two-component system response regulator (ArcA) involved in the response to changes in oxygen levels. The genome of *Shewanella baltica* was found to have chitinase C involved in the metabolism for the utilization of chitin, whereas we did not identify genes encoding for chitinase in *Duganella* sp. The *Shewanella baltica* genome possesses Na^+^-translocating NADH:quinone oxidoreductase (Na^+^-NQR) encoding genes, which might function as the osmoregulation for sodium ion transport, whereas these genes are absent in the genome of *Duganella* sp. The gene encoding xylan esterase, cellulase, and pectin lyase was found in the genome of *Duganella* sp. for the enzymatic hydrolysis of lignocellulosic materials. The *Duganella* sp. genome harbors a gene encoding for gallate dioxygenase that degrades gallate. Moreover, a key gene for the utilization of aromatic carbon sources, like protocatechuate dioxygenase, is present in the genome of *Duganella* sp. but not in *Shewanella baltica*, and potentially reflects the adaptation to the river habitat of *Duganella* sp. Both these enzymes are needed in lignin decomposition, which is essential to the terrestrial carbon cycle.

**TABLE 3:** Comparative list of selected protein coding genes involved in carbon, nitrogen, and phosphorous metabolism in the closest genomes of *Shewanella* and *Duganella* species.

Protein coding genes	*Shewanella baltica* OS117	*Duganella* sp. AF9R3	Functional roles	References
Carbon–nitrogen hydrolase family protein	+	+	Reduction of organic nitrogen compounds	Bork and Koonin, 1994
Nitrate reductase/nitrate ABC transporter permease	+	+	Nitrate assimilation	Smith et al., 2007
Two-component sensor histidine kinase	+	+	Nutrient acquisition and phosphate regulation	Santos-Beneit, 2015; Held et al., 2019
Sucrose phosphorylase	+	–	Sucrose phosphorylase activity	Müller and Wegmann, 1978
Glycogen/starch/alpha-glucan phosphorylase	+	–	Glycogen phosphorylase activity, linear malto-oligosaccharide phosphorylase activity, alpha-glucan phosphorylase activity	Schinzel and Nidetzky, 1999
Chitinase C	+	–	Hydrolysis of chitin by the enzyme bound to chitin	Watanabe et al., 1994
Na^+^-translocating NADH-quinone reductase	+	–	Sodium ion transport	Hayashi et al., 2001
Two-component system response regulator ArcA	+	–	Response to changes in oxygen levels	Gao et al., 2008
Gallate dioxygenase	–	+	Ring-cleavage dioxygenase (gallate degradation)	Kasai et al., 2005; Nogales et al., 2011
Cytochrome P450	–	+	Polycyclic aromatic hydrocarbon degradation	Brezna et al., 2006
Protocatechuate 3,4-dioxygenase	–	+	Benzoate degradation via hydroxylation and 2,4-dichlorobenzoate degradation	Orville et al., 1998
Cellulase	–	+	Involved in degradation of cellulose	López-Mondéjar et al., 2016
Xylan esterase	–	+	Hydrolysis of lignocellulosic materials	López-Mondéjar et al., 2016
Pectin lyase	–	+	Pectin degradation	Hugouvieux-Cotte-Pattat et al., 2014

*+, –represent encoding genes present or absent in the closely related genome.*

## Discussion

### Plankton Extract More Bioavailable Than River Extract

The plankton extract was more bioavailable (30–40%) than the river extract (1–2%) for both bacteria. The varying bioavailability was not due to a major difference in the molecular size of the extracts, since both consisted of high molecular weight compounds. However, they originated from different environments and were composed of biopolymers and terrestrial organic compounds, respectively, with large differences in elemental stoichiometry.

*Shewanella* showed 1.5 times higher DOC consumption of the plankton extract than did *Duganella*, and the abundance and biomass carbon yield were ∼20-fold higher for *Shewanella*. The DOC availability of the river extract was similarly low for both of the bacteria. We expected that a shortage of phosphorus could be a limiting factor for DOC degradation, but the addition of P to the river extract did not increase the DOC bioavailability. Thus, the riverine DOC was truly refractory, and the low bioavailability was not due to a stoichiometric mismatch.

Many attempts have been made to identify coastal bacterial communities that are adapted to the degradation of terrestrial organic matter (e.g., Asmala et al., 2013; Herlemann et al., 2014; Figueroa et al., 2016). Most studies show that coastal bacteria are only able to degrade a small fraction of the coastal DOC and the riverine DOC entering the coast (e.g., Zweifel et al., 1993; Herlemann et al., 2014; Figueroa et al., 2016). Herlemann et al. (2014) performed an experimental study on the utilization of terrestrial DOM by a coastal bacterial community. They found that the DOC utilization was relatively low (4–16%), and that neither the DOM composition nor the bacterial community composition changed during the experiment. They concluded that terrestrial DOC cannot be efficiently utilized by coastal bacteria. Our results are in line with these findings, as only ∼2% of the DOC in the river extract was bioavailable for degradation by the bacteria. The lack of identified difference in DOC utilization between *Shewanella* and *Duganella* might be explained by a small consumption in a large pool. The variations were likely below the detection limit of the used analytical method.

We conclude that the plankton extract was much more bioavailable (20-fold) than the river extract, and that *Shewanella* was the most efficient user of the plankton extract. In contrast, the river extract was used to the same low degree by both bacteria. The river extract had similarly low bioavailability as the aged coastal seawater (control).

### *Shewanella* Adapted to Autochthonous Carbon—*Duganella* to Terrestrial Carbon

At large, the measured range (from < 0.7 to ∼12%) of the BGE of the bacteria is in agreement with earlier studies performed in natural aquatic systems, like coastal waters, freshwaters, and oceanic waters (e.g., del Giorgio and Cole, 1998; Lee et al., 2009). *Shewanella* showed the highest BGE when the plankton extract was provided as the food substrate, while the river extract did not cause any elevated BGE. The plankton extract was considered to be of good quality because of its relatively high bioavailability and balanced C:P stoichiometry (close to the Redfield ratio). We interpret the BGE result as quality being an important factor for the BGE of *Shewanella*, while quantity itself is not important, at least within the tested range of concentrations. Even though the plankton extract also showed higher bioavailability for *Duganella* than in the control and the river extract, the biomass yield was not as pronounced as for *Shewanella* and the BGE was at minimum. *Duganella* consumed a substantial amount of plankton DOC, which did not translate into biomass. The carbon consumed by *Duganella* was likely respired, as efficient metabolic pathways were lacking as compared to the *Shewanella* bacterium. Unfavorable salinity could also cause increased respiration. We conclude that for this bacterium, neither good substrate quality nor high substrate concentration induced a high BGE. Instead, the highest BGE was observed in the control, which had relatively low DOC concentration of low quality (aged seawater).

In *Shewanella*, the presence of chitinase gene may function in the decomposition of chitin, which is the exoskeleton of some planktonic crustaceans (Souza et al., 2011). Furthermore, *Shewanella* has genes coding for phosphorylase enzymes that catalyze the addition of phosphates to carbon sources such as sucrose and glycogen (Table 3). The TDP consumption was twice as high for *Shewanella* than for *Duganella*. In the *Shewanella* microcosms, phosphate was consumed, while it was produced where *Duganella* was the inoculated bacterium. Furthermore, the *Shewanella* and *Duganella* genomes contain nitrate and nitrite reductase genes. However, genes for efficient reduction to ammonia were only found in *Shewanella*’s genome (*nap B, nap C, nap D, nap E*, *nrfA*), which may render *Shewanella* more efficient at ammonium production when grown on the plankton extract (Supplementary Figure 3). Altogether, this shows that *Shewanella* is better adapted in utilizing the phosphorus-rich plankton extract containing easily available carbon sources than *Duganella*. Opportunistic bacteria, such as the studied *Shewanella*, would thus be functionally and genetically adapted to a life in eutrophied aquatic environments, where dead planktons are being degraded. In fact, the genus *Shewanella* has been shown to occur abundantly in such waters (Deng et al., 2019).

Even though the measured DOC consumption was low for both bacteria when the river extract was the provided food source, we found indications of differences in the performance of the bacteria, measured as BGE, cell yield, and carbon biomass yield. When the river extract was provided as the food substrate, *Duganella* showed slightly higher average BGE (1.6 times) and carbon biomass yield (4 times) than *Shewanella*, although with a large variation within treatment. The abundance yield of *Duganella* was significantly higher than of *Shewanella* when pooling the river extract and the river extract + P treatments. In the search of information in a closely related genome, we found that *Duganella* has the capability to produce several enzymes for the degradation of refractory material, such as lignocellulose and gallate, which *Shewanella* does not have (Table 3). Furthermore, genes encoding for cellulase, pectin lyase, and xylan esterase, present in *Duganella* but absent in *Shewanella*, may be involved in the hydrolysis of lignocellulosic material originating from terrestrial plants in the riverine extract. Altogether, the presence of genes in *Duganella* for aromatic compound degradation seems to have contributed to the adaptation of this bacterium in the riverine extract. In line with this, a recent study reported higher relative abundances of *Duganella* in forest riverine systems than in degraded rivers (Lin et al., 2019). In a famine environment, as in nutrient poor boreal rivers, carrying such genetic properties may mean survival and a competitive advantage in the environment.

We conclude that the utilization of the plankton extract as food source resulted in a relatively high carbon biomass production and BGE for *Shewanella* but not for *Duganella*. The reason for this difference is probably that *Shewanella* possesses genes for enzymes degrading autochthonously produced organic matter, while these are missing in *Duganella*. Another possibility is that the growth of *Duganella* was somewhat hampered due to the brackish water incubation. However, when the river extract was the provided food source, *Duganella* showed signs of being the more efficient bacterium, which may be explained by the presence of genes for the degradation of terrestrial organic matter.

### Link to the Occurrence of Bacteria in Rivers and Coasts

It may be questioned how relevant it is to perform experiments with isolated organisms, since only a tiny fraction of all bacteria in aquatic environments are cultivable (Steen et al., 2019). However, the isolated bacteria we used in this study were representatives of common groups of bacteria in rivers and coasts. A Betaproteobacterium (*Duganella*) was isolated in the river mouth, and a Gammaproteobacterium (*Shewanella*) was isolated from the seaside area. The isolation of the bacteria did not occur randomly. Plankton extract was used for isolating coastal bacteria, while river extract was used for isolating river bacteria. The genus *Shewanella* constituted 60% of the coastal isolates, while the genus *Duganella* constituted 80% of the river isolates (Supplementary Table 1). Whereas it was possible to obtain bacterial isolates from both the coast and the river on agar plates with the plankton extract, no or very few colonies from the coastal water were growing on agar plates with the river extract. The bacteria were thus isolated on substrates originating from their natural environments. This indicates that we isolated ecologically relevant species, and that the results of the experiments would mirror their functions in the ecosystems.

Betaprotebacteria have been shown to be relatively abundant in freshwater, while Gammaproteobacteria are more common in nutrient-rich, saline waters (Herlemann et al., 2011). The study by Herlemann et al. (2011) showed that the relative abundance of Gammaproteobacteria increases along the north–south salinity and productivity gradient of the Baltic Sea. The salinity ranges between 2 and 7 psu in the gradient, while the primary production and the phosphorus concentrations show a 10- to 20-fold increase from north to south in the Baltic Sea (e.g., Andersson et al., 2015). Since Gammaproteobacteria are known to thrive in nutrient-rich environments (Andersson et al., 2018a), this could be part of the explanation for their salinity distribution pattern. Even if salinity can be an explanatory factor itself, the results of the present study conform to the nutrient-scavenging ecotype, which is common in some Gammaproteobacteria (e.g., Andersson et al., 2018a).

Salinity is a strong governing factor for the bacterial community composition in freshwater lakes and oceans (Herlemann et al., 2011; Colatriano et al., 2018). Marine bacteria have evolved strategies to manage elevated salinity concentrations, including a Na^+^-dependent respiratory chain, to counteract the osmotic stress (Walsh et al., 2013). However, at both our sampling sites, the salinity was <5 psu, indicating that other environmental factors could have been of importance. Previous studies have shown that *Duganella* as well as *Shewanella* occur in both freshwater and brackish water (*Duganella*: Rappé et al., 2000; Lin et al., 2019) (*Shewanella*: Deng et al., 2014; Mathisen et al., 2016; Castillo et al., 2018). Both bacteria were isolated from brackish water, at a salinity of 0.9 and 4.4 psu, respectively, and the experiment was performed at a salinity of 3.5–4 psu. However, we found that *Shewanella* harbored a gene for Na^+^-dependent respiratory chain (NQR) while *Duganella* did not. Differential salinity adaptation could thus be one of the explanatory factors for the varying results of the two bacteria. The growth of *Duganella* might have been somewhat hampered in the incubation salinity, while it was not the case for *Shewanella*.

The DOC concentration at the sampling sites was approximately twice as high in the river mouth (∼667 μmolC.l^–1^) than in the coast (∼333 μmolC.l^–1^), and the composition was also likely different, with more terrestrial material in the river mouth and more organic compounds originating from the marine system at the coastal site (Deutsch et al., 2012). We found genetic differences between the bacteria for carbon utilization, where *Duganella* is more versatile in the carbon utilization and has specific enzymes for the acquisition of aromatic compounds, while *Shewanella* has enzymes for the acquisition of carbohydrates such as glycogen sucrose and chitin. Accordingly, we think that the observed experimental differences mirror the adaptation to the carbon substrate composition and availability in the habitats from which the bacteria were isolated. Thus, the isolated bacteria seem to be ecologically relevant organisms, showing different adaptation to riverine and autochthonous marine food sources.

## Conclusion and Outlook

The main findings of this study were that *Shewanella* was more adapted to utilize the plankton extract as the food source than *Duganella*, while *Duganella* showed signs of being better in utilizing the river extract as the food source. Furthermore, we can, at least partly, link these variations to differences in their genomes. A closely related genome of the studied *Shewanella* isolate was shown to harbor genes for the sequestration of autochthonously produced carbon substrates, such as sucrose and chitin. In contrast, a closely related genome of the studied *Duganella* lacked these properties. Instead, the genomic comparison indicated that the studied *Duganella* bacterium contained genes for the degradation of relatively refractive lignocellulosic material and gallate. However, these properties only caused a slightly average growth advantage on the river extract, which could not be clearly verified in this short-term experiment. Furthermore, differential salinity adaptation might have influenced the results.

In a long-term perspective, however, it might be beneficial for bacteria in coastal areas to carry genes encoding the degradation of terrestrial organic compounds, such as lignocellulosic material. In line with this, Chloroflexi bacteria in the Canada Basin, Western Arctic Ocean, have been shown to be replete with aromatic compound degradation genes, potentially enabling the bacteria to degrade organic matter originating from the terrestrial system (Colatriano et al., 2018). Lateral gene transfer from terrestrial to marine bacteria was suggested to be one of the causative factors. The ability of bacteria to grow, even though slowly, on such compounds would mean a selective advantage compared to non-adapted bacterial taxa. Modeling studies indicate that, on a yearly basis, significant degradation of terrestrial organic carbon does occur in the Baltic Sea (Wikner and Andersson, 2012; Fransner et al., 2019). Moreover, according to global change models, the precipitation will increase in subarctic areas during the next 100 years (Andersson et al., 2015), and thus the inflow of terrestrial organic matter will increase in the coastal areas. A future perspective would thus be that the composition of the heterotrophic bacterial community in subarctic coastal areas will be driven toward taxa having a metabolic machinery for the degradation of complex terrestrial organic compounds.

## Data Availability Statement

The datasets presented in this study can be found in online repositories. The names of the repository/repositories and accession number(s) can be found in the article/Supplementary Material.

## Author Contributions

LZ, SB, and AA designed, performed, and analyzed the experiment. LZ and KR performed the molecular analyses. LZ, SB, KR, and AA wrote the manuscript. All authors contributed to the article and approved the submitted version.

## Conflict of Interest

The authors declare that the research was conducted in the absence of any commercial or financial relationships that could be construed as a potential conflict of interest.

## Publisher’s Note

All claims expressed in this article are solely those of the authors and do not necessarily represent those of their affiliated organizations, or those of the publisher, the editors and the reviewers. Any product that may be evaluated in this article, or claim that may be made by its manufacturer, is not guaranteed or endorsed by the publisher.

## Footnotes

1
http://www.ncbi.nlm.nih.gov/BLAST/
