# Supplementary material for: Response of Coastal Shewanella and Duganella Bacteria to Planktonic and Terrestrial Food Substrates
Source: Front Microbiol. 2022 Feb 16;12:726844. doi: 10.3389/fmicb.2021.726844 (PMC8888917; doi:10.3389/fmicb.2021.726844)
Supplement: Supplementary file 1 [file Presentation_1.pdf]

Supplementary information

Li Zhao, Sonia Brugel, Kesava Priyan Ramasamy, Agneta Andersson. Response of coastal *Shewanella* and *Duganella* bacteria to planktonic and terrestrial food substrates

Supplementary Table 1. Identification of the 20 isolated bacterial strains. Habitat location, top hit similarities and access number in GenBank are presented.

Location	Species name	Closest hit	Similarity	Accession number
**Seaside**	*Shewanella baltica* PP003	*Shewanella baltica* OS117	99.85%	MW032670
**Seaside**	*Shewanella baltica* PP001	*Shewanella baltica* OS117	99.78%	MW032668
**Seaside**	*Shewanella baltica* PP006	*Shewanella baltica* OS117	99.64%	MW032672
**Seaside**	*Shewanella baltica* PP004	*Shewanella baltica* strain CD-1	100%%	MW032666
**Seaside**	*Shewanella baltica* PP005	*Shewanella baltica* strain SeaQual_254/10	99.85%	MW032671
**Seaside**	*Shewanella baltica* PP002	*Shewanella baltica* strain SeaQual_198N	99.50%	MW032669
**Seaside**	*Pseudomonas gessardii* PP007	*Pseudomonas gessardii* strain SeaQual_P_B791/	100%	MW032667
**Seaside**	*Pseudomonas gessardii* PP018	*Pseudomonas gessardii* strain SeaQual_P_B791/1	99.78%	MW032675
**Seaside**	*Pseudomonas gessardii* PP008	*Pseudomonas gessardii* strain SeaQual_P_B845N	99.64%	MW032673
**Seaside**	*Pseudomonas gessardii* PP017	*Pseudomonas gessardii* strain OBE3	99.64%	MW032674
**River mouth**	*Duganella* sp. RR008	*Duganella* sp. AF9R3	99.71%	MW032681
**River mouth**	*Duganella* sp. RR007	*Duganella* sp. AF9R3	99.64%	MW032680
**River mouth**	*Duganella* sp. RR001	*Duganella* sp. AF9R3	99.57%	MW032676
**River mouth**	*Duganella* sp. RR002	*Duganella levis* strain CY42W	99.57%	MW032677
**River mouth**	*Duganella* sp. RR009	*Duganella levis* strain CY42W	99.43%	MW032682
**River mouth**	*Duganella* sp. RR010	*Duganella levis* strain CY42W	99.29%	MW032683
**River mouth**	*Duganella* sp. RR003	*Duganella* sp. HMD2171	99.85%	MW032678
**River mouth**	*Duganella* sp. RR004	*Duganella* sp. HMD2171	99.78%	MW032679
**River mouth**	*Aeromonas* sp. RR011	*Aeromonas sp. 1805*	100%	MW032684
**River mouth**	*Aeromonas* sp. RR012	*Aeromonas* sp. 1805	100%	MW032685

Supplementary Table 2. Locus tag and NCBI accession number for genes encoding for protein homologs involved in C, N and P metabolism in the strains *Shewanella baltica* OS117 and *Duganella* sp..

**Protein coding genes**	***Shewanella baltica* OS117**	***Duganella* sp. AF9R3**
Carbon-nitrogen hydrolase family protein	SBAL117_RS20125 WP_011847929.1	HH213_RS13110 WP_169112523.1
Nitrate reductase/nitrate ABC transporter permease	SBAL117_RS10150 WP_011846662.1	HH213_RS08050 WP_169111907.1
Two component sensor histidine kinase	SBAL117_RS06730 WP_011846193.1	HH213_RS26750 WP_169114292.1
Sucrose phosphorylase	SBAL117_RS22630 WP_011848240.1	-
Glycogen/starch/alpha-glucan phosphorylase	SBAL117_RS07190 WP_011846264.1	-
Chitinase C	SBAL117_2627 AEH14333.1	-
Na^+-translocating NADH-quinone reductase	SBAL117_RS04465 WP_011845864.1	-
Two-component system response regulator ArcA	SBAL117_RS03575 WP_011845892.1	-
Gallate dioxygenase	-	HH213_RS29575 WP_169114804.1
Cytochrome P450	-	HH213_RS01020 WP_229263242.1
Protocatechuate 3,4-dioxygenase	-	HH213_RS15590 WP_169112813.1
Cellulase	-	HH213_RS01680 WP_229263254.1
Xylan esterase	-	HH213_RS01665 WP_169110348.1
Pectin lyase	-	HH213_RS16180 WP_229263026.1

Supplementary Table 3. Relative carbon biomass of plankton organisms in the plankton extract.

Plankton group	Relative carbon biomass (%)
Copepods	80.1
Cladocerans	19.1
Rotifers	0.7
Diatoms	0.02
Cyanobacteria	0.05
Chlorophyceans	0.0006
Cryptophyceans	0.00004

Supplementary Table 4. Bacterial size, cell volume and cell carbon content at day 0 and day 6 during the experiment. Mean values ± standard deviation are presented.

Day	Treatment	*Shewanella*			*Duganella*		
		Size (µm)	Cell volume (μm^3)	Carbon content ($fgC.cell^{-1}$)	Size (µm)	Cell volume (μm^3)	Carbon content ($fgC.cell^{-1}$)
0		0.7 x 2	0.680	92	0.4 x 2.5	0.297	51
6	Control		0.107±0.005	25.1±0.8		0.032±0.003	10.7±0.6
6	River		0.112±0.003	25.9±0.4		0.035±0.001	11.5±0.3
6	River + P		0.114±0.006	27.1±0.0		0.036±0.001	11.7±0.3
6	Plankton		0.128±0.006	28.5±0.9		0.075±0.009	19.5±1.6

Supplementary Figure 1.

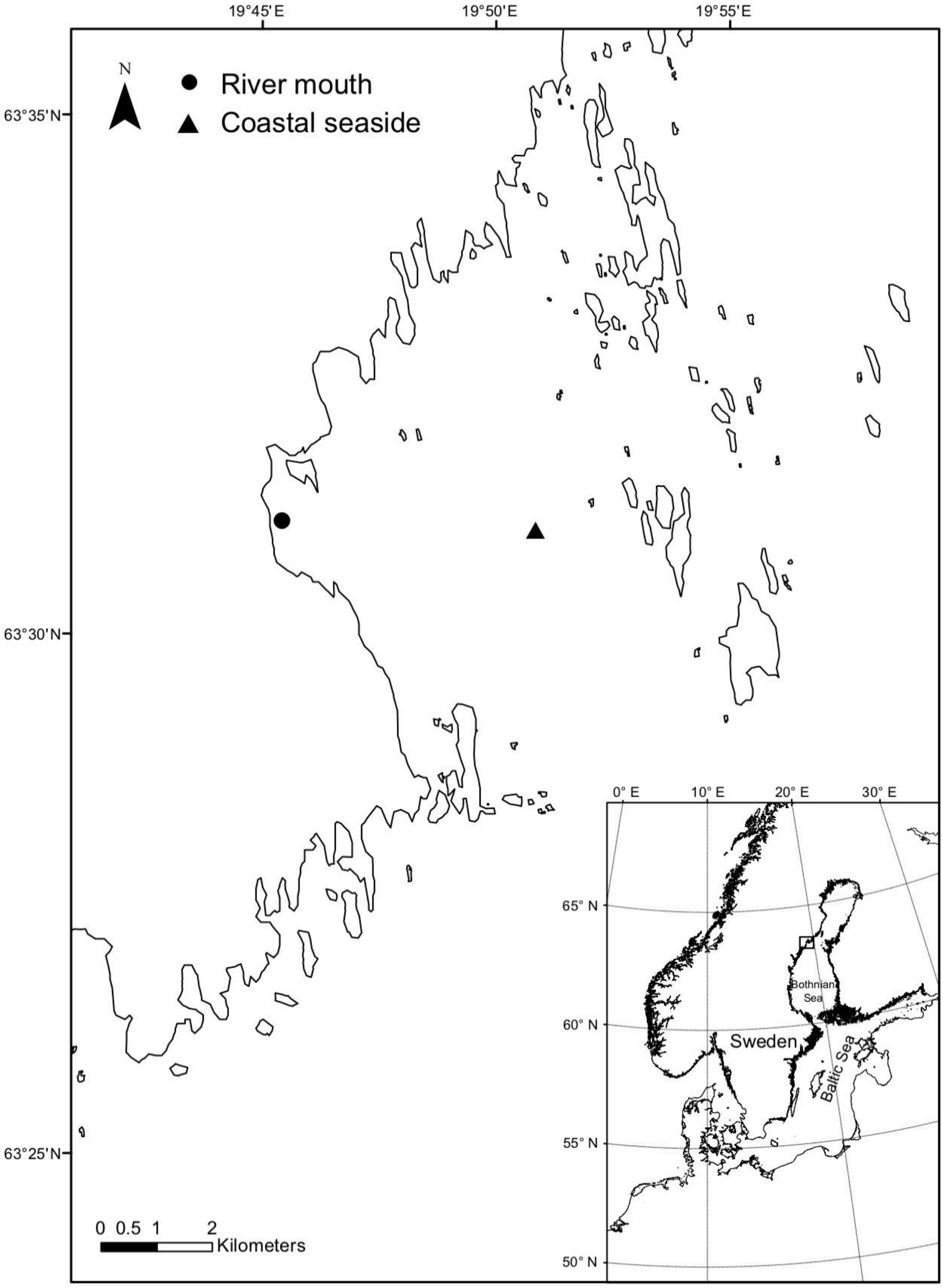

Supplementary Figure 1. Locations of the sampling sites in the northern Baltic Sea: coastal seaside and Öre River mouth.

Supplementary Figure 2.

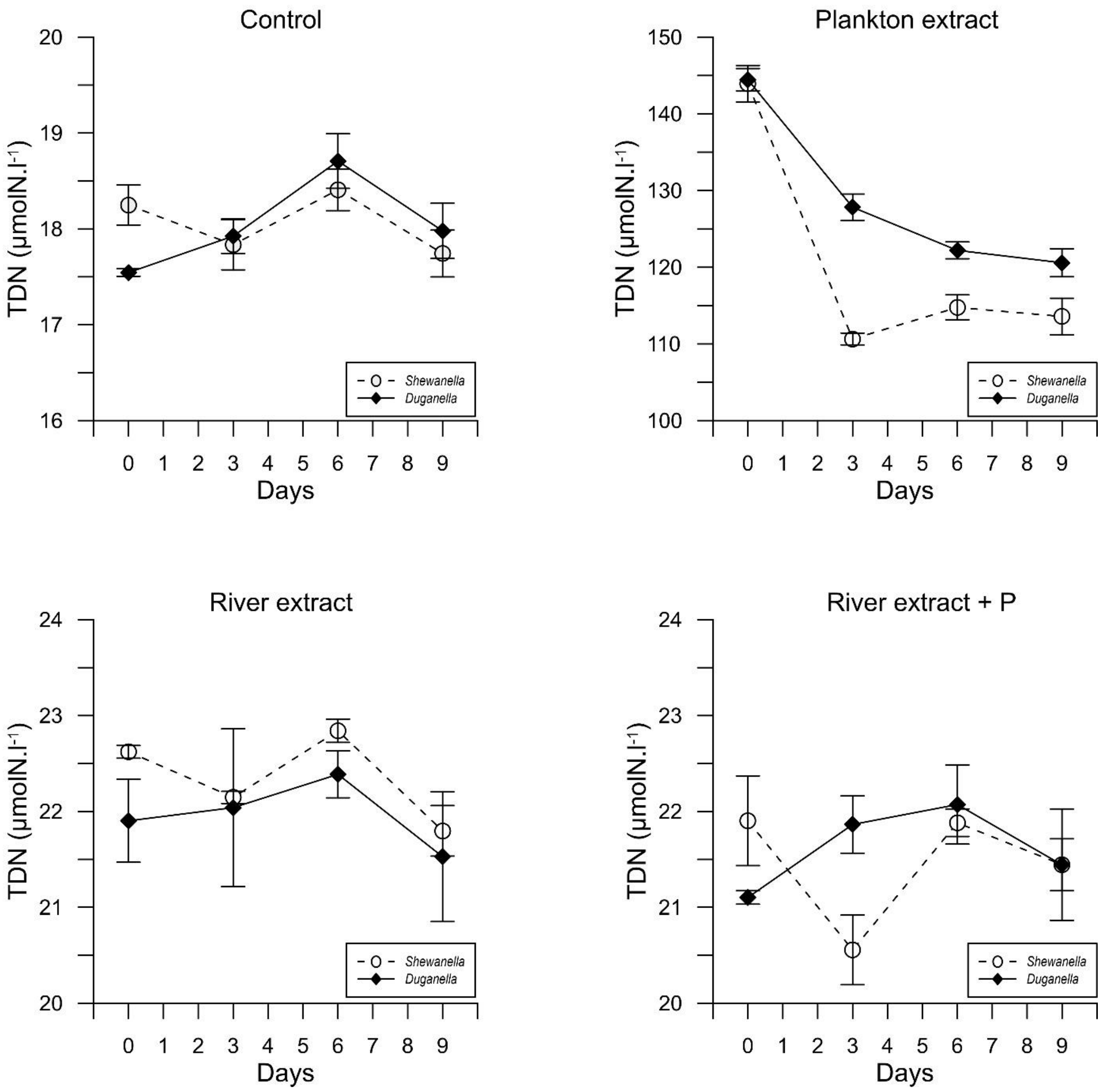

Supplementary Figure 2. Temporal variation of total dissolved nitrogen (TDN) concentration in the microcosm experiment. Data points show average values and error bars denote standard deviation.

Supplementary Figure 3.

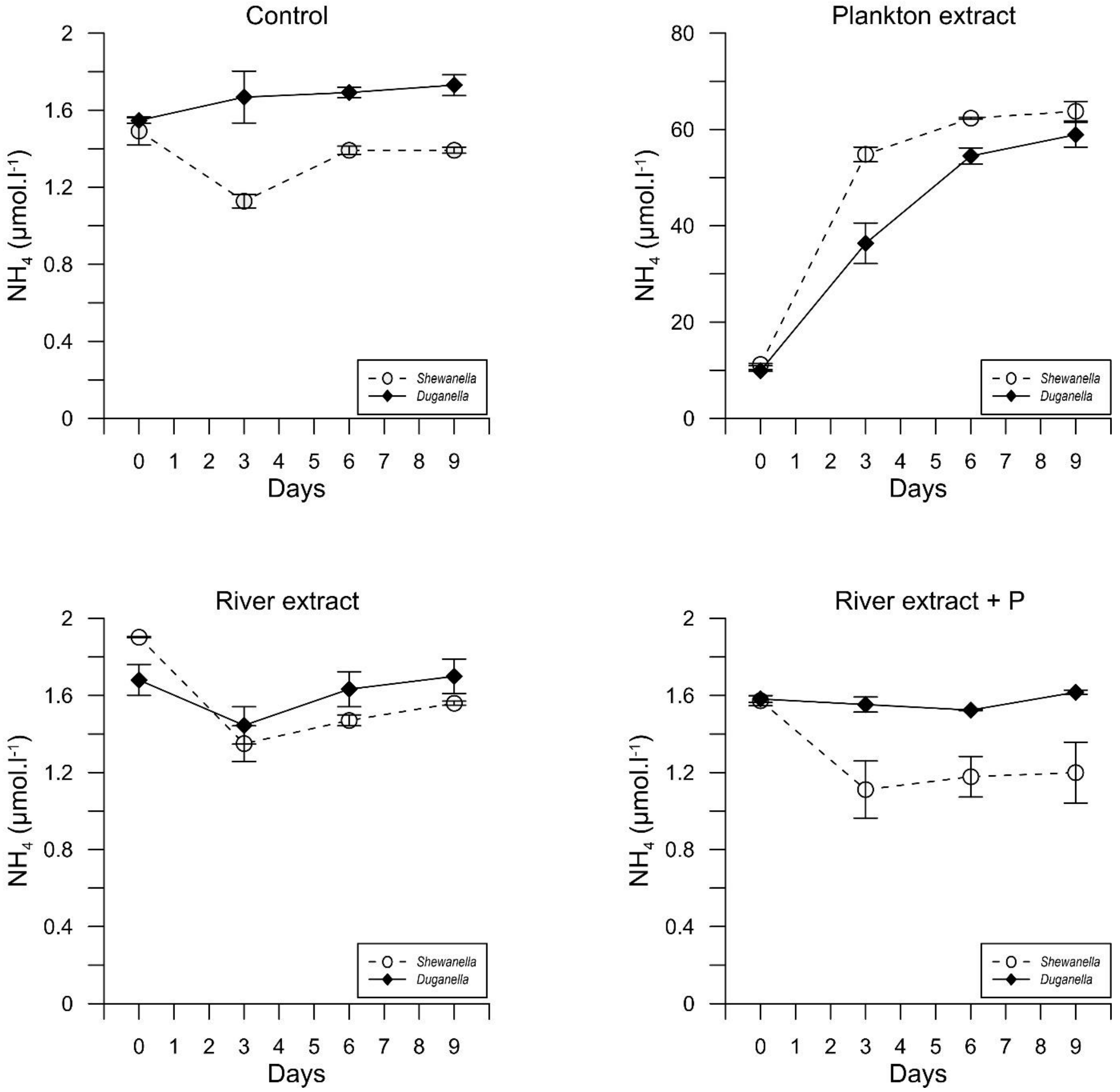

Supplementary Figure 3. Temporal variation of ammonium (NH_4) concentration in the microcosm experiment. Data points show average values and error bars denote standard deviation.

Supplementary Figure 4.

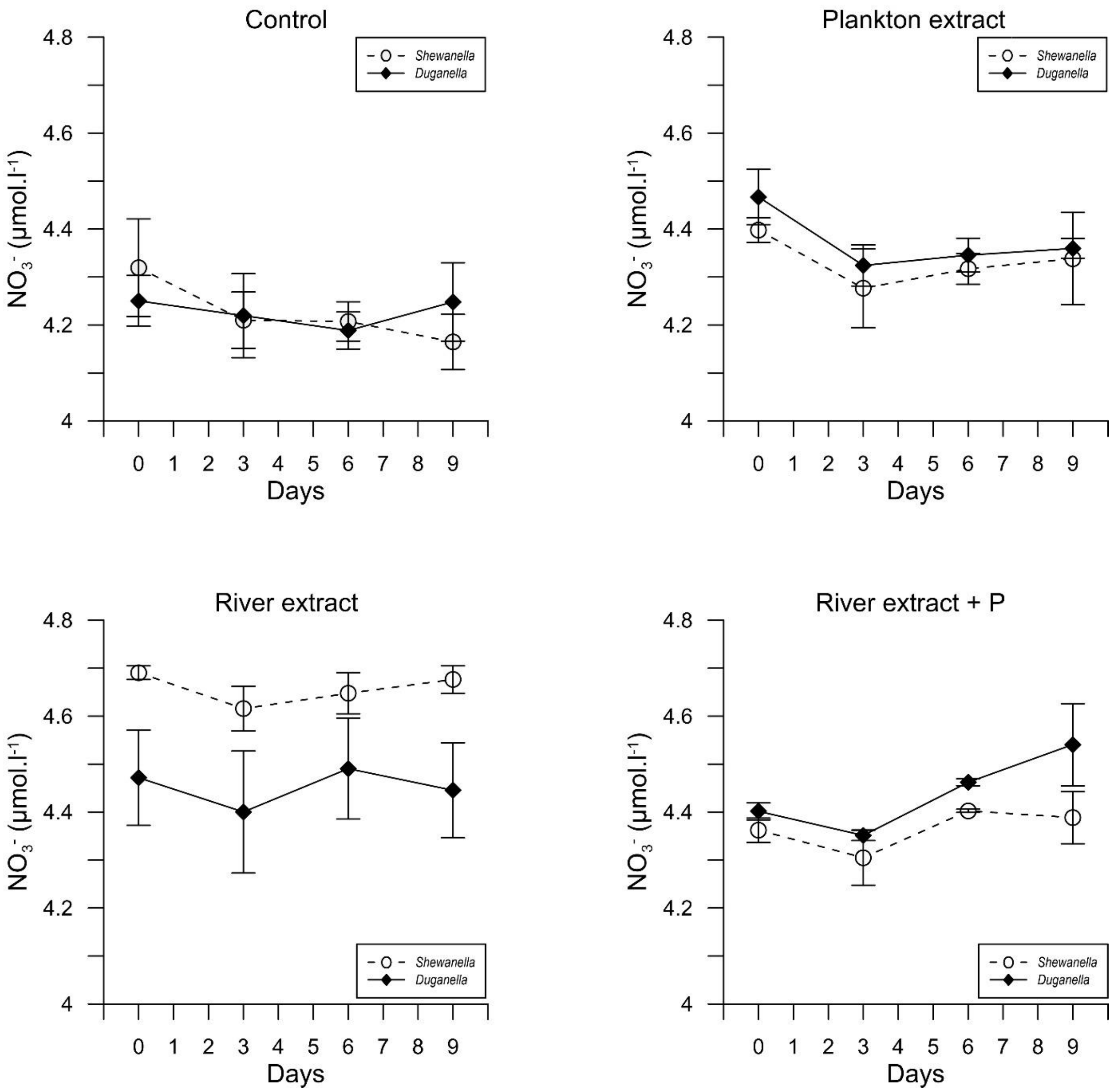

Supplementary Figure 4. Temporal variation of nitrate (NO_3) concentration in the microcosm experiment. Data points show average values and error bars denote standard deviation.

Supplementary Figure 5.

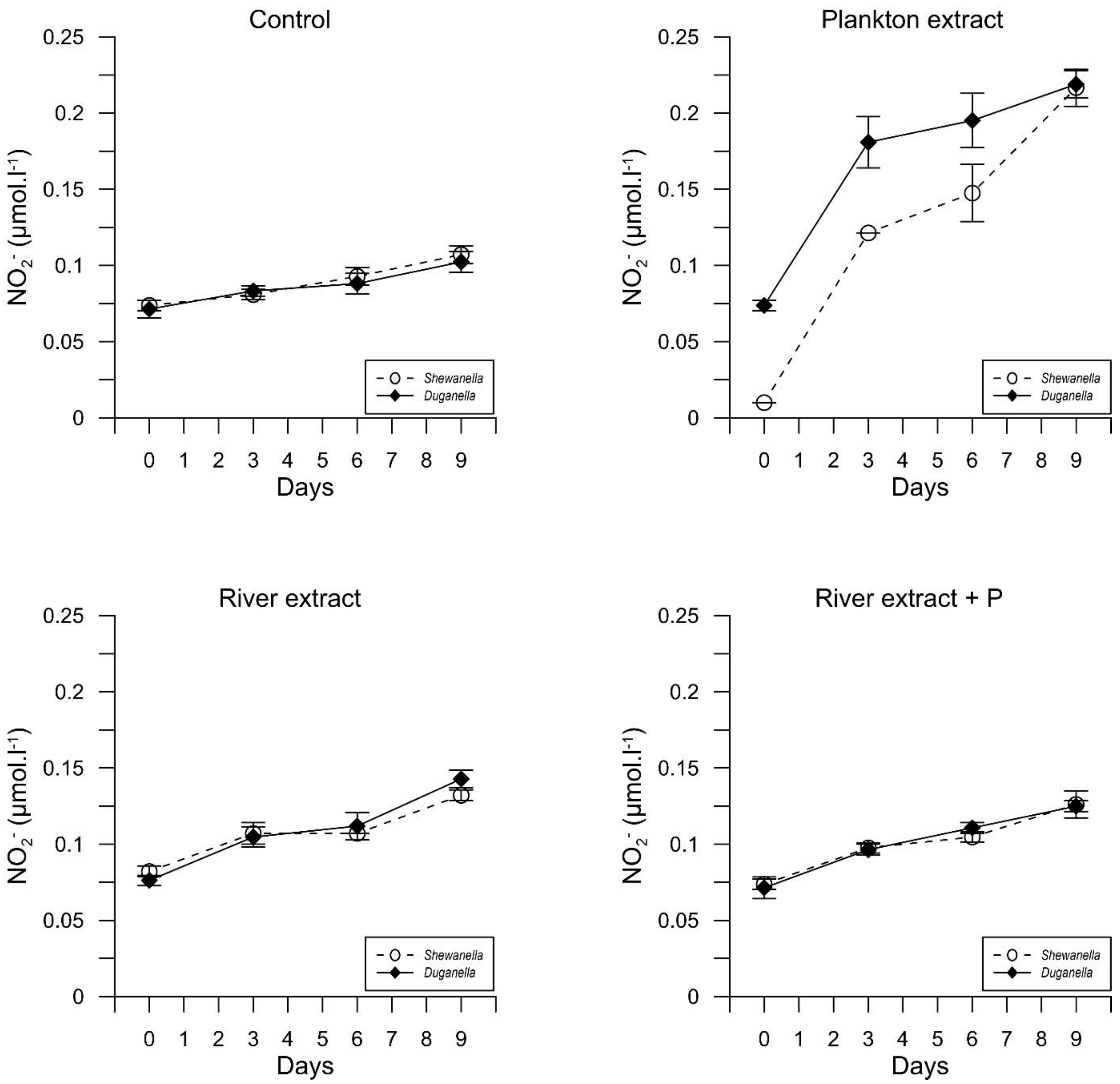

Supplementary Figure 5. Temporal variation of nitrite (NO_2) concentration in the microcosm experiment. Data points show average values and error bars denote standard deviation.

Supplementary Figure 6.

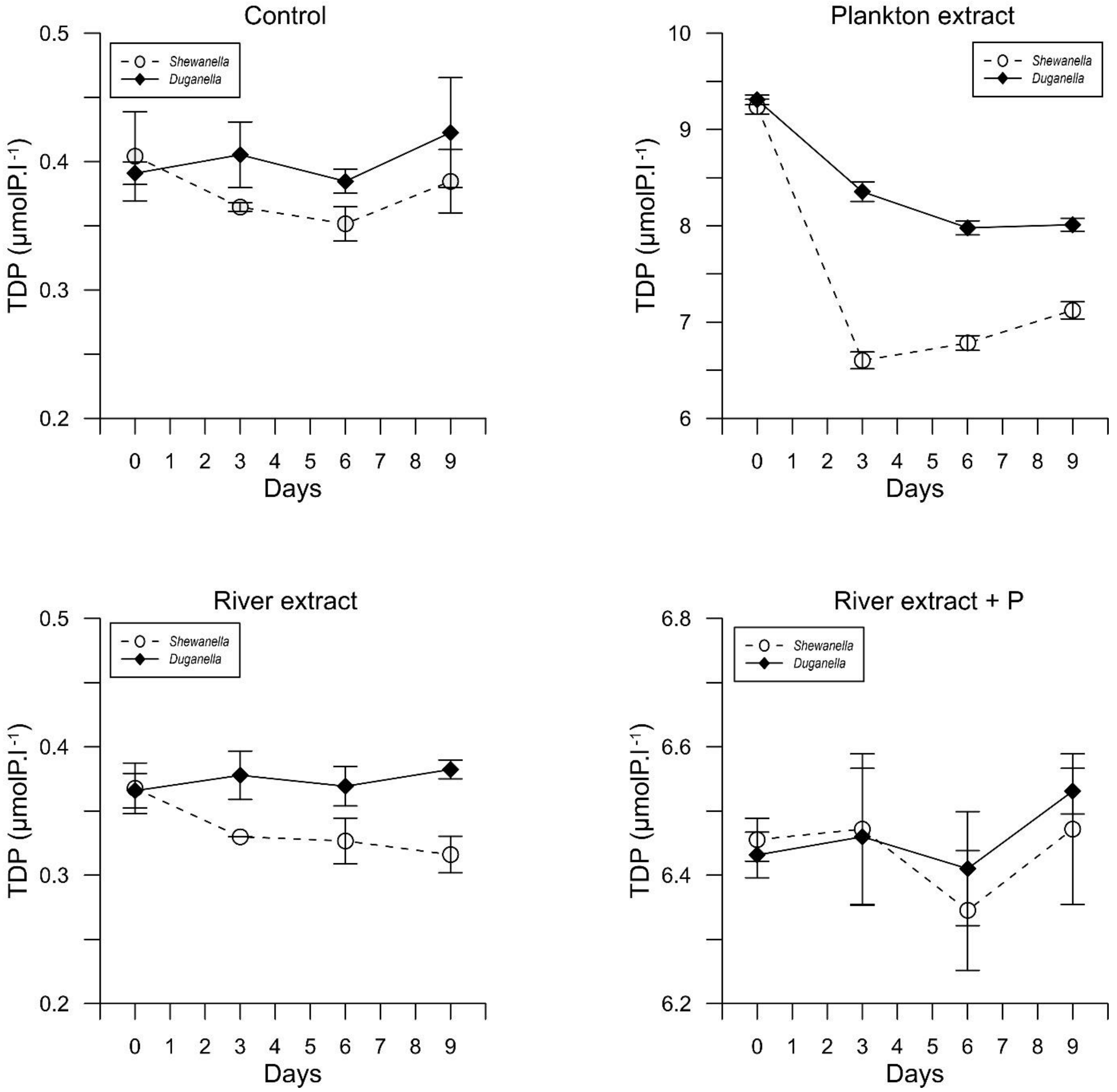

Supplementary Figure 6. Temporal variation of total dissolved phosphorus (TDP) concentration in the microcosm experiment. Data points show average values and error bars denote standard deviation.

Supplementary Figure 7.

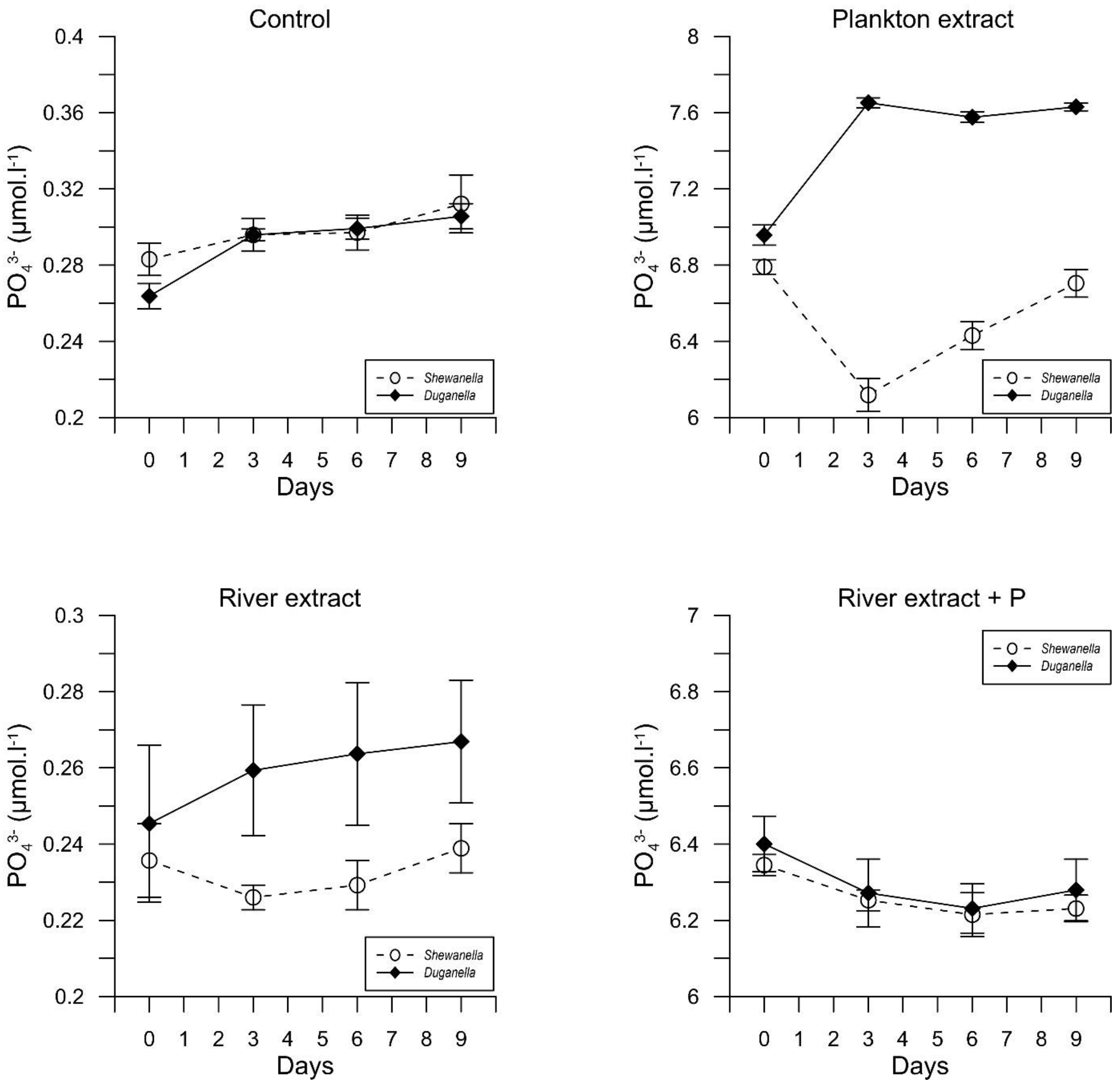

Supplementary Figure 7. Temporal variation of phosphate (PO_4) concentration in the microcosm experiment. Data points show average values and error bars denote standard deviation.

Supplementary Figure 8.

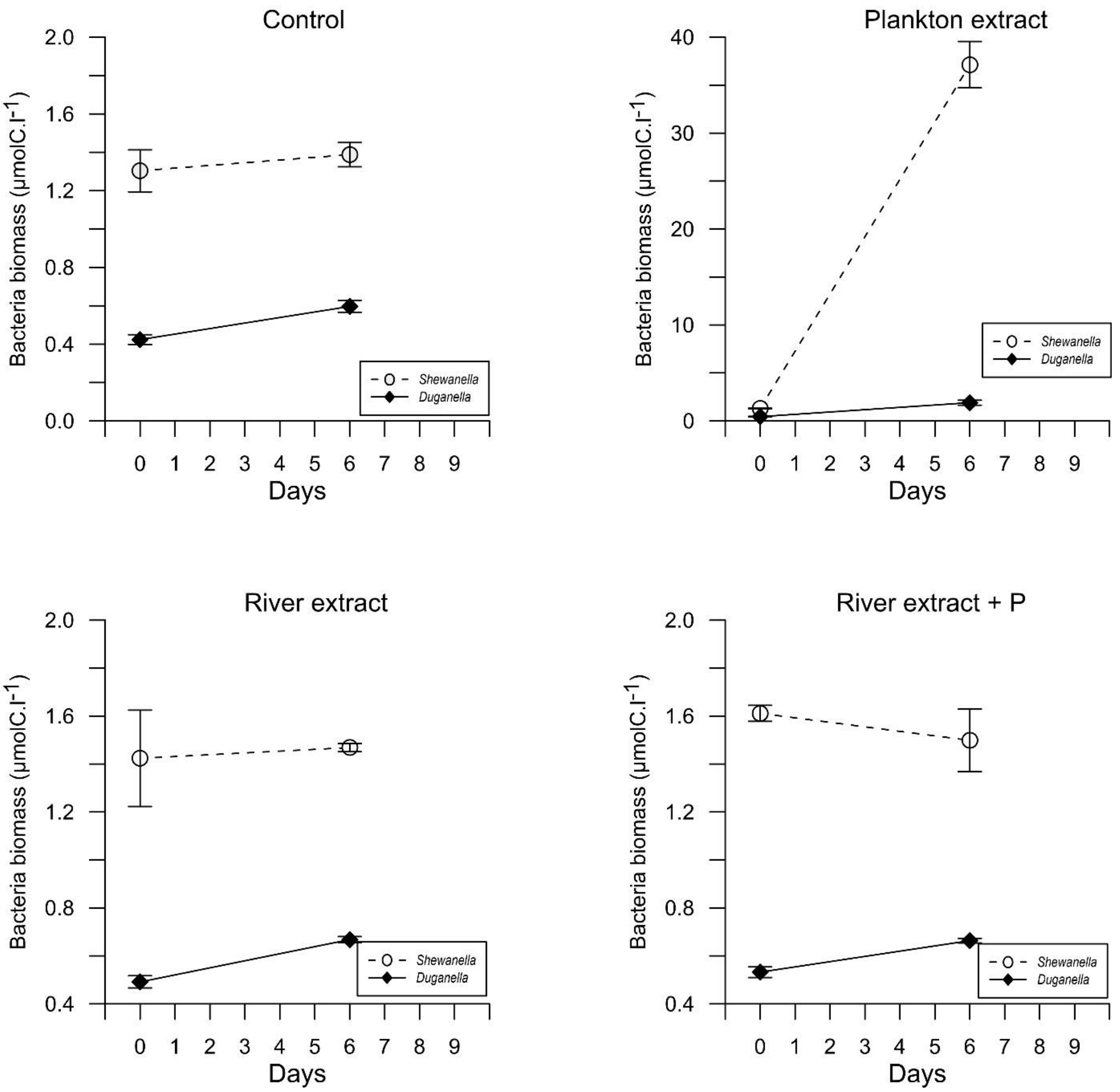

Supplementary Figure 8. Temporal variation of the biomass of *Shewanella* and *Duganella* in the microcosm experiment. Data points show average values and error bars denote standard deviation.

Supplementary Figure 9.

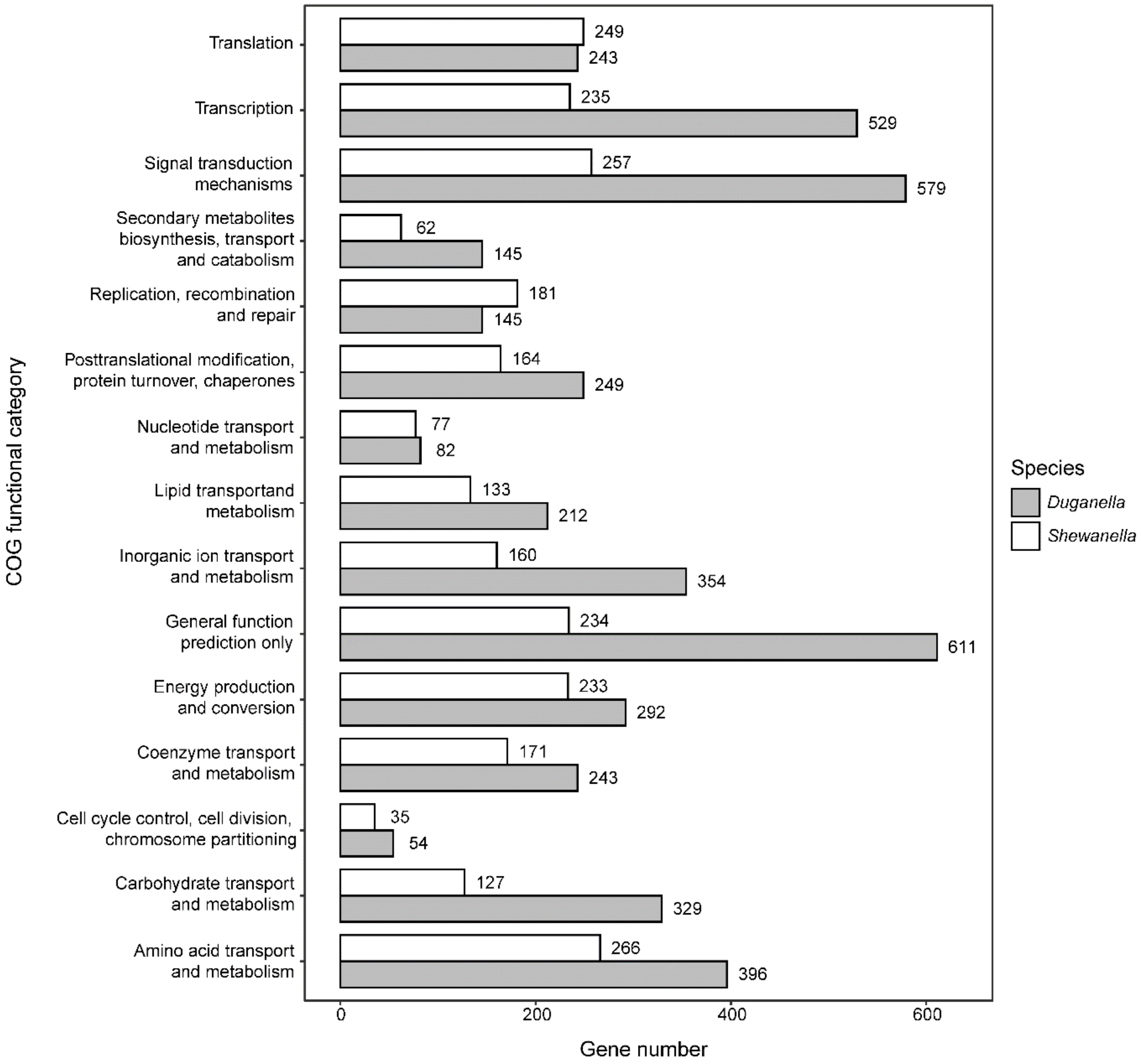

Supplementary Figure 9. Distribution of Clusters of Orthologous Groups of proteins (COGs) functional category of *Shewanella* sp. and *Duganella* sp. genome extracted from IMG (img.jgi.doe.gov).
