# Supplementary material for: Response of Coastal Shewanella and Duganella Bacteria to Planktonic and Terrestrial Food Substrates
Source: Front Microbiol. 2022 Feb 16;12:726844. doi: 10.3389/fmicb.2021.726844 (PMC8888917; doi:10.3389/fmicb.2021.726844)
Supplement: Supplementary file 2 [file Data_Sheet_1.pdf]

Supplementary File 10. Complete comparative abundance list of predicted protein families in *Shewanella baltica* and *Duganella* sp. Protein families discussed in our study are highlighted in yellow.

Function Id	Function Name	*Shewanella baltica* OS117 (ID-651053066)	*Duganella* sp AF9R3 (ID-2884672076)
pfam00004	ATPase family associated with various cellular activities (AAA)	10	10
pfam00005	ABC transporter	44	79
pfam00006	ATP synthase alpha/beta family, nucleotide-binding domain	7	5
pfam00009	Elongation factor Tu GTP binding domain	9	10
pfam00011	Hsp20/alpha crystallin family	1	1
pfam00012	Hsp70 protein	3	6
pfam00013	KH domain	1	1
pfam00015	Methyl-accepting chemotaxis protein (MCP) signalling domain	42	55
pfam00023	Ankyrin repeat	1	0
pfam00027	Cyclic nucleotide-binding domain	4	11
pfam00030	Beta/Gamma crystallin	0	2
pfam00032	Cytochrome b(C-terminal)/b6/petD	1	1
pfam00033	Cytochrome b/b6/petB	1	1
pfam00034	Cytochrome c	5	18
pfam00035	Double-stranded RNA binding motif	1	1
pfam00037	4Fe-4S binding domain	1	1
pfam00041	Fibronectin type III domain	1	0
pfam00042	Globin	1	1
pfam00043	Glutathione S-transferase, C-terminal domain	5	13
pfam00044	Glyceraldehyde 3-phosphate dehydrogenase, NAD binding domain	4	1
pfam00056	lactate/malate dehydrogenase, NAD binding domain	1	1
pfam00067	**Cytochrome P450**	**0**	**2**
pfam00069	Protein kinase domain	2	5
pfam00072	Response regulator receiver domain	62	171
pfam00075	RNase H	1	1
pfam00076	RNA recognition motif. (a.k.a. RRM, RBD, or RNP domain)	1	0
pfam00078	Reverse transcriptase (RNA-dependent DNA polymerase)	4	2
pfam00080	Copper/zinc superoxide dismutase (SODC)	0	1
pfam00081	Iron/manganese superoxide dismutases, alpha-hairpin domain	1	1

pfam00082	Subtilase family	4	4
pfam00083	Sugar (and other) transporter	0	6
pfam00085	Thioredoxin	3	5
pfam00089	Trypsin	1	0
pfam00091	Tubulin/FtsZ family, GTPase domain	1	1
pfam00092	von Willebrand factor type A domain	3	1
pfam00106	short chain dehydrogenase	8	23
pfam00107	Zinc-binding dehydrogenase	6	12
pfam00108	Thiolase, N-terminal domain	3	7
pfam00109	Beta-ketoacyl synthase, N-terminal domain	6	2
pfam00111	2Fe-2S iron-sulfur cluster binding domain	7	10
pfam00113	Enolase, C-terminal TIM barrel domain	1	1
pfam00114	Pilin (bacterial filament)	0	1
pfam00115	Cytochrome C and Quinol oxidase polypeptide I	3	4
pfam00116	Cytochrome C oxidase subunit II, periplasmic domain	1	3
pfam00117	Glutamine amidotransferase class-I	6	6
pfam00118	TCP-1/cpn60 chaperonin family	1	1
pfam00119	ATP synthase A chain	1	1
pfam00120	Glutamine synthetase, catalytic domain	3	3
pfam00121	Triosephosphate isomerase	1	1
pfam00122	E1-E2 ATPase	2	3
pfam00126	Bacterial regulatory helix-turn-helix protein, lysR family	54	106
pfam00127	Copper binding proteins, plastocyanin/azurin family	1	0
pfam00128	Alpha amylase, catalytic domain	6	6
pfam00132	Bacterial transferase hexapeptide (six repeats)	10	12
pfam00133	tRNA synthetases class I (I, L, M and V)	3	3
pfam00135	Carboxylesterase family	0	2
pfam00136	DNA polymerase family B	1	1
pfam00137	ATP synthase subunit C	1	1
pfam00140	Sigma-70 factor, region 1.2	3	4
pfam00141	Peroxidase	1	1
pfam00144	Beta-lactamase	2	10
pfam00146	NADH dehydrogenase	0	1
pfam00149	Calcineurin-like phosphoesterase	9	8

pfam00150	Cellulase (glycosyl hydrolase family 5)	0	3
pfam00152	tRNA synthetases class II (D, K and N)	3	2
pfam00154	recA bacterial DNA recombination protein	1	1
pfam00155	Aminotransferase class I and II	12	21
pfam00156	Phosphoribosyl transferase domain	7	6
pfam00158	Sigma-54 interaction domain	12	9
pfam00160	Cyclophilin type peptidyl-prolyl cis-trans isomerase/CLD	4	3
pfam00162	Phosphoglycerate kinase	1	1
pfam00163	Ribosomal protein S4/S9 N-terminal domain	1	1
pfam00164	Ribosomal protein S12/S23	1	1
pfam00166	Chaperonin 10 Kd subunit	1	1
pfam00171	Aldehyde dehydrogenase family	13	15
pfam00174	Oxidoreductase molybdopterin binding domain	2	3
pfam00175	Oxidoreductase NAD-binding domain	7	6
pfam00176	SNF2 family N-terminal domain	2	3
pfam00177	Ribosomal protein S7p/S5e	1	1
pfam00180	Isocitrate/isopropylmalate dehydrogenase	2	4
pfam00181	Ribosomal Proteins L2, RNA binding domain	1	1
pfam00183	Hsp90 protein	1	1
pfam00185	Aspartate/ornithine carbamoyltransferase, Asp/Orn binding domain	2	2
pfam00186	Dihydrofolate reductase	1	1
pfam00188	Cysteine-rich secretory protein family	0	2
pfam00189	Ribosomal protein S3, C-terminal domain	1	1
pfam00190	Cupin	0	1
pfam00194	Eukaryotic-type carbonic anhydrase	0	1
pfam00196	Bacterial regulatory proteins, luxR family	11	30
pfam00198	2-oxoacid dehydrogenases acyltransferase (catalytic domain)	3	2
pfam00199	Catalase	2	7
pfam00202	Aminotransferase class-III	5	6
pfam00203	Ribosomal protein S19	1	1
pfam00204	DNA gyrase B	2	2
pfam00205	Thiamine pyrophosphate enzyme, central domain	3	3
pfam00206	Lyase	4	4
pfam00207	Alpha-2-macroglobulin family	1	1

pfam00208	Glutamate/Leucine/Phenylalanine/Valine dehydrogenase	1	1
pfam00209	Sodium:neurotransmitter symporter family	1	1
pfam00210	Ferritin-like domain	4	3
pfam00211	Adenylate and Guanylate cyclase catalytic domain	1	2
pfam00213	ATP synthase delta (OSCP) subunit	1	1
pfam00215	Orotidine 5'-phosphate decarboxylase / HUMPS family	1	1
pfam00216	Bacterial DNA-binding protein	5	4
pfam00218	Indole-3-glycerol phosphate synthase	1	1
pfam00221	Aromatic amino acid lyase	2	1
pfam00224	Pyruvate kinase, barrel domain	1	2
pfam00226	DnaJ domain	4	5
pfam00227	Proteasome subunit	1	1
pfam00230	Major intrinsic protein	1	0
pfam00231	ATP synthase	1	1
pfam00232	Glycosyl hydrolase family 1	1	4
pfam00237	Ribosomal protein L22p/L17e	1	1
pfam00238	Ribosomal protein L14p/L23e	1	1
pfam00239	Resolvase, N terminal domain	8	5
pfam00245	Alkaline phosphatase	3	0
pfam00246	Zinc carboxypeptidase	3	5
pfam00248	Aldo/keto reductase family	2	13
pfam00252	Ribosomal protein L16p/L10e	1	1
pfam00253	Ribosomal protein S14p/S29e	1	1
pfam00254	FKBP-type peptidyl-prolyl cis-trans isomerase	8	8
pfam00255	Glutathione peroxidase	2	3
pfam00258	Flavodoxin	4	2
pfam00263	Bacterial type II and III secretion system protein	6	8
pfam00265	Thymidine kinase	1	1
pfam00266	Aminotransferase class-V	5	5
pfam00267	Gram-negative porin	2	0
pfam00268	Ribonucleotide reductase, small chain	1	1
pfam00270	DEAD/DEAH box helicase	21	16
pfam00271	Helicase conserved C-terminal domain	26	21
pfam00275	EPSP synthase (3-phosphoshikimate 1-carboxyvinyltransferase)	2	2

pfam00276	Ribosomal protein L23	1	1
pfam00278	Pyridoxal-dependent decarboxylase, C-terminal sheet domain	3	1
pfam00281	Ribosomal protein L5	1	1
pfam00282	Pyridoxal-dependent decarboxylase conserved domain	1	1
pfam00285	Citrate synthase, C-terminal domain	2	3
pfam00288	GHMP kinases N terminal domain	3	1
pfam00289	Biotin carboxylase, N-terminal domain	2	2
pfam00290	Tryptophan synthase alpha chain	1	1
pfam00291	Pyridoxal-phosphate dependent enzyme	7	7
pfam00293	NUDIX domain	8	9
pfam00294	pfkB family carbohydrate kinase	5	7
pfam00295	Glycosyl hydrolases family 28	0	2
pfam00296	Luciferase-like monooxygenase	1	7
pfam00297	Ribosomal protein L3	1	1
pfam00298	Ribosomal protein L11, RNA binding domain	1	1
pfam00300	Histidine phosphatase superfamily (branch 1)	3	6
pfam00301	Rubredoxin	0	1
pfam00302	Chloramphenicol acetyltransferase	0	1
pfam00303	Thymidylate synthase	1	1
pfam00306	ATP synthase alpha/beta chain, C terminal domain	1	1
pfam00308	Bacterial dnaA protein	2	2
pfam00309	Sigma-54 factor, Activator interacting domain (AID)	1	1
pfam00310	Glutamine amidotransferases class-II	1	1
pfam00311	Phosphoenolpyruvate carboxylase	1	1
pfam00312	Ribosomal protein S15	1	1
pfam00313	'Cold-shock' DNA-binding domain	4	3
pfam00316	Fructose-1-6-bisphosphatase, N-terminal domain	1	1
pfam00317	Ribonucleotide reductase, all-alpha domain	1	1
pfam00318	Ribosomal protein S2	1	1
pfam00324	Amino acid permease	0	1
pfam00325	Bacterial regulatory proteins, crp family	1	0
pfam00326	Prolyl oligopeptidase family	10	18
pfam00327	Ribosomal protein L30p/L7e	1	1
pfam00328	Histidine phosphatase superfamily (branch 2)	0	1

pfam00329	Respiratory-chain NADH dehydrogenase, 30 Kd subunit	0	1
pfam00330	Aconitase family (aconitate hydratase)	3	4
pfam00331	Glycosyl hydrolase family 10	0	1
pfam00333	Ribosomal protein S5, N-terminal domain	1	1
pfam00334	Nucleoside diphosphate kinase	1	1
pfam00338	Ribosomal protein S10p/S20e	1	1
pfam00342	Phosphoglucose isomerase	1	1
pfam00343	**Carbohydrate phosphorylase**	**1**	**0**
pfam00344	SecY translocase	1	1
pfam00345	Pili and flagellar-assembly chaperone, PapD N-terminal domain	0	1
pfam00346	Respiratory-chain NADH dehydrogenase, 49 Kd subunit	0	1
pfam00347	Ribosomal protein L6	1	1
pfam00348	Polyprenyl synthetase	2	2
pfam00350	Dynamin family	0	1
pfam00351	Biopterin-dependent aromatic amino acid hydroxylase	1	0
pfam00353	Haemolysin-type calcium-binding repeat (2 copies)	1	0
pfam00355	Rieske [2Fe-2S] domain	1	5
pfam00356	Bacterial regulatory proteins, lacI family	5	17
pfam00358	phosphoenolpyruvate-dependent sugar phosphotransferase system, EIIA	1	0
pfam00359	Phosphoenolpyruvate-dependent sugar phosphotransferase system, EIIA	1	1
pfam00360	Phytochrome region	0	2
pfam00361	Proton-conducting membrane transporter	0	3
pfam00364	Biotin-requiring enzyme	6	5
pfam00365	Phosphofructokinase	0	1
pfam00366	Ribosomal protein S17	1	1
pfam00367	phosphotransferase system, EIIB	1	0
pfam00370	FGGY family of carbohydrate kinases, N-terminal domain	1	2
pfam00374	Nickel-dependent hydrogenase	1	0
pfam00375	Sodium:dicarboxylate symporter family	7	5
pfam00376	MerR family regulatory protein	1	4
pfam00378	Enoyl-CoA hydratase/isomerase	7	9
pfam00380	Ribosomal protein S9/S16	1	1
pfam00381	PTS HPr component phosphorylation site	2	1
pfam00383	Cytidine and deoxycytidylate deaminase zinc-binding region	4	4

pfam00384	Molybdopterin oxidoreductase	8	7
pfam00389	D-isomer specific 2-hydroxyacid dehydrogenase, catalytic domain	4	5
pfam00390	Malic enzyme, N-terminal domain	2	2
pfam00391	PEP-utilising enzyme, mobile domain	3	2
pfam00392	Bacterial regulatory proteins, gntR family	11	18
pfam00393	6-phosphogluconate dehydrogenase, C-terminal domain	1	1
pfam00398	Ribosomal RNA adenine dimethylase	1	1
pfam00400	WD domain, G-beta repeat	1	0
pfam00401	ATP synthase, Delta/Epsilon chain, long alpha-helix domain	1	1
pfam00403	Heavy-metal-associated domain	3	3
pfam00406	Adenylate kinase	1	1
pfam00408	Phosphoglucomutase/phosphomannomutase, C-terminal domain	4	3
pfam00410	Ribosomal protein S8	1	1
pfam00411	Ribosomal protein S11	1	1
pfam00413	Matrixin	0	1
pfam00415	Regulator of chromosome condensation (RCC1) repeat	0	1
pfam00416	Ribosomal protein S13/S18	1	1
pfam00420	NADH-ubiquinone/plastoquinone oxidoreductase chain 4L	0	1
pfam00425	chorismate binding enzyme	3	2
pfam00430	ATP synthase B/B' CF(0)	1	1
pfam00436	Single-strand binding protein family	4	1
pfam00437	Type II/IV secretion system protein	6	14
pfam00438	S-adenosylmethionine synthetase, N-terminal domain	1	1
pfam00440	Bacterial regulatory proteins, tetR family	14	32
pfam00441	Acyl-CoA dehydrogenase, C-terminal domain	6	13
pfam00444	Ribosomal protein L36	1	0
pfam00445	Ribonuclease T2 family	1	1
pfam00448	SRP54-type protein, GTPase domain	3	3
pfam00449	Urease alpha-subunit, N-terminal domain	0	1
pfam00450	Serine carboxypeptidase	1	1
pfam00453	Ribosomal protein L20	1	1
pfam00455	DeoR C terminal sensor domain	1	3
pfam00456	Transketolase, thiamine diphosphate binding domain	2	3
pfam00459	Inositol monophosphatase family	3	3

pfam00460	Flagella basal body rod protein	10	12
pfam00462	Glutaredoxin	2	4
pfam00463	Isocitrate lyase family	1	1
pfam00464	Serine hydroxymethyltransferase	1	1
pfam00465	Iron-containing alcohol dehydrogenase	5	1
pfam00466	Ribosomal protein L10	1	1
pfam00467	KOW motif	1	1
pfam00468	Ribosomal protein L34	1	1
pfam00471	Ribosomal protein L33	1	1
pfam00472	RF-1 domain	3	3
pfam00474	Sodium:solute symporter family	3	4
pfam00475	Imidazoleglycerol-phosphate dehydratase	1	1
pfam00476	DNA polymerase family A	1	1
pfam00478	IMP dehydrogenase / GMP reductase domain	1	1
pfam00479	Glucose-6-phosphate dehydrogenase, NAD binding domain	1	1
pfam00480	ROK family	4	3
pfam00482	Type II secretion system (T2SS), protein F	3	11
pfam00483	Nucleotidyl transferase	5	6
pfam00484	Carbonic anhydrase	1	1
pfam00485	Phosphoribulokinase / Uridine kinase family	3	1
pfam00486	Transcriptional regulatory protein, C terminal	20	30
pfam00487	Fatty acid desaturase	1	2
pfam00488	MutS domain V	1	2
pfam00490	Delta-aminolevulinic acid dehydratase	2	1
pfam00491	Arginase family	1	0
pfam00494	Squalene/phytoene synthase	0	2
pfam00496	Bacterial extracellular solute-binding proteins, family 5 Middle	1	1
pfam00497	Bacterial extracellular solute-binding proteins, family 3	22	24
pfam00498	FHA domain	1	3
pfam00499	NADH-ubiquinone/plastoquinone oxidoreductase chain 6	0	1
pfam00501	AMP-binding enzyme	10	13
pfam00507	NADH-ubiquinone/plastoquinone oxidoreductase, chain 3	0	1
pfam00510	Cytochrome c oxidase subunit III	2	2
pfam00512	His Kinase A (phospho-acceptor) domain	24	82

pfam00520	Ion transport protein	2	1
pfam00521	DNA gyrase/topoisomerase IV, subunit A	2	2
pfam00528	Binding-protein-dependent transport system inner membrane compone	14	33
pfam00529	HlyD membrane-fusion protein of T1SS	2	1
pfam00532	Periplasmic binding proteins and sugar binding domain of LacI family	2	2
pfam00533	BRCA1 C Terminus (BRCT) domain	2	1
pfam00534	Glycosyl transferases group 1	4	5
pfam00535	Glycosyl transferase family 2	6	13
pfam00542	Ribosomal protein L7/L12 C-terminal domain	1	1
pfam00543	Nitrogen regulatory protein P-II	3	2
pfam00544	**Pectate lyase**	**0**	**2**
pfam00547	Urease, gamma subunit	0	1
pfam00549	CoA-ligase	2	2
pfam00550	Phosphopantetheine attachment site	4	1
pfam00551	Formyl transferase	3	5
pfam00557	Metallopeptidase family M24	3	7
pfam00561	alpha/beta hydrolase fold	8	23
pfam00562	RNA polymerase Rpb2, domain 6	1	1
pfam00563	EAL domain	28	43
pfam00570	HRDC domain	2	1
pfam00571	CBS domain	12	11
pfam00572	Ribosomal protein L13	1	1
pfam00573	Ribosomal protein L4/L1 family	1	1
pfam00574	Clp protease	2	3
pfam00575	S1 RNA binding domain	7	6
pfam00576	HIUase/Transthyretin family	0	1
pfam00577	Outer membrane usher protein	0	1
pfam00578	AhpC/TSA family	6	11
pfam00579	tRNA synthetases class I (W and Y)	2	3
pfam00580	UvrD/REP helicase N-terminal domain	4	3
pfam00581	Rhodanese-like domain	7	11
pfam00582	Universal stress protein family	3	3
pfam00583	Acetyltransferase (GNAT) family	22	16
pfam00584	SecE/Sec61-gamma subunits of protein translocation complex	1	1

pfam00585	C-terminal regulatory domain of Threonine dehydratase	1	1
pfam00586	AIR synthase related protein, N-terminal domain	4	3
pfam00587	tRNA synthetase class II core domain (G, H, P, S and T)	4	3
pfam00588	SpoU rRNA Methylase family	4	4
pfam00589	Phage integrase family	18	10
pfam00590	Tetrapyrrole (Corrin/Porphyrin) Methylases	3	7
pfam00591	Glycosyl transferase family, a/b domain	2	3
pfam00593	TonB dependent receptor	28	66
pfam00595	PDZ domain (Also known as DHR or GLGF)	1	2
pfam00596	Class II Aldolase and Adducin N-terminal domain	0	1
pfam00623	RNA polymerase Rpb1, domain 2	1	1
pfam00625	Guanylate kinase	1	1
pfam00633	Helix-hairpin-helix motif	2	0
pfam00639	PPIC-type PPIASE domain	1	1
pfam00654	Voltage gated chloride channel	1	2
pfam00656	Caspase domain	0	2
pfam00657	GDSL-like Lipase/Acylhydrolase	1	0
pfam00662	NADH-Ubiquinone oxidoreductase (complex I), chain 5 N-terminus	0	1
pfam00664	**ABC transporter transmembrane region**	**7**	**8**
pfam00665	Integrase core domain	114	1
pfam00667	FAD binding domain	1	0
pfam00669	Bacterial flagellin N-terminal helical region	8	6
pfam00670	S-adenosyl-L-homocysteine hydrolase, NAD binding domain	0	1
pfam00672	HAMP domain	39	60
pfam00673	ribosomal L5P family C-terminus	1	1
pfam00675	Insulinase (Peptidase family M16)	6	5
pfam00676	Dehydrogenase E1 component	2	1
pfam00677	Lumazine binding domain	2	1
pfam00679	Elongation factor G C-terminus	4	4
pfam00682	HMGL-like	3	3
pfam00684	DnaJ central domain	1	1
pfam00687	Ribosomal protein L1p/L10e family	1	1
pfam00691	OmpA family	13	11
pfam00692	dUTPase	2	2

pfam00694	Aconitase C-terminal domain	2	3
pfam00696	Amino acid kinase family	9	5
pfam00697	N-(5'phosphoribosyl)anthranilate (PRA) isomerase	1	1
pfam00698	Acyl transferase domain	2	2
pfam00699	Urease beta subunit	0	1
pfam00700	Bacterial flagellin C-terminal helical region	7	4
pfam00701	Dihydrodipicolinate synthetase family	1	5
pfam00702	haloacid dehalogenase-like hydrolase	2	3
pfam00703	Glycosyl hydrolases family 2	0	3
pfam00704	Glycosyl hydrolases family 18	2	1
pfam00707	Translation initiation factor IF-3, C-terminal domain	1	1
pfam00708	Acylphosphatase	2	1
pfam00709	Adenylosuccinate synthetase	2	1
pfam00710	Asparaginase, N-terminal	2	2
pfam00712	DNA polymerase III beta subunit, N-terminal domain	1	1
pfam00717	Peptidase S24-like	8	5
pfam00719	Inorganic pyrophosphatase	0	1
pfam00722	Glycosyl hydrolases family 16	0	1
pfam00724	NADH:flavin oxidoreductase / NADH oxidase family	5	11
pfam00725	3-hydroxyacyl-CoA dehydrogenase, C-terminal domain	2	3
pfam00728	Glycosyl hydrolase family 20, catalytic domain	2	1
pfam00730	HhH-GPD superfamily base excision DNA repair protein	3	4
pfam00731	AIR carboxylase	1	1
pfam00732	GMC oxidoreductase	3	6
pfam00733	Asparagine synthase	1	0
pfam00742	Homoserine dehydrogenase	2	1
pfam00743	Flavin-binding monooxygenase-like	0	1
pfam00745	Glutamyl-tRNAGlu reductase, dimerisation domain	1	1
pfam00749	tRNA synthetases class I (E and Q), catalytic domain	3	3
pfam00750	tRNA synthetases class I (R)	1	1
pfam00753	Metallo-beta-lactamase superfamily	5	12
pfam00754	F5/8 type C domain	0	3
pfam00756	Putative esterase	2	2
pfam00759	Glycosyl hydrolase family 9	0	1

pfam00762	Ferrochelatase	2	1
pfam00763	Tetrahydrofolate dehydrogenase/cyclohydrolase, catalytic domain	1	1
pfam00764	Arginosuccinate synthase	1	1
pfam00766	Electron transfer flavoprotein FAD-binding domain	1	1
pfam00768	D-alanyl-D-alanine carboxypeptidase	2	3
pfam00771	FHIPEP family	4	2
pfam00772	DnaB-like helicase N terminal domain	1	1
pfam00773	RNB domain	1	2
pfam00775	**Dioxygenase**	**0**	**1**
pfam00781	Diacylglycerol kinase catalytic domain	1	1
pfam00782	Dual specificity phosphatase, catalytic domain	2	0
pfam00793	DAHP synthetase I family	5	3
pfam00795	**Carbon-nitrogen hydrolase**	**2**	**4**
pfam00800	Prephenate dehydratase	1	1
pfam00801	PKD domain	3	0
pfam00809	Pterin binding enzyme	2	2
pfam00813	FliP family	4	2
pfam00814	Glycoprotease family	2	2
pfam00815	Histidinol dehydrogenase	1	1
pfam00816	H-NS histone family	2	4
pfam00817	impB/mucB/samB family	3	3
pfam00821	Phosphoenolpyruvate carboxykinase	0	1
pfam00825	Ribonuclease P	1	1
pfam00828	Ribosomal proteins 50S-L15, 50S-L18e, 60S-L27A	1	1
pfam00829	Ribosomal prokaryotic L21 protein	1	1
pfam00830	Ribosomal L28 family	1	1
pfam00831	Ribosomal L29 protein	1	1
pfam00834	Ribulose-phosphate 3 epimerase family	1	1
pfam00842	Alanine racemase, C-terminal domain	1	1
pfam00848	Ring hydroxylating alpha subunit (catalytic domain)	0	1
pfam00849	RNA pseudouridylate synthase	15	7
pfam00850	Histone deacetylase domain	1	2
pfam00854	POT family	3	2
pfam00856	SET domain	0	1

pfam00857	Isochorismatase family	2	9
pfam00860	Permease family	3	1
pfam00861	Ribosomal L18 of archaea, bacteria, mitoch. and chloroplast	1	1
pfam00871	Acetokinase family	1	1
pfam00872	Transposase, Mutator family	1	0
pfam00873	AcrB/AcrD/AcrF family	15	16
pfam00875	DNA photolyase	1	1
pfam00877	NlpC/P60 family	1	0
pfam00881	Nitroreductase family	2	5
pfam00883	Cytosol aminopeptidase family, catalytic domain	4	1
pfam00884	Sulfatase	4	5
pfam00885	6,7-dimethyl-8-ribityllumazine synthase	1	1
pfam00886	Ribosomal protein S16	1	1
pfam00887	Acyl CoA binding protein	0	1
pfam00889	Elongation factor TS	1	1
pfam00890	FAD binding domain	7	2
pfam00892	EamA-like transporter family	14	19
pfam00893	Small Multidrug Resistance protein	1	2
pfam00899	ThiF family	3	3
pfam00902	Sec-independent protein translocase protein (TatC)	1	1
pfam00903	Glyoxalase/Bleomycin resistance protein/Dioxygenase superfamily	7	11
pfam00905	Penicillin binding protein transpeptidase domain	7	9
pfam00908	dTDP-4-dehydrorhamnose 3,5-epimerase	2	1
pfam00909	Ammonium Transporter Family	2	2
pfam00912	Transglycosylase	5	5
pfam00916	Sulfate permease family	3	2
pfam00919	Uncharacterized protein family UPF0004	2	1
pfam00920	Dehydratase family	2	4
pfam00923	Transaldolase/Fructose-6-phosphate aldolase	1	2
pfam00924	Mechanosensitive ion channel	6	3
pfam00925	GTP cyclohydrolase II	2	2
pfam00926	3,4-dihydroxy-2-butanone 4-phosphate synthase	2	2
pfam00929	Exonuclease	7	4
pfam00930	Dipeptidyl peptidase IV (DPP IV) N-terminal region	2	2

pfam00931	NB-ARC domain	0	1
pfam00932	Lamin Tail Domain	3	1
pfam00933	Glycosyl hydrolase family 3 N terminal domain	2	6
pfam00939	Sodium:sulfate symporter transmembrane region	3	1
pfam00941	FAD binding domain in molybdopterin dehydrogenase	0	5
pfam00950	ABC 3 transport family	1	0
pfam00953	Glycosyl transferase family 4	1	2
pfam00958	GMP synthase C terminal domain	1	1
pfam00959	Phage lysozyme	2	0
pfam00962	Adenosine/AMP deaminase	1	2
pfam00970	Oxidoreductase FAD-binding domain	5	4
pfam00977	Histidine biosynthesis protein	2	2
pfam00984	UDP-glucose/GDP-mannose dehydrogenase family, central domain	1	1
pfam00986	DNA gyrase B subunit, carboxyl terminus	2	2
pfam00988	Carbamoyl-phosphate synthase small chain, CPSase domain	1	1
pfam00989	PAS fold	5	18
pfam00990	Diguanylate cyclase, GGDEF domain	58	74
pfam00994	Probable molybdopterin binding domain	4	5
pfam00999	Sodium/hydrogen exchanger family	6	8
pfam01000	RNA polymerase Rpb3/RpoA insert domain	1	1
pfam01012	Electron transfer flavoprotein domain	2	2
pfam01016	Ribosomal L27 protein	1	1
pfam01018	GTP1/OBG	1	1
pfam01019	Gamma-glutamyltranspeptidase	2	2
pfam01022	Bacterial regulatory protein, arsR family	3	4
pfam01025	GrpE	1	1
pfam01026	TatD related DNase	3	2
pfam01027	Inhibitor of apoptosis-promoting Bax1	2	1
pfam01028	Eukaryotic DNA topoisomerase I, catalytic core	0	1
pfam01029	NusB family	2	1
pfam01032	FecCD transport family	2	1
pfam01035	6-O-methylguanine DNA methyltransferase, DNA binding domain	3	3
pfam01037	Lrp/AsnC ligand binding domain	4	6
pfam01039	Carboxyl transferase domain	2	3

pfam01040	UbiA prenyltransferase family	4	3
pfam01041	DegT/DnrJ/EryC1/StrS aminotransferase family	2	4
pfam01042	Endoribonuclease L-PSP	3	6
pfam01043	SecA preprotein cross-linking domain	1	1
pfam01047	MarR family	7	10
pfam01048	Phosphorylase superfamily	5	3
pfam01050	Mannose-6-phosphate isomerase	0	1
pfam01051	Initiator Replication protein	2	0
pfam01052	Type III flagellar switch regulator (C-ring) FliN C-term	5	4
pfam01053	Cys/Met metabolism PLP-dependent enzyme	5	5
pfam01055	Glycosyl hydrolases family 31	0	3
pfam01058	NADH ubiquinone oxidoreductase, 20 Kd subunit	1	1
pfam01061	ABC-2 type transporter	1	2
pfam01062	Bestrophin, RFP-TM, chloride channel	0	1
pfam01063	Amino-transferase class IV	2	1
pfam01066	CDP-alcohol phosphatidyltransferase	2	3
pfam01068	ATP dependent DNA ligase domain	1	2
pfam01070	FMN-dependent dehydrogenase	0	1
pfam01071	Phosphoribosylglycinamide synthetase, ATP-grasp (A) domain	1	1
pfam01073	3-beta hydroxysteroid dehydrogenase/isomerase family	1	0
pfam01075	Glycosyltransferase family 9 (heptosyltransferase)	1	3
pfam01077	Nitrite and sulphite reductase 4Fe-4S domain	1	2
pfam01078	Magnesium chelatase, subunit ChlI	1	2
pfam01081	KDPG and KHG aldolase	1	2
pfam01084	Ribosomal protein S18	1	1
pfam01095	Pectinesterase	0	2
pfam01098	Cell cycle protein	2	4
pfam01103	Surface antigen	2	3
pfam01106	NifU-like domain	1	0
pfam01112	Asparaginase	1	2
pfam01113	Dihydrodipicolinate reductase, N-terminus	1	1
pfam01116	Fructose-bisphosphate aldolase class-II	1	1
pfam01117	Aerolysin toxin	1	0
pfam01118	Semialdehyde dehydrogenase, NAD binding domain	2	2

pfam01119	DNA mismatch repair protein, C-terminal domain	1	1
pfam01121	Dephospho-CoA kinase	1	1
pfam01124	MAPEG family	2	0
pfam01126	Heme oxygenase	0	1
pfam01127	Succinate dehydrogenase/Fumarate reductase transmembrane subunit	5	2
pfam01128	2-C-methyl-D-erythritol 4-phosphate cytidylyltransferase	1	1
pfam01131	DNA topoisomerase	4	1
pfam01132	Elongation factor P (EF-P) OB domain	1	1
pfam01134	Glucose inhibited division protein A	1	1
pfam01135	Protein-L-isoaspartate(D-aspartate) O-methyltransferase (PCMT)	1	2
pfam01136	Peptidase family U32	4	4
pfam01138	3' exoribonuclease family, domain 1	2	2
pfam01142	tRNA pseudouridine synthase D (TruD)	1	0
pfam01144	Coenzyme A transferase	2	3
pfam01145	SPFH domain / Band 7 family	6	4
pfam01148	Cytidylyltransferase family	1	1
pfam01149	Formamidopyrimidine-DNA glycosylase N-terminal domain	1	2
pfam01152	Bacterial-like globin	2	2
pfam01155	Hydrogenase/urease nickel incorporation, metallochaperone, hypA	1	0
pfam01156	Inosine-uridine preferring nucleoside hydrolase	1	1
pfam01161	Phosphatidylethanolamine-binding protein	1	1
pfam01163	RIO1 family	1	0
pfam01165	Ribosomal protein S21	1	1
pfam01168	Alanine racemase, N-terminal domain	2	3
pfam01169	Uncharacterized protein family UPF0016	1	1
pfam01170	Putative RNA methylase family UPF0020	1	1
pfam01171	PP-loop family	2	2
pfam01175	Urocanase	1	1
pfam01176	Translation initiation factor 1A / IF-1	1	1
pfam01177	Asp/Glu/Hydantoin racemase	1	1
pfam01180	Dihydroorotate dehydrogenase	1	2
pfam01182	Glucosamine-6-phosphate isomerases/6-phosphogluconolactonase	1	0
pfam01184	GPR1/FUN34/yaaH family	1	0
pfam01189	16S rRNA methyltransferase RsmB/F	4	2

pfam01192	RNA polymerase Rpb6	1	1
pfam01193	RNA polymerase Rpb3/Rpb11 dimerisation domain	1	1
pfam01195	Peptidyl-tRNA hydrolase	1	1
pfam01196	Ribosomal protein L17	1	1
pfam01197	Ribosomal protein L31	1	1
pfam01202	Shikimate kinase	2	3
pfam01203	Type II secretion system (T2SS), protein N	1	1
pfam01205	Uncharacterized protein family UPF0029	1	1
pfam01206	Sulfurtransferase TusA	2	1
pfam01207	Dihydrouridine synthase (Dus)	3	2
pfam01208	Uroporphyrinogen decarboxylase (URO-D)	1	1
pfam01209	ubiE/COQ5 methyltransferase family	1	1
pfam01210	NAD-dependent glycerol-3-phosphate dehydrogenase N-terminus	1	1
pfam01212	Beta-eliminating lyase	1	2
pfam01218	Coproporphyrinogen III oxidase	1	1
pfam01219	Prokaryotic diacylglycerol kinase	1	1
pfam01220	Dehydroquinase class II	1	1
pfam01223	DNA/RNA non-specific endonuclease	0	1
pfam01225	Mur ligase family, catalytic domain	4	4
pfam01226	Formate/nitrite transporter	1	0
pfam01227	GTP cyclohydrolase I	1	2
pfam01228	Glycine radical	1	0
pfam01229	Glycosyl hydrolases family 39	0	1
pfam01230	HIT domain	1	2
pfam01232	Mannitol dehydrogenase Rossmann domain	0	1
pfam01235	Sodium:alanine symporter family	3	0
pfam01242	6-pyruvoyl tetrahydropterin synthase	1	1
pfam01243	Pyridoxamine 5'-phosphate oxidase	2	3
pfam01244	Membrane dipeptidase (Peptidase family M19)	1	2
pfam01245	Ribosomal protein L19	1	1
pfam01250	Ribosomal protein S6	1	1
pfam01252	Signal peptidase (SPase) II	1	1
pfam01253	Translation initiation factor SUI1	1	1
pfam01255	Putative undecaprenyl diphosphate synthase	1	1

pfam01256	Carbohydrate kinase	1	2
pfam01257	Thioredoxin-like [2Fe-2S] ferredoxin	0	2
pfam01258	Prokaryotic dksA/traR C4-type zinc finger	3	2
pfam01259	SAICAR synthetase	1	1
pfam01261	Xylose isomerase-like TIM barrel	0	1
pfam01262	Alanine dehydrogenase/PNT, C-terminal domain	2	2
pfam01263	Aldose 1-epimerase	2	3
pfam01264	Chorismate synthase	1	1
pfam01266	FAD dependent oxidoreductase	5	6
pfam01268	Formate--tetrahydrofolate ligase	1	0
pfam01272	Transcription elongation factor, GreA/GreB, C-term	4	2
pfam01274	Malate synthase	1	1
pfam01276	Orn/Lys/Arg decarboxylase, major domain	1	1
pfam01281	Ribosomal protein L9, N-terminal domain	1	1
pfam01288	7,8-dihydro-6-hydroxymethylpterin-pyrophosphokinase (HPPK)	2	1
pfam01292	Prokaryotic cytochrome b561	8	4
pfam01293	Phosphoenolpyruvate carboxykinase	1	0
pfam01295	Adenylate cyclase, class-I	1	0
pfam01297	Zinc-uptake complex component A periplasmic	1	0
pfam01300	Telomere recombination	3	2
pfam01301	Glycosyl hydrolases family 35	0	2
pfam01311	Bacterial export proteins, family 1	3	2
pfam01312	FlhB HrpN YscU SpaS Family	5	3
pfam01313	Bacterial export proteins, family 3	4	2
pfam01314	Aldehyde ferredoxin oxidoreductase, domains 2 & 3	1	0
pfam01315	Aldehyde oxidase and xanthine dehydrogenase, a/b hammerhead doma	0	5
pfam01316	Arginine repressor, DNA binding domain	1	0
pfam01321	Creatinase/Prolidase N-terminal domain	1	2
pfam01322	Cytochrome C'	1	1
pfam01323	DSBA-like thioredoxin domain	3	5
pfam01326	Pyruvate phosphate dikinase, PEP/pyruvate binding domain	1	1
pfam01327	Polypeptide deformylase	3	2
pfam01329	Pterin 4 alpha carbinolamine dehydratase	1	1
pfam01330	RuvA N terminal domain	1	1

pfam01336	OB-fold nucleic acid binding domain	3	3
pfam01337	Barstar (barnase inhibitor)	0	2
pfam01339	CheB methylesterase	2	5
pfam01340	Met Apo-repressor, MetJ	1	0
pfam01343	Peptidase family S49	2	3
pfam01346	Domain amino terminal to FKBP-type peptidyl-prolyl isomerase	3	0
pfam01351	Ribonuclease HII	1	1
pfam01361	Tautomerase enzyme	0	3
pfam01367	5'-3' exonuclease, C-terminal SAM fold	2	2
pfam01368	DHH family	2	1
pfam01370	NAD dependent epimerase/dehydratase family	2	11
pfam01371	Trp repressor protein	1	0
pfam01379	Porphobilinogen deaminase, dipyromethane cofactor binding domain	1	2
pfam01380	SIS domain	4	8
pfam01381	Helix-turn-helix	13	7
pfam01384	Phosphate transporter family	2	2
pfam01386	Ribosomal L25p family	1	1
pfam01389	OmpA-like transmembrane domain	1	0
pfam01391	Collagen triple helix repeat (20 copies)	2	0
pfam01396	Topoisomerase DNA binding C4 zinc finger	3	0
pfam01401	Angiotensin-converting enzyme	1	1
pfam01402	Ribbon-helix-helix protein, copG family	1	0
pfam01406	tRNA synthetases class I (C) catalytic domain	1	2
pfam01408	Oxidoreductase family, NAD-binding Rossmann fold	1	4
pfam01409	tRNA synthetases class II core domain (F)	1	1
pfam01411	tRNA synthetases class II (A)	1	1
pfam01416	tRNA pseudouridine synthase	1	1
pfam01418	Helix-turn-helix domain, rpiR family	1	3
pfam01420	Type I restriction modification DNA specificity domain	5	0
pfam01425	Amidase	1	3
pfam01430	Hsp33 protein	1	1
pfam01431	Peptidase family M13	3	4
pfam01432	Peptidase family M3	5	3
pfam01433	Peptidase family M1	5	8

pfam01434	Peptidase family M41	1	2
pfam01435	Peptidase family M48	4	9
pfam01436	NHL repeat	0	1
pfam01447	Thermolysin metallopeptidase, catalytic domain	1	2
pfam01450	Acetohydroxy acid isomeroreductase, catalytic domain	1	1
pfam01451	Low molecular weight phosphotyrosine protein phosphatase	1	3
pfam01455	HupF/HypC family	1	0
pfam01464	Transglycosylase SLT domain	6	4
pfam01467	Cytidylyltransferase-like	4	4
pfam01470	Pyroglutamyl peptidase	0	1
pfam01471	Putative peptidoglycan binding domain	2	3
pfam01472	PUA domain	1	1
pfam01475	Ferric uptake regulator family	1	6
pfam01476	LysM domain	6	6
pfam01478	Type IV leader peptidase family	1	3
pfam01479	S4 domain	8	8
pfam01483	Proprotein convertase P-domain	1	0
pfam01488	Shikimate / quinate 5-dehydrogenase	2	2
pfam01491	Frataxin-like domain	1	1
pfam01493	GXGXG motif	1	1
pfam01494	FAD binding domain	4	8
pfam01497	Periplasmic binding protein	2	2
pfam01502	Phosphoribosyl-AMP cyclohydrolase	1	1
pfam01503	Phosphoribosyl-ATP pyrophosphohydrolase	2	1
pfam01507	Phosphoadenosine phosphosulfate reductase family	2	2
pfam01509	TruB family pseudouridylate synthase (N terminal domain)	1	1
pfam01510	N-acetylmuramoyl-L-alanine amidase	1	2
pfam01512	Respiratory-chain NADH dehydrogenase 51 Kd subunit	1	1
pfam01513	ATP-NAD kinase	2	1
pfam01514	Secretory protein of YscJ/FliF family	3	2
pfam01515	Phosphate acetyl/butaryl transferase	1	2
pfam01520	N-acetylmuramoyl-L-alanine amidase	1	1
pfam01521	Iron-sulphur cluster biosynthesis	3	2
pfam01522	Polysaccharide deacetylase	2	8

pfam01523	Putative modulator of DNA gyrase	2	6
pfam01526	Tn3 transposase DDE domain	5	0
pfam01527	Transposase	50	1
pfam01536	Adenosylmethionine decarboxylase	1	0
pfam01541	GIY-YIG catalytic domain	2	1
pfam01544	CorA-like Mg2+ transporter protein	2	3
pfam01545	Cation efflux family	1	3
pfam01546	Peptidase family M20/M25/M40	4	13
pfam01547	Bacterial extracellular solute-binding protein	1	3
pfam01548	Transposase	0	1
pfam01551	Peptidase family M23	8	4
pfam01553	Acyltransferase	8	4
pfam01554	MatE	7	2
pfam01555	DNA methylase	1	2
pfam01556	DnaJ C terminal domain	1	2
pfam01557	Fumarylacetoacetate (FAA) hydrolase family	2	5
pfam01558	Pyruvate ferredoxin/flavodoxin oxidoreductase	0	2
pfam01564	Spermine/spermidine synthase	2	4
pfam01565	FAD binding domain	4	6
pfam01566	Natural resistance-associated macrophage protein	1	4
pfam01568	Molydopterin dinucleotide binding domain	7	6
pfam01569	PAP2 superfamily	4	3
pfam01571	Aminomethyltransferase folate-binding domain	2	2
pfam01575	MaoC like domain	1	2
pfam01578	Cytochrome C assembly protein	3	2
pfam01580	FtsK/SpoIIIE family	1	1
pfam01583	Adenylylsulphate kinase	1	0
pfam01584	CheW-like domain	9	16
pfam01588	Putative tRNA binding domain	2	2
pfam01590	GAF domain	6	11
pfam01592	NifU-like N terminal domain	1	1
pfam01593	Flavin containing amine oxidoreductase	2	5
pfam01594	AI-2E family transporter	3	7
pfam01595	Domain of unknown function DUF21	3	2

pfam01596	O-methyltransferase	0	1
pfam01597	Glycine cleavage H-protein	1	1
pfam01609	Transposase DDE domain	10	0
pfam01610	Transposase	0	1
pfam01612	3'-5' exonuclease	3	2
pfam01613	Flavin reductase like domain	3	4
pfam01614	Bacterial transcriptional regulator	0	3
pfam01618	MotA/TolQ/ExbB proton channel family	7	9
pfam01619	Proline dehydrogenase	1	1
pfam01624	MutS domain I	1	1
pfam01625	Peptide methionine sulfoxide reductase	2	3
pfam01627	Hpt domain	8	21
pfam01628	HrcA protein C terminal domain	0	1
pfam01632	Ribosomal protein L35	1	1
pfam01634	ATP phosphoribosyltransferase	1	1
pfam01636	Phosphotransferase enzyme family	4	5
pfam01638	HxlR-like helix-turn-helix	2	6
pfam01641	SelR domain	1	2
pfam01642	Methylmalonyl-CoA mutase	0	1
pfam01645	Conserved region in glutamate synthase	3	2
pfam01648	4'-phosphopantetheinyl transferase superfamily	2	2
pfam01649	Ribosomal protein S20	1	1
pfam01653	NAD-dependent DNA ligase adenylation domain	2	1
pfam01654	Cytochrome bd terminal oxidase subunit I	2	1
pfam01656	CobQ/CobB/MinD/ParA nucleotide binding domain	4	4
pfam01663	Type I phosphodiesterase / nucleotide pyrophosphatase	0	2
pfam01668	SmpB protein	1	1
pfam01676	Metalloenzyme superfamily	2	1
pfam01678	Diaminopimelate epimerase	1	2
pfam01679	Proteolipid membrane potential modulator	1	0
pfam01687	Riboflavin kinase	1	1
pfam01694	Rhomboid family	3	1
pfam01695	IstB-like ATP binding protein	39	0
pfam01699	Sodium/calcium exchanger protein	2	1

pfam01702	Queuine tRNA-ribosyltransferase	1	1
pfam01706	FliG C-terminal domain	2	2
pfam01709	Transcriptional regulator	2	1
pfam01713	Smr domain	2	1
pfam01715	IPP transferase	1	1
pfam01717	Cobalamin-independent synthase, Catalytic domain	2	1
pfam01722	BolA-like protein	2	2
pfam01724	Domain of unknown function DUF29	0	1
pfam01725	Ham1 family	1	1
pfam01726	LexA DNA binding domain	1	1
pfam01728	FtsJ-like methyltransferase	2	1
pfam01729	Quinolinate phosphoribosyl transferase, C-terminal domain	1	1
pfam01730	UreF	0	1
pfam01734	Patatin-like phospholipase	5	6
pfam01738	Dienelactone hydrolase family	4	5
pfam01739	CheR methyltransferase, SAM binding domain	2	7
pfam01740	STAS domain	4	4
pfam01741	Large-conductance mechanosensitive channel, MscL	1	1
pfam01743	Poly A polymerase head domain	2	2
pfam01746	tRNA (Guanine-1)-methyltransferase	1	1
pfam01750	Hydrogenase maturation protease	1	0
pfam01751	Toprim domain	6	3
pfam01752	Collagenase	1	0
pfam01757	Acyltransferase family	1	11
pfam01758	Sodium Bile acid symporter family	1	0
pfam01761	3-dehydroquinate synthase	1	1
pfam01764	Lipase (class 3)	1	0
pfam01765	Ribosome recycling factor	1	1
pfam01769	Divalent cation transporter	3	0
pfam01773	Na+ dependent nucleoside transporter N-terminus	2	0
pfam01774	UreD urease accessory protein	0	1
pfam01782	RimM N-terminal domain	1	1
pfam01783	Ribosomal L32p protein family	1	1
pfam01784	NIF3 (NGG1p interacting factor 3)	1	0

pfam01790	Prolipoprotein diacylglyceryl transferase	2	2
pfam01791	DeoC/LacD family aldolase	1	1
pfam01794	Ferric reductase like transmembrane component	1	1
pfam01795	MraW methylase family	1	1
pfam01797	Transposase IS200 like	0	2
pfam01799	[2Fe-2S] binding domain	2	11
pfam01804	Penicillin amidase	1	2
pfam01807	CHC2 zinc finger	1	1
pfam01808	AICARFT/IMPCHase bienzyme	1	1
pfam01809	Haemolytic domain	1	2
pfam01810	LysE type translocator	9	9
pfam01812	5-formyltetrahydrofolate cyclo-ligase family	1	1
pfam01814	Hemerythrin HHE cation binding domain	3	3
pfam01817	Chorismate mutase type II	3	1
pfam01820	D-ala D-ala ligase N-terminus	1	1
pfam01832	Mannosyl-glycoprotein endo-beta-N-acetylglucosaminidase	2	3
pfam01835	MG2 domain	1	1
pfam01841	Transglutaminase-like superfamily	2	4
pfam01842	ACT domain	3	3
pfam01844	HNH endonuclease	1	1
pfam01848	Hok/gef family	1	0
pfam01850	PIN domain	0	3
pfam01863	Protein of unknown function DUF45	1	2
pfam01865	Protein of unknown function DUF47	1	1
pfam01869	BadF/BadG/BcrA/BcrD ATPase family	1	3
pfam01872	RibD C-terminal domain	2	1
pfam01874	ATP:dephospho-CoA triphosphoribosyl transferase	0	1
pfam01878	EVE domain	1	1
pfam01882	Protein of unknown function DUF58	2	2
pfam01883	Domain of unknown function DUF59	0	2
pfam01885	RNA 2'-phosphotransferase, Tpt1 / KptA family	0	1
pfam01887	S-adenosyl-l-methionine hydroxide adenosyltransferase	0	1
pfam01888	CbiD	0	1
pfam01890	Cobalamin synthesis G C-terminus	0	1

pfam01891	Cobalt uptake substrate-specific transmembrane region	1	1
pfam01895	PhoU domain	1	1
pfam01903	CbiX	0	2
pfam01904	Protein of unknown function DUF72	1	2
pfam01909	Nucleotidyltransferase domain	1	1
pfam01914	MarC family integral membrane protein	2	1
pfam01915	Glycosyl hydrolase family 3 C-terminal domain	1	6
pfam01923	Cobalamin adenosyltransferase	0	1
pfam01924	Hydrogenase formation hypA family	1	0
pfam01925	Sulfite exporter TauE/SafE	8	3
pfam01926	50S ribosome-binding GTPase	8	9
pfam01928	CYTH domain	2	2
pfam01931	Protein of unknown function DUF84	1	0
pfam01933	Uncharacterised protein family UPF0052	1	0
pfam01936	NYN domain	1	1
pfam01938	TRAM domain	2	1
pfam01943	Polysaccharide biosynthesis protein	1	0
pfam01957	NfeD-like C-terminal, partner-binding	1	1
pfam01960	ArgJ family	0	1
pfam01963	TraB family	1	3
pfam01964	Radical SAM ThiC family	1	1
pfam01965	DJ-1/PfpI family	4	18
pfam01966	HD domain	7	9
pfam01967	MoaC family	1	1
pfam01968	Hydantoinase/oxoprolinase	0	1
pfam01970	Tripartite tricarboxylate transporter TctA family	1	0
pfam01973	Protein of unknown function DUF115	2	0
pfam01975	Survival protein SurE	1	2
pfam01977	3-octaprenyl-4-hydroxybenzoate carboxy-lyase	1	1
pfam01979	Amidohydrolase family	5	16
pfam01980	Uncharacterised protein family UPF0066	1	0
pfam01985	CRS1 / YhbY (CRM) domain	1	1
pfam01987	Mitochondrial biogenesis AIM24	1	1
pfam02001	Protein of unknown function DUF134	1	0

pfam02016	LD-carboxypeptidase	0	1
pfam02021	Uncharacterised protein family UPF0102	1	1
pfam02028	BCCT, betaine/carnitine/choline family transporter	1	0
pfam02033	Ribosome-binding factor A	1	1
pfam02036	SCP-2 sterol transfer family	2	2
pfam02040	Arsenical pump membrane protein	0	2
pfam02049	Flagellar hook-basal body complex protein FliE	2	2
pfam02050	Flagellar FliJ protein	1	2
pfam02055	Glycosyl hydrolase family 30 TIM-barrel domain	0	1
pfam02065	Melibiase	0	1
pfam02074	Carboxypeptidase Taq (M32) metallopeptidase	1	0
pfam02075	Crossover junction endodeoxyribonuclease RuvC	1	1
pfam02080	TrkA-C domain	4	2
pfam02082	Transcriptional regulator	2	3
pfam02085	Class III cytochrome C family	1	0
pfam02086	D12 class N6 adenine-specific DNA methyltransferase	1	1
pfam02091	Glycyl-tRNA synthetase alpha subunit	1	1
pfam02092	Glycyl-tRNA synthetase beta subunit	1	1
pfam02096	60Kd inner membrane protein	1	1
pfam02104	SURF1 family	1	2
pfam02107	Flagellar L-ring protein	2	2
pfam02108	Flagellar assembly protein FliH	2	2
pfam02113	D-Ala-D-Ala carboxypeptidase 3 (S13) family	1	1
pfam02119	Flagellar P-ring protein	2	2
pfam02120	Flagellar hook-length control protein FliK	2	3
pfam02129	X-Pro dipeptidyl-peptidase (S15 family)	0	5
pfam02130	Uncharacterized protein family UPF0054	1	1
pfam02132	RecR protein	1	1
pfam02133	Permease for cytosine/purines, uracil, thiamine, allantoin	0	1
pfam02142	MGS-like domain	2	3
pfam02146	Sir2 family	1	2
pfam02148	Zn-finger in ubiquitin-hydrolases and other protein	0	1
pfam02151	UvrB/uvrC motif	2	2
pfam02152	Dihydroneopterin aldolase	2	1

pfam02153	Prephenate dehydrogenase	1	1
pfam02154	Flagellar motor switch protein FliM	1	1
pfam02156	Glycosyl hydrolase family 26	0	1
pfam02163	Peptidase family M50	1	2
pfam02167	Cytochrome C1 family	1	1
pfam02169	LPP20 lipoprotein	2	0
pfam02190	ATP-dependent protease La (LON) substrate-binding domain	3	2
pfam02195	ParB-like nuclease domain	2	2
pfam02201	SWIB/MDM2 domain	0	1
pfam02203	Tar ligand binding domain homologue	0	5
pfam02205	WH2 motif	1	0
pfam02219	Methylenetetrahydrofolate reductase	1	1
pfam02222	ATP-grasp domain	2	2
pfam02223	Thymidylate kinase	1	1
pfam02224	Cytidylate kinase	1	1
pfam02225	PA domain	6	5
pfam02230	Phospholipase/Carboxylesterase	1	2
pfam02233	NAD(P) transhydrogenase beta subunit	1	1
pfam02237	Biotin protein ligase C terminal domain	1	1
pfam02245	Methylpurine-DNA glycosylase (MPG)	0	1
pfam02253	Phospholipase A1	1	1
pfam02254	TrkA-N domain	5	3
pfam02261	Aspartate decarboxylase	0	1
pfam02265	S1/P1 Nuclease	0	1
pfam02272	DHHA1 domain	2	2
pfam02277	Phosphoribosyltransferase	1	1
pfam02281	Transposase Tn5 dimerisation domain	1	0
pfam02283	Cobinamide kinase / cobinamide phosphate guanyltransferase	1	1
pfam02303	Helix-destabilising protein	1	0
pfam02308	MgtC family	0	2
pfam02310	B12 binding domain	1	4
pfam02311	AraC-like ligand binding domain	6	6
pfam02321	Outer membrane efflux protein	5	19
pfam02322	Cytochrome bd terminal oxidase subunit II	2	1

pfam02325	YGGT family	1	1
pfam02333	Phytase	1	0
pfam02335	Cytochrome c552	1	0
pfam02342	TerD domain	0	2
pfam02347	Glycine cleavage system P-protein	1	1
pfam02348	Cytidylyltransferase	2	1
pfam02350	UDP-N-acetylglucosamine 2-epimerase	1	0
pfam02353	Mycolic acid cyclopropane synthetase	1	2
pfam02355	Protein export membrane protein	4	2
pfam02357	Transcription termination factor nusG	2	1
pfam02361	Cobalt transport protein	1	1
pfam02367	Threonylcarbamoyl adenosine biosynthesis protein TsaE	1	1
pfam02368	Bacterial Ig-like domain (group 2)	1	0
pfam02369	Bacterial Ig-like domain (group 1)	1	1
pfam02371	Transposase IS116/IS110/IS902 family	0	1
pfam02378	Phosphotransferase system, EIIC	1	0
pfam02381	MraZ protein, putative antitoxin-like	1	1
pfam02384	N-6 DNA Methylase	5	0
pfam02386	Cation transport protein	3	0
pfam02390	Putative methyltransferase	1	1
pfam02391	MoaE protein	1	1
pfam02397	Bacterial sugar transferase	1	1
pfam02399	Origin of replication binding protein	0	1
pfam02401	LytB protein	1	1
pfam02403	Seryl-tRNA synthetase N-terminal domain	1	1
pfam02405	Permease MlaE	1	3
pfam02410	Ribosomal silencing factor during starvation	1	1
pfam02416	mttA/Hcf106 family	2	2
pfam02417	Chromate transporter	1	3
pfam02421	Ferrous iron transport protein B	1	1
pfam02423	Ornithine cyclodeaminase/mu-crystallin family	0	1
pfam02424	ApbE family	3	2
pfam02433	Cytochrome C oxidase, mono-heme subunit/FixO	1	1
pfam02436	Conserved carboxylase domain	1	0

pfam02441	Flavoprotein	2	2
pfam02445	Quinolinate synthetase A protein	1	1
pfam02446	4-alpha-glucanotransferase	1	0
pfam02447	GntP family permease	2	1
pfam02449	Beta-galactosidase	0	2
pfam02452	PemK-like, MazF-like toxin of type II toxin-antitoxin system	1	0
pfam02457	DisA bacterial checkpoint controller nucleotide-binding	1	0
pfam02463	RecF/RecN/SMC N terminal domain	4	2
pfam02464	Competence-damaged protein	1	1
pfam02465	Flagellar hook-associated protein 2 N-terminus	2	3
pfam02470	MlaD protein	2	4
pfam02472	Biopolymer transport protein ExbD/TolR	5	6
pfam02481	DNA recombination-mediator protein A	2	1
pfam02482	Sigma 54 modulation protein / S30EA ribosomal protein	3	2
pfam02485	Core-2/I-Branching enzyme	0	1
pfam02491	SHS2 domain inserted in FTSA	1	1
pfam02492	CobW/HypB/UreG, nucleotide-binding domain	2	3
pfam02501	Type II secretion system (T2SS), protein I	1	1
pfam02503	Polyphosphate kinase middle domain	1	1
pfam02504	Fatty acid synthesis protein	1	1
pfam02508	Rnf-Nqr subunit, membrane protein	6	0
pfam02515	CoA-transferase family III	0	3
pfam02517	CAAX protease self-immunity	1	9
pfam02518	Histidine kinase-, DNA gyrase B-, and HSP90-like ATPase	45	130
pfam02525	Flavodoxin-like fold	2	4
pfam02527	rRNA small subunit methyltransferase G	1	1
pfam02535	ZIP Zinc transporter	0	1
pfam02537	CrcB-like protein, Camphor Resistance (CrcB)	1	0
pfam02538	Hydantoinase B/oxoprolinase	0	1
pfam02540	NAD synthase	2	2
pfam02541	Ppx/GppA phosphatase family	2	1
pfam02542	YgbB family	1	1
pfam02545	Maf-like protein	2	2
pfam02547	Queuosine biosynthesis protein	1	1

pfam02548	Ketopantoate hydroxymethyltransferase	2	1
pfam02550	Acetyl-CoA hydrolase/transferase N-terminal domain	1	1
pfam02553	Cobalt transport protein component CbiN	0	1
pfam02554	Carbon starvation protein CstA	2	1
pfam02556	Preprotein translocase subunit SecB	1	1
pfam02557	D-alanyl-D-alanine carboxypeptidase	1	0
pfam02558	Ketopantoate reductase PanE/ApbA	1	1
pfam02559	CarD-like/TRCF domain	1	1
pfam02560	Cyanate lyase C-terminal domain	0	1
pfam02561	Flagellar protein FliS	2	2
pfam02562	PhoH-like protein	2	2
pfam02563	Polysaccharide biosynthesis/export protein	1	1
pfam02565	Recombination protein O C terminal	1	1
pfam02566	OsmC-like protein	3	7
pfam02567	Phenazine biosynthesis-like protein	4	3
pfam02568	Thiamine biosynthesis protein (ThiI)	1	0
pfam02569	Pantoate-beta-alanine ligase	1	1
pfam02570	Precorrin-8X methylmutase	0	1
pfam02572	ATP:corrinoid adenosyltransferase BtuR/CobO/CobP	1	1
pfam02574	Homocysteine S-methyltransferase	2	1
pfam02575	YbaB/EbfC DNA-binding family	1	1
pfam02576	Putative ribosome maturation factor RimP	1	1
pfam02578	Multi-copper polyphenol oxidoreductase laccase	1	1
pfam02579	Dinitrogenase iron-molybdenum cofactor	1	0
pfam02580	D-Tyr-tRNA(Tyr) deacylase	1	1
pfam02581	Thiamine monophosphate synthase/TENI	1	1
pfam02585	GlcNAc-PI de-N-acetylase	0	1
pfam02586	SOS response associated peptidase (SRAP)	0	1
pfam02589	LUD domain	2	0
pfam02590	Predicted SPOUT methyltransferase	1	1
pfam02592	Putative vitamin uptake transporter	1	0
pfam02594	Uncharacterised ACR, YggU family COG1872	1	0
pfam02595	Glycerate kinase family	1	1
pfam02597	ThiS family	2	2

pfam02599	Global regulator protein family	1	0
pfam02600	Disulfide bond formation protein DsbB	1	1
pfam02601	Exonuclease VII, large subunit	1	1
pfam02602	Uroporphyrinogen-III synthase HemD	1	1
pfam02603	HPr Serine kinase N terminus	0	1
pfam02604	Antitoxin Phd_YefM, type II toxin-antitoxin system	2	4
pfam02606	Tetraacyldisaccharide-1-P 4'-kinase	1	1
pfam02607	B12 binding domain	1	1
pfam02608	ABC transporter substrate-binding protein PnrA-like	0	2
pfam02609	Exonuclease VII small subunit	1	1
pfam02613	Nitrate reductase delta subunit	2	0
pfam02616	Segregation and condensation protein ScpA	1	1
pfam02617	ATP-dependent Clp protease adaptor protein ClpS	1	1
pfam02618	YceG-like family	1	1
pfam02620	Uncharacterized ACR, COG1399	1	1
pfam02622	Uncharacterized ACR, COG1678	1	1
pfam02624	YcaO cyclodehydratase, ATP-ad Mg2+-binding	1	1
pfam02625	XdhC and CoxI family	1	2
pfam02626	Carboxyltransferase domain, subdomain A and B	1	0
pfam02627	Carboxymuconolactone decarboxylase family	3	10
pfam02628	Cytochrome oxidase assembly protein	1	1
pfam02629	CoA binding domain	1	1
pfam02630	SCO1/SenC	1	2
pfam02631	RecX family	2	1
pfam02633	Creatinine amidohydrolase	0	1
pfam02634	FdhD/NarQ family	1	1
pfam02635	DsrE/DsrF-like family	4	0
pfam02636	Putative S-adenosyl-L-methionine-dependent methyltransferase	0	1
pfam02637	GatB domain	0	1
pfam02638	Glycosyl hydrolase-like 10	0	1
pfam02639	Uncharacterized BCR, YaiI/YqxD family COG1671	1	1
pfam02643	Uncharacterized ACR, COG1430	0	1
pfam02646	RmuC family	1	1
pfam02652	L-lactate permease	1	0

pfam02653	Branched-chain amino acid transport system / permease component	0	21
pfam02654	Cobalamin-5-phosphate synthase	1	1
pfam02655	ATP-grasp domain	1	0
pfam02657	Fe-S metabolism associated domain	1	0
pfam02659	Putative manganese efflux pump	0	1
pfam02660	Glycerol-3-phosphate acyltransferase	1	1
pfam02661	Fic/DOC family	4	0
pfam02664	S-Ribosylhomocysteinase (LuxS)	1	0
pfam02666	Phosphatidylserine decarboxylase	1	1
pfam02667	Short chain fatty acid transporter	1	0
pfam02668	Taurine catabolism dioxygenase TauD, TfdA family	0	3
pfam02669	K+-transporting ATPase, c chain	0	1
pfam02670	1-deoxy-D-xylulose 5-phosphate reductoisomerase	1	1
pfam02673	Bacitracin resistance protein BacA	1	1
pfam02674	Colicin V production protein	1	1
pfam02675	S-adenosylmethionine decarboxylase	0	1
pfam02678	Pirin	3	7
pfam02681	Divergent PAP2 family	0	1
pfam02683	Cytochrome C biogenesis protein transmembrane region	2	3
pfam02684	Lipid-A-disaccharide synthetase	1	1
pfam02685	Glucokinase	0	3
pfam02686	Glu-tRNAGln amidotransferase C subunit	0	1
pfam02687	FtsX-like permease family	8	15
pfam02694	Uncharacterised BCR, YnfA/UPF0060 family	0	1
pfam02696	Uncharacterized ACR, YdiU/UPF0061 family	1	1
pfam02698	DUF218 domain	1	0
pfam02699	Preprotein translocase subunit	1	1
pfam02702	Osmosensitive K+ channel His kinase sensor domain	0	1
pfam02705	K+ potassium transporter	0	1
pfam02706	Chain length determinant protein	1	1
pfam02709	N-terminal domain of galactosyltransferase	0	1
pfam02719	Polysaccharide biosynthesis protein	2	0
pfam02729	Aspartate/ornithine carbamoyltransferase, carbamoyl-P binding domain	2	2
pfam02730	Aldehyde ferredoxin oxidoreductase, N-terminal domain	1	0

pfam02732	ERCC4 domain	1	0
pfam02735	Ku70/Ku80 beta-barrel domain	0	1
pfam02737	3-hydroxyacyl-CoA dehydrogenase, NAD binding domain	2	3
pfam02738	Molybdopterin-binding domain of aldehyde dehydrogenase	2	12
pfam02739	5'-3' exonuclease, N-terminal resolvase-like domain	2	2
pfam02743	Cache domain	15	8
pfam02746	Mandelate racemase / muconate lactonizing enzyme, N-terminal domai	0	2
pfam02749	Quinolinate phosphoribosyl transferase, N-terminal domain	1	1
pfam02754	Cysteine-rich domain	2	2
pfam02767	DNA polymerase III beta subunit, central domain	1	1
pfam02768	DNA polymerase III beta subunit, C-terminal domain	1	1
pfam02769	AIR synthase related protein, C-terminal domain	5	4
pfam02770	Acyl-CoA dehydrogenase, middle domain	5	13
pfam02771	Acyl-CoA dehydrogenase, N-terminal domain	6	13
pfam02772	S-adenosylmethionine synthetase, central domain	1	1
pfam02773	S-adenosylmethionine synthetase, C-terminal domain	1	1
pfam02774	Semialdehyde dehydrogenase, dimerisation domain	2	2
pfam02775	Thiamine pyrophosphate enzyme, C-terminal TPP binding domain	4	6
pfam02776	Thiamine pyrophosphate enzyme, N-terminal TPP binding domain	4	4
pfam02777	Iron/manganese superoxide dismutases, C-terminal domain	1	1
pfam02779	Transketolase, pyrimidine binding domain	5	4
pfam02780	Transketolase, C-terminal domain	4	3
pfam02781	Glucose-6-phosphate dehydrogenase, C-terminal domain	1	1
pfam02782	FGGY family of carbohydrate kinases, C-terminal domain	1	2
pfam02784	Pyridoxal-dependent decarboxylase, pyridoxal binding domain	3	1
pfam02785	Biotin carboxylase C-terminal domain	2	2
pfam02786	Carbamoyl-phosphate synthase L chain, ATP binding domain	3	5
pfam02787	Carbamoyl-phosphate synthetase large chain, oligomerisation domain	1	1
pfam02789	Cytosol aminopeptidase family, N-terminal domain	2	2
pfam02790	Cytochrome C oxidase subunit II, transmembrane domain	1	1
pfam02796	Helix-turn-helix domain of resolvase	4	0
pfam02798	Glutathione S-transferase, N-terminal domain	4	11
pfam02800	Glyceraldehyde 3-phosphate dehydrogenase, C-terminal domain	4	1
pfam02801	Beta-ketoacyl synthase, C-terminal domain	6	2

pfam02803	Thiolase, C-terminal domain	3	7
pfam02805	Metal binding domain of Ada	1	1
pfam02806	Alpha amylase, C-terminal all-beta domain	1	0
pfam02810	SEC-C motif	3	4
pfam02811	PHP domain	3	4
pfam02812	Glu/Leu/Phe/Val dehydrogenase, dimerisation domain	1	1
pfam02814	UreE urease accessory protein, N-terminal domain	0	1
pfam02817	e3 binding domain	3	2
pfam02823	ATP synthase, Delta/Epsilon chain, beta-sandwich domain	1	1
pfam02824	TGS domain	3	3
pfam02826	D-isomer specific 2-hydroxyacid dehydrogenase, NAD binding domain	5	7
pfam02831	gpW	0	1
pfam02833	DHHA2 domain	1	0
pfam02834	LigT like Phosphoesterase	1	0
pfam02836	Glycosyl hydrolases family 2, TIM barrel domain	0	3
pfam02837	Glycosyl hydrolases family 2, sugar binding domain	0	3
pfam02838	Glycosyl hydrolase family 20, domain 2	2	1
pfam02839	Carbohydrate binding domain	2	0
pfam02843	Phosphoribosylglycinamide synthetase, C domain	1	1
pfam02844	Phosphoribosylglycinamide synthetase, N domain	1	1
pfam02852	Pyridine nucleotide-disulphide oxidoreductase, dimerisation domain	2	3
pfam02861	Clp amino terminal domain, pathogenicity island component	2	4
pfam02863	Arginine repressor, C-terminal domain	1	0
pfam02866	lactate/malate dehydrogenase, alpha/beta C-terminal domain	1	1
pfam02867	Ribonucleotide reductase, barrel domain	1	1
pfam02868	Thermolysin metallopeptidase, alpha-helical domain	1	2
pfam02872	5'-nucleotidase, C-terminal domain	3	3
pfam02873	UDP-N-acetylenolpyruvoylglucosamine reductase, C-terminal domain	1	1
pfam02874	ATP synthase alpha/beta family, beta-barrel domain	3	3
pfam02875	Mur ligase family, glutamate ligase domain	6	9
pfam02878	Phosphoglucomutase/phosphomannomutase, alpha/beta/alpha domair	4	3
pfam02879	Phosphoglucomutase/phosphomannomutase, alpha/beta/alpha domair	4	3
pfam02880	Phosphoglucomutase/phosphomannomutase, alpha/beta/alpha domair	4	3
pfam02881	SRP54-type protein, helical bundle domain	2	2

pfam02882	Tetrahydrofolate dehydrogenase/cyclohydrolase, NAD(P)-binding domai	1	1
pfam02885	Glycosyl transferase family, helical bundle domain	2	3
pfam02887	Pyruvate kinase, alpha/beta domain	1	2
pfam02894	Oxidoreductase family, C-terminal alpha/beta domain	0	1
pfam02895	Signal transducing histidine kinase, homodimeric domain	1	4
pfam02896	PEP-utilising enzyme, TIM barrel domain	3	2
pfam02897	Prolyl oligopeptidase, N-terminal beta-propeller domain	3	3
pfam02899	Phage integrase, N-terminal SAM-like domain	2	2
pfam02900	Catalytic LigB subunit of aromatic ring-opening dioxygenase	1	3
pfam02901	Pyruvate formate lyase-like	1	0
pfam02909	Tetracyclin repressor, C-terminal all-alpha domain	0	2
pfam02910	Fumarate reductase flavoprotein C-term	4	2
pfam02911	Formyl transferase, C-terminal domain	1	2
pfam02912	Aminoacyl tRNA synthetase class II, N-terminal domain	1	1
pfam02913	FAD linked oxidases, C-terminal domain	2	4
pfam02922	Carbohydrate-binding module 48 (Isoamylase N-terminal domain)	2	2
pfam02924	Bacteriophage lambda head decoration protein D	0	1
pfam02926	THUMP domain	2	1
pfam02927	Cellulase N-terminal ig-like domain	0	1
pfam02934	GatB/GatE catalytic domain	0	1
pfam02938	GAD domain	1	1
pfam02951	Prokaryotic glutathione synthetase, N-terminal domain	1	1
pfam02954	Bacterial regulatory protein, Fis family	14	11
pfam02955	Prokaryotic glutathione synthetase, ATP-grasp domain	1	2
pfam02962	5-carboxymethyl-2-hydroxymuconate isomerase	0	1
pfam02965	Vitamin B12 dependent methionine synthase, activation domain	1	1
pfam02976	DNA mismatch repair enzyme MutH	1	0
pfam02978	Signal peptide binding domain	1	1
pfam03006	Haemolysin-III related	1	1
pfam03007	Wax ester synthase-like Acyl-CoA acyltransferase domain	0	1
pfam03009	Glycerophosphoryl diester phosphodiesterase family	3	2
pfam03015	Male sterility protein	0	1
pfam03022	Major royal jelly protein	1	0
pfam03023	MviN-like protein	1	1

pfam03030	Inorganic H+ pyrophosphatase	0	1
pfam03033	Glycosyltransferase family 28 N-terminal domain	1	2
pfam03050	Transposase IS66 family	6	0
pfam03054	tRNA methyl transferase	1	1
pfam03055	Retinal pigment epithelial membrane protein	1	1
pfam03060	Nitronate monooxygenase	2	3
pfam03061	Thioesterase superfamily	10	6
pfam03063	Prismane/CO dehydrogenase family	1	0
pfam03065	Glycosyl hydrolase family 57	1	0
pfam03067	Lytic polysaccharide mono-oxygenase, cellulose-degrading	1	0
pfam03069	Acetamidase/Formamidase family	1	0
pfam03073	TspO/MBR family	0	1
pfam03091	CutA1 divalent ion tolerance protein	1	0
pfam03092	BT1 family	0	1
pfam03099	Biotin/lipoate A/B protein ligase family	2	2
pfam03100	CcmE	1	0
pfam03102	NeuB family	1	0
pfam03104	DNA polymerase family B, exonuclease domain	1	1
pfam03109	ABC1 family	1	1
pfam03116	**NQR2, RnfD, RnfE family**	**3**	**0**
pfam03118	Bacterial RNA polymerase, alpha chain C terminal domain	1	1
pfam03119	NAD-dependent DNA ligase C4 zinc finger domain	0	1
pfam03120	NAD-dependent DNA ligase OB-fold domain	1	1
pfam03129	Anticodon binding domain	4	3
pfam03143	Elongation factor Tu C-terminal domain	2	2
pfam03144	Elongation factor Tu domain 2	7	7
pfam03147	Ferredoxin-fold anticodon binding domain	1	1
pfam03150	Di-haem cytochrome c peroxidase	1	1
pfam03167	Uracil DNA glycosylase superfamily	2	4
pfam03169	OPT oligopeptide transporter protein	0	1
pfam03171	2OG-Fe(II) oxygenase superfamily	2	0
pfam03173	Putative carbohydrate binding domain	2	1
pfam03174	Chitobiase/beta-hexosaminidase C-terminal domain	1	1
pfam03176	MMPL family	2	1

pfam03180	NLPA lipoprotein	0	1
pfam03186	CobD/Cbib protein	1	2
pfam03193	Protein of unknown function, DUF258	1	1
pfam03205	Molybdopterin guanine dinucleotide synthesis protein B	1	1
pfam03222	Tryptophan/tyrosine permease family	4	0
pfam03232	Ubiquinone biosynthesis protein COQ7	0	1
pfam03235	Protein of unknown function DUF262	1	2
pfam03237	Terminase-like family	1	0
pfam03239	Iron permease FTR1 family	0	1
pfam03255	Acetyl co-enzyme A carboxylase carboxyltransferase alpha subunit	0	1
pfam03264	**NapC/NirT cytochrome c family, N-terminal region**	**3**	**0**
pfam03279	Bacterial lipid A biosynthesis acyltransferase	3	2
pfam03280	Proteobacterial lipase chaperone protein	0	1
pfam03301	Tryptophan 2,3-dioxygenase	0	1
pfam03308	ArgK protein	0	1
pfam03309	Type III pantothenate kinase	0	1
pfam03313	Serine dehydratase alpha chain	2	1
pfam03315	Serine dehydratase beta chain	1	1
pfam03328	HpcH/HpaI aldolase/citrate lyase family	0	3
pfam03330	Lytic transglycolase	2	1
pfam03331	UDP-3-O-acyl N-acetylglycosamine deacetylase	1	1
pfam03349	Outer membrane protein transport protein (OMPP1/FadL/TodX)	2	2
pfam03350	Uncharacterized protein family, UPF0114	1	1
pfam03352	Methyladenine glycosylase	2	1
pfam03358	NADPH-dependent FMN reductase	1	6
pfam03364	Polyketide cyclase / dehydrase and lipid transport	1	2
pfam03372	Endonuclease/Exonuclease/phosphatase family	2	6
pfam03379	CcmB protein	1	0
pfam03401	Tripartite tricarboxylate transporter family receptor	1	0
pfam03403	Platelet-activating factor acetylhydrolase, isoform II	0	1
pfam03404	Mo-co oxidoreductase dimerisation domain	1	0
pfam03411	Penicillin-insensitive murein endopeptidase	0	1
pfam03412	Peptidase C39 family	1	3
pfam03413	Peptidase propeptide and YPEB domain	2	1

pfam03433	EspA-like secreted protein	2	0
pfam03441	FAD binding domain of DNA photolyase	1	2
pfam03444	Winged helix-turn-helix transcription repressor, HrcA DNA-binding	0	1
pfam03445	Putative nucleotidyltransferase DUF294	1	0
pfam03446	NAD binding domain of 6-phosphogluconate dehydrogenase	3	3
pfam03447	Homoserine dehydrogenase, NAD binding domain	2	1
pfam03448	MgtE intracellular N domain	3	0
pfam03449	Transcription elongation factor, N-terminal	3	2
pfam03450	CO dehydrogenase flavoprotein C-terminal domain	0	5
pfam03453	MoeA N-terminal region (domain I and II)	2	2
pfam03454	MoeA C-terminal region (domain IV)	2	2
pfam03458	UPF0126 domain	2	2
pfam03459	TOBE domain	3	2
pfam03460	Nitrite/Sulfite reductase ferredoxin-like half domain	1	2
pfam03461	TRCF domain	1	1
pfam03462	PCRF domain	2	2
pfam03466	LysR substrate binding domain	52	106
pfam03471	Transporter associated domain	4	3
pfam03473	MOSC domain	2	1
pfam03475	3-alpha domain	1	0
pfam03476	MOSC N-terminal beta barrel domain	0	1
pfam03477	ATP cone domain	3	2
pfam03480	Bacterial extracellular solute-binding protein, family 7	2	2
pfam03481	Putative GTP-binding controlling metal-binding	0	1
pfam03483	B3/4 domain	1	1
pfam03484	tRNA synthetase B5 domain	1	1
pfam03485	Arginyl tRNA synthetase N terminal domain	1	1
pfam03486	HI0933-like protein	1	2
pfam03502	Nucleoside-specific channel-forming protein, Tsx	2	0
pfam03527	RHS protein	0	1
pfam03543	Yersinia/Haemophilus virulence surface antigen	1	0
pfam03544	Gram-negative bacterial TonB protein C-terminal	6	11
pfam03547	Membrane transport protein	2	2
pfam03548	Outer membrane lipoprotein carrier protein LolA	1	1

pfam03550	Outer membrane lipoprotein LolB	1	1
pfam03551	Transcriptional regulator PadR-like family	0	2
pfam03553	Na+/H+ antiporter family	5	0
pfam03562	MltA specific insert domain	0	1
pfam03571	Peptidase family M49	0	1
pfam03572	Peptidase family S41	4	10
pfam03575	Peptidase family S51	1	4
pfam03588	Leucyl/phenylalanyl-tRNA protein transferase	1	1
pfam03591	AzlC protein	2	1
pfam03594	Benzoate membrane transport protein	1	0
pfam03595	Voltage-dependent anion channel	1	3
pfam03597	Cytochrome oxidase maturation protein cbb3-type	1	1
pfam03600	Citrate transporter	3	0
pfam03601	Conserved hypothetical protein 698	1	1
pfam03602	Conserved hypothetical protein 95	1	1
pfam03605	Anaerobic c4-dicarboxylate membrane transporter	2	0
pfam03606	C4-dicarboxylate anaerobic carrier	1	1
pfam03610	PTS system fructose IIA component	0	1
pfam03616	Sodium/glutamate symporter	1	0
pfam03618	Kinase/pyrophosphorylase	1	1
pfam03625	Domain of unknown function DUF302	0	1
pfam03626	Prokaryotic Cytochrome C oxidase subunit IV	1	1
pfam03631	Virulence factor BrkB	1	3
pfam03632	Glycosyl hydrolase family 65 central catalytic domain	0	1
pfam03633	Glycosyl hydrolase family 65, C-terminal domain	0	1
pfam03636	Glycosyl hydrolase family 65, N-terminal domain	0	1
pfam03641	Possible lysine decarboxylase	1	2
pfam03646	FlaG protein	1	2
pfam03648	Glycosyl hydrolase family 67 N-terminus	0	1
pfam03649	Uncharacterised protein family (UPF0014)	0	1
pfam03652	Holliday junction resolvase	1	1
pfam03653	Uncharacterised protein family (UPF0093)	0	1
pfam03658	RnfH family Ubiquitin	1	1
pfam03668	P-loop ATPase protein family	1	1

pfam03692	Putative zinc- or iron-chelating domain	2	1
pfam03695	Uncharacterised protein family (UPF0149)	1	2
pfam03702	Anhydro-N-acetylmuramic acid kinase	1	1
pfam03703	Bacterial PH domain	1	0
pfam03705	CheR methyltransferase, all-alpha domain	2	6
pfam03706	Lysylphosphatidylglycerol synthase TM region	1	0
pfam03707	Bacterial signalling protein N terminal repeat	0	1
pfam03709	Orn/Lys/Arg decarboxylase, N-terminal domain	1	1
pfam03710	Glutamate-ammonia ligase adenylyltransferase	1	1
pfam03711	Orn/Lys/Arg decarboxylase, C-terminal domain	1	1
pfam03713	Domain of unknown function (DUF305)	0	1
pfam03714	Bacterial pullanase-associated domain	0	1
pfam03717	Penicillin-binding Protein dimerisation domain	2	3
pfam03719	Ribosomal protein S5, C-terminal domain	1	1
pfam03720	UDP-glucose/GDP-mannose dehydrogenase family, UDP binding domain	1	1
pfam03721	UDP-glucose/GDP-mannose dehydrogenase family, NAD binding domair	1	1
pfam03724	META domain	1	1
pfam03725	3' exoribonuclease family, domain 2	2	2
pfam03726	Polyribonucleotide nucleotidyltransferase, RNA binding domain	1	1
pfam03733	Inner membrane component domain	1	0
pfam03734	L,D-transpeptidase catalytic domain	3	2
pfam03737	Demethylmenaquinone methyltransferase	2	3
pfam03739	Predicted permease YjgP/YjgQ family	2	2
pfam03740	Pyridoxal phosphate biosynthesis protein PdxJ	1	1
pfam03741	Integral membrane protein TerC family	1	0
pfam03743	Bacterial conjugation TrbI-like protein	1	0
pfam03747	ADP-ribosylglycohydrolase	0	1
pfam03748	Flagellar basal body-associated protein FliL	3	2
pfam03749	Sugar fermentation stimulation protein	1	0
pfam03755	YicC-like family, N-terminal region	1	1
pfam03764	Elongation factor G, domain IV	2	2
pfam03772	Competence protein	1	1
pfam03773	Predicted permease	2	0
pfam03775	Septum formation inhibitor MinC, C-terminal domain	1	1

pfam03776	Septum formation topological specificity factor MinE	1	1
pfam03779	SPW repeat	0	1
pfam03781	Sulfatase-modifying factor enzyme 1	1	6
pfam03783	Curli production assembly/transport component CsgG	1	1
pfam03788	LrgA family	1	1
pfam03795	YCII-related domain	1	2
pfam03796	DnaB-like helicase C terminal domain	1	1
pfam03799	Cell division protein FtsQ	1	1
pfam03806	AbgT putative transporter family	1	0
pfam03807	NADP oxidoreductase coenzyme F420-dependent	1	1
pfam03808	Glycosyl transferase WecB/TagA/CpsF family	0	1
pfam03814	Potassium-transporting ATPase A subunit	0	1
pfam03819	MazG nucleotide pyrophosphohydrolase domain	1	0
pfam03824	High-affinity nickel-transport protein	0	1
pfam03831	PhnA domain	1	1
pfam03837	RecT family	2	0
pfam03840	Preprotein translocase SecG subunit	1	1
pfam03841	L-seryl-tRNA selenium transferase	0	1
pfam03846	Cell division inhibitor SulA	1	0
pfam03848	Tellurite resistance protein TehB	1	0
pfam03853	YjeF-related protein N-terminus	1	1
pfam03861	ANTAR domain	0	3
pfam03864	Phage major capsid protein E	0	1
pfam03869	Arc-like DNA binding domain	0	1
pfam03872	Anti sigma-E protein RseA, N-terminal domain	1	1
pfam03873	Anti sigma-E protein RseA, C-terminal domain	1	0
pfam03880	DbpA RNA binding domain	2	2
pfam03883	Peroxide stress protein YaaA	1	1
pfam03884	Domain of unknown function (DUF329)	1	1
pfam03886	ABC-type transport auxiliary lipoprotein component	0	2
pfam03888	MucB/RseB N-terminal domain	1	0
pfam03889	Domain of unknown function	1	0
pfam03891	Domain of unknown function (DUF333)	1	0
pfam03892	**Nitrate reductase cytochrome c-type subunit (NapB)**	**2**	**0**

pfam03894	D-xylulose 5-phosphate/D-fructose 6-phosphate phosphoketolase	1	0
pfam03899	ATP synthase I chain	1	1
pfam03900	Porphobilinogen deaminase, C-terminal domain	1	2
pfam03901	Alg9-like mannosyltransferase family	0	2
pfam03916	Polysulphide reductase, NrfD	3	0
pfam03918	Cytochrome C biogenesis protein	1	0
pfam03922	OmpW family	1	2
pfam03923	Uncharacterized lipoprotein	1	0
pfam03924	CHASE domain	3	7
pfam03925	SeqA protein C-terminal domain	1	0
pfam03927	**NapD protein**	**2**	**0**
pfam03928	Haem-degrading	0	2
pfam03929	PepSY-associated TM region	6	8
pfam03932	CutC family	0	1
pfam03934	Type II secretion system (T2SS), protein K	1	1
pfam03937	Flavinator of succinate dehydrogenase	1	1
pfam03938	Outer membrane protein (OmpH-like)	1	1
pfam03942	DTW domain	3	1
pfam03946	Ribosomal protein L11, N-terminal domain	1	1
pfam03947	Ribosomal Proteins L2, C-terminal domain	1	1
pfam03948	Ribosomal protein L9, C-terminal domain	1	1
pfam03949	Malic enzyme, NAD binding domain	2	2
pfam03950	tRNA synthetases class I (E and Q), anti-codon binding domain	1	1
pfam03951	Glutamine synthetase, beta-Grasp domain	1	1
pfam03952	Enolase, N-terminal domain	1	1
pfam03958	Bacterial type II/III secretion system short domain	4	3
pfam03960	ArsC family	2	2
pfam03961	Protein of unknown function (DUF342)	1	2
pfam03963	Flagellar hook capping protein - N-terminal region	2	2
pfam03965	Penicillinase repressor	0	4
pfam03966	Trm112p-like protein	1	1
pfam03968	OstA-like protein	2	2
pfam03969	AFG1-like ATPase	2	1
pfam03971	Monomeric isocitrate dehydrogenase	1	1

pfam03974	Ecotin	1	0
pfam03975	CheD chemotactic sensory transduction	1	3
pfam03976	Polyphosphate kinase 2 (PPK2)	0	2
pfam03977	Na+-transporting oxaloacetate decarboxylase beta subunit	1	0
pfam03979	Sigma-70 factor, region 1.1	1	2
pfam03989	DNA gyrase C-terminal domain, beta-propeller	2	2
pfam03992	Antibiotic biosynthesis monooxygenase	4	6
pfam03993	Domain of Unknown Function (DUF349)	0	1
pfam03994	Domain of Unknown Function (DUF350)	1	1
pfam04002	RadC-like JAB domain	2	1
pfam04011	LemA family	1	0
pfam04012	PspA/IM30 family	2	0
pfam04013	Putative SAM-dependent RNA methyltransferase	1	0
pfam04014	Antidote-toxin recognition MazE, bacterial antitoxin	1	0
pfam04018	Domain of unknown function (DUF368)	1	0
pfam04020	Mycobacterial 4 TMS phage holin, superfamily IV	0	1
pfam04023	FeoA domain	1	1
pfam04024	PspC domain	2	1
pfam04052	TolB amino-terminal domain	1	1
pfam04055	Radical SAM superfamily	17	12
pfam04060	Putative Fe-S cluster	1	1
pfam04069	Substrate binding domain of ABC-type glycine betaine transport system	1	2
pfam04072	Leucine carboxyl methyltransferase	0	1
pfam04073	Aminoacyl-tRNA editing domain	2	2
pfam04074	Domain of unknown function (DUF386)	1	0
pfam04077	DsrH like protein	1	0
pfam04079	Segregation and condensation complex subunit ScpB	1	1
pfam04085	rod shape-determining protein MreC	1	1
pfam04093	rod shape-determining protein MreD	1	1
pfam04095	Nicotinate phosphoribosyltransferase (NAPRTase) family	0	1
pfam04101	Glycosyltransferase family 28 C-terminal domain	1	2
pfam04102	SlyX	1	1
pfam04107	Glutamate-cysteine ligase family 2(GCS2)	0	1
pfam04116	Fatty acid hydroxylase superfamily	2	3

pfam04126	Cyclophilin-like	1	0
pfam04127	DNA / pantothenate metabolism flavoprotein	1	1
pfam04134	Protein of unknown function, DUF393	1	0
pfam04138	GtrA-like protein	0	1
pfam04143	Sulphur transport	0	1
pfam04151	Bacterial pre-peptidase C-terminal domain	5	0
pfam04166	Pyridoxal phosphate biosynthetic protein PdxA	1	1
pfam04168	A predicted alpha-helical domain with a conserved ER motif.	0	1
pfam04170	NlpE N-terminal domain	1	0
pfam04172	LrgB-like family	1	1
pfam04175	Protein of unknown function (DUF406)	1	0
pfam04183	IucA / IucC family	1	0
pfam04185	Phosphoesterase family	0	3
pfam04186	FxsA cytoplasmic membrane protein	1	0
pfam04187	Haem-binding uptake, Tiki superfamily, ChaN	0	1
pfam04193	PQ loop repeat	0	1
pfam04199	Putative cyclase	0	1
pfam04203	Sortase family	1	0
pfam04204	Homoserine O-succinyltransferase	1	0
pfam04205	FMN-binding domain	5	0
pfam04209	homogentisate 1,2-dioxygenase	1	0
pfam04216	Protein involved in formate dehydrogenase formation	0	1
pfam04217	Protein of unknown function, DUF412	1	0
pfam04219	Protein of unknown function, DUF	1	0
pfam04220	Der GTPase activator (YihI)	1	0
pfam04222	Protein of unknown function (DUF416)	1	0
pfam04224	Protein of unknown function, DUF417	2	0
pfam04225	Opacity-associated protein A LysM-like domain	2	0
pfam04227	Indigoidine synthase A like protein	0	1
pfam04228	Putative neutral zinc metallopeptidase	1	0
pfam04229	GrpB protein	1	0
pfam04230	Polysaccharide pyruvyl transferase	0	1
pfam04231	Endonuclease I	2	0
pfam04235	Protein of unknown function (DUF418)	1	1

pfam04239	Protein of unknown function (DUF421)	0	1
pfam04241	Protein of unknown function (DUF423)	1	0
pfam04244	Deoxyribodipyrimidine photo-lyase-related protein	1	1
pfam04245	37-kD nucleoid-associated bacterial protein	1	0
pfam04246	Positive regulator of sigma(E), RseC/MucC	1	0
pfam04247	Invasion gene expression up-regulator, SirB	1	0
pfam04248	Domain of unknown function (DUF427)	0	1
pfam04255	Protein of unknown function (DUF433)	0	2
pfam04257	Exodeoxyribonuclease V, gamma subunit	1	1
pfam04261	Dyp-type peroxidase family	1	0
pfam04262	Glutamate-cysteine ligase	1	1
pfam04264	YceI-like domain	2	4
pfam04266	ASCH domain	1	0
pfam04277	Oxaloacetate decarboxylase, gamma chain	1	0
pfam04279	Intracellular septation protein A	1	1
pfam04280	Tim44-like domain	1	1
pfam04285	Protein of unknown function (DUF444)	1	1
pfam04286	Protein of unknown function (DUF445)	0	1
pfam04287	tRNA pseudouridine synthase C	1	0
pfam04290	Tripartite ATP-independent periplasmic transporters, DctQ component	1	2
pfam04293	SpoVR like protein	1	1
pfam04295	D-galactarate dehydratase / Altronate hydrolase, C terminus	0	1
pfam04299	Putative FMN-binding domain	1	0
pfam04303	PrpF protein	1	2
pfam04304	Protein of unknown function (DUF454)	1	1
pfam04305	Protein of unknown function (DUF455)	0	1
pfam04307	LexA-binding, inner membrane-associated putative hydrolase	1	1
pfam04313	Type I restriction enzyme R protein N terminus (HSDR_N)	5	0
pfam04314	Copper chaperone PCu(A)C	1	1
pfam04316	Anti-sigma-28 factor, FlgM	2	2
pfam04317	YcjX-like family, DUF463	1	0
pfam04320	Protein with unknown function (DUF469)	1	0
pfam04321	RmlD substrate binding domain	3	2
pfam04324	BFD-like [2Fe-2S] binding domain	1	2

pfam04325	Protein of unknown function (DUF465)	2	1
pfam04326	Putative DNA-binding domain	1	0
pfam04328	Selenoprotein, putative	0	1
pfam04332	Protein of unknown function (DUF475)	0	2
pfam04333	MlaA lipoprotein	1	1
pfam04336	Protein of unknown function, DUF479	1	0
pfam04337	Protein of unknown function, DUF480	1	1
pfam04338	Protein of unknown function, DUF481	2	1
pfam04339	Peptidogalycan biosysnthesis/recognition	1	1
pfam04340	Protein of unknown function, DUF484	1	2
pfam04341	Protein of unknown function, DUF485	0	1
pfam04342	Putative member of DMT superfamily (DUF486)	1	0
pfam04344	Chemotaxis phosphatase, CheZ	1	1
pfam04345	Chorismate lyase	1	1
pfam04347	Flagellar biosynthesis protein, FliO	1	1
pfam04348	LppC putative lipoprotein	1	1
pfam04349	Periplasmic glucan biosynthesis protein, MdoG	3	0
pfam04350	Pilus assembly protein, PilO	1	1
pfam04351	Pilus assembly protein, PilP	1	1
pfam04352	ProQ/FINO family	1	1
pfam04353	Regulator of RNA polymerase sigma(70) subunit, Rsd/AlgQ	1	0
pfam04354	ZipA, C-terminal FtsZ-binding domain	1	1
pfam04355	SmpA / OmlA family	1	1
pfam04356	Protein of unknown function (DUF489)	1	0
pfam04357	TamB, inner membrane protein subunit of TAM complex	1	1
pfam04358	DsrC like protein	1	0
pfam04359	Protein of unknown function (DUF493)	1	1
pfam04361	Protein of unknown function (DUF494)	1	1
pfam04362	Bacterial Fe(2+) trafficking	1	1
pfam04364	DNA polymerase III chi subunit, HolC	1	1
pfam04365	Ribonuclease toxin, BrnT, of type II toxin-antitoxin system	0	2
pfam04366	Las17-binding protein actin regulator	1	1
pfam04367	Protein of unknown function (DUF502)	1	1
pfam04371	Porphyromonas-type peptidyl-arginine deiminase	1	0

pfam04375	HemX, putative uroporphyrinogen-III C-methyltransferase	1	1
pfam04376	Arginine-tRNA-protein transferase, N terminus	1	1
pfam04377	Arginine-tRNA-protein transferase, C terminus	1	1
pfam04378	Ribosomal RNA large subunit methyltransferase D, RlmJ	1	1
pfam04379	ApaG domain	1	1
pfam04380	Membrane fusogenic activity	1	1
pfam04381	Putative exonuclease, RdgC	1	1
pfam04384	Iron-sulphur cluster assembly	1	0
pfam04386	Stringent starvation protein B	1	1
pfam04389	Peptidase family M28	4	8
pfam04390	Lipopolysaccharide-assembly	1	1
pfam04393	Protein of unknown function (DUF535)	0	1
pfam04397	LytTr DNA-binding domain	2	14
pfam04400	(Na+)-NQR maturation NqrM	1	0
pfam04402	Protein of unknown function (DUF541)	2	3
pfam04403	Paraquat-inducible protein A	3	1
pfam04405	Domain of Unknown function (DUF542)	1	0
pfam04408	Helicase associated domain (HA2)	2	1
pfam04413	3-Deoxy-D-manno-octulosonic-acid transferase (kdotransferase)	1	1
pfam04430	Protein of unknown function (DUF498/DUF598)	0	1
pfam04434	SWIM zinc finger	0	1
pfam04442	Cytochrome c oxidase assembly protein CtaG/Cox11	1	1
pfam04445	Putative SAM-dependent methyltransferase	1	0
pfam04452	RNA methyltransferase	1	1
pfam04453	Organic solvent tolerance protein	1	1
pfam04461	Protein of unknown function (DUF520)	1	1
pfam04463	Protein of unknown function (DUF523)	2	0
pfam04464	CDP-Glycerol:Poly(glycerophosphate) glycerophosphotransferase	1	0
pfam04471	Restriction endonuclease	1	1
pfam04509	CheC-like family	0	1
pfam04519	Polymer-forming cytoskeletal	2	1
pfam04539	Sigma-70 region 3	4	5
pfam04542	Sigma-70 region 2	11	22
pfam04545	Sigma-70, region 4	6	7

pfam04546	Sigma-70, non-essential region	1	2
pfam04551	GcpE protein	1	1
pfam04552	Sigma-54, DNA binding domain	1	1
pfam04560	RNA polymerase Rpb2, domain 7	1	1
pfam04561	RNA polymerase Rpb2, domain 2	1	1
pfam04563	RNA polymerase beta subunit	1	1
pfam04565	RNA polymerase Rpb2, domain 3	1	1
pfam04606	Ogr/Delta-like zinc finger	1	0
pfam04607	Region found in RelA / SpoT proteins	3	2
pfam04608	Phosphatidylglycerophosphatase A	1	0
pfam04612	Type II secretion system (T2SS), protein M	1	1
pfam04613	UDP-3-O-[3-hydroxymyristoyl] glucosamine N-acyltransferase, LpxD	1	1
pfam04616	Glycosyl hydrolases family 43	0	9
pfam04632	Fusaric acid resistance protein family	2	3
pfam04654	Protein of unknown function, DUF599	1	0
pfam04657	Putative inner membrane exporter, YdcZ	2	0
pfam04675	DNA ligase N terminus	0	1
pfam04679	ATP dependent DNA ligase C terminal region	0	2
pfam04715	Anthranilate synthase component I, N terminal region	2	1
pfam04728	Lipoprotein leucine-zipper	1	0
pfam04748	Divergent polysaccharide deacetylase	1	0
pfam04751	Protein of unknown function (DUF615)	1	1
pfam04752	ChaC-like protein	0	1
pfam04754	Putative transposase, YhgA-like	0	1
pfam04760	Translation initiation factor IF-2, N-terminal region	1	1
pfam04773	FecR protein	1	6
pfam04820	Tryptophan halogenase	5	6
pfam04828	Glutathione-dependent formaldehyde-activating enzyme	4	4
pfam04851	Type III restriction enzyme, res subunit	9	1
pfam04860	Phage portal protein	1	0
pfam04865	Baseplate J-like protein	1	1
pfam04879	Molybdopterin oxidoreductase Fe4S4 domain	6	3
pfam04888	Secretion system effector C (SseC) like family	1	0
pfam04892	VanZ like family	1	2

pfam04898	Glutamate synthase central domain	1	1
pfam04909	Amidohydrolase	0	4
pfam04932	O-Antigen ligase	0	2
pfam04940	Sensors of blue-light using FAD	0	2
pfam04952	Succinylglutamate desuccinylase / Aspartoacylase family	5	3
pfam04954	Siderophore-interacting protein	1	1
pfam04955	HupE / UreJ protein	1	1
pfam04957	Ribosome modulation factor	1	0
pfam04958	Arginine N-succinyltransferase beta subunit	1	2
pfam04960	Glutaminase	1	0
pfam04963	Sigma-54 factor, core binding domain	1	1
pfam04964	Flp/Fap pilin component	0	2
pfam04965	Gene 25-like lysozyme	0	2
pfam04966	Carbohydrate-selective porin, OprB family	0	1
pfam04970	Lecithin retinol acyltransferase	1	0
pfam04972	BON domain	2	5
pfam04973	Nicotinamide mononucleotide transporter	2	2
pfam04977	Septum formation initiator	1	1
pfam04982	HPP family	1	1
pfam04983	RNA polymerase Rpb1, domain 3	1	1
pfam04984	Phage tail sheath protein	0	1
pfam04993	TfoX N-terminal domain	0	1
pfam04995	Heme exporter protein D (CcmD)	1	0
pfam04996	Succinylarginine dihydrolase	2	1
pfam04997	RNA polymerase Rpb1, domain 1	1	1
pfam04998	RNA polymerase Rpb1, domain 5	1	1
pfam04999	Cell division protein FtsL	1	1
pfam05000	RNA polymerase Rpb1, domain 4	1	1
pfam05013	N-formylglutamate amidohydrolase	0	1
pfam05015	RelE-like toxin of type II toxin-antitoxin system HigB	0	1
pfam05016	ParE toxin of type II toxin-antitoxin system, parDE	2	1
pfam05035	2-keto-3-deoxy-galactonokinase	0	1
pfam05036	Sporulation related domain	4	4
pfam05050	Methyltransferase FkbM domain	0	2

pfam05088	Bacterial NAD-glutamate dehydrogenase	1	1
pfam05096	Glutamine cyclotransferase	0	1
pfam05099	Tellurite resistance protein TerB	2	0
pfam05114	Protein of unknown function (DUF692)	2	1
pfam05118	Aspartyl/Asparaginyl beta-hydroxylase	1	1
pfam05125	Phage major capsid protein, P2 family	1	0
pfam05128	Domain of unknown function (DUF697)	1	0
pfam05130	FlgN protein	2	2
pfam05134	Type II secretion system (T2SS), protein L	1	1
pfam05136	Phage portal protein, lambda family	1	1
pfam05137	Fimbrial assembly protein (PilN)	1	1
pfam05138	Phenylacetic acid catabolic protein	0	2
pfam05140	ResB-like family	0	1
pfam05144	Phage replication protein CRI	2	0
pfam05154	TM2 domain	0	2
pfam05155	Phage X family	3	0
pfam05157	Type II secretion system (T2SS), protein E, N-terminal domain	2	5
pfam05161	MOFRL family	0	1
pfam05163	DinB family	1	1
pfam05164	Cell division protein ZapA	1	1
pfam05166	YcgL domain	1	0
pfam05170	AsmA family	1	2
pfam05171	Haemin-degrading HemS.ChuX domain	0	1
pfam05173	Dihydrodipicolinate reductase, C-terminus	1	1
pfam05175	Methyltransferase small domain	4	4
pfam05187	Electron transfer flavoprotein-ubiquinone oxidoreductase, 4Fe-4S	1	1
pfam05188	MutS domain II	1	1
pfam05190	MutS family domain IV	1	1
pfam05191	Adenylate kinase, active site lid	1	1
pfam05192	MutS domain III	1	1
pfam05193	Peptidase M16 inactive domain	7	5
pfam05194	UreE urease accessory protein, C-terminal domain	0	1
pfam05195	Aminopeptidase P, N-terminal domain	0	1
pfam05198	Translation initiation factor IF-3, N-terminal domain	1	1

pfam05199	GMC oxidoreductase	3	7
pfam05201	Glutamyl-tRNAGlu reductase, N-terminal domain	1	1
pfam05209	Septum formation inhibitor MinC, N-terminal domain	1	1
pfam05221	S-adenosyl-L-homocysteine hydrolase	0	1
pfam05222	Alanine dehydrogenase/PNT, N-terminal domain	2	2
pfam05226	CHASE2 domain	0	2
pfam05227	CHASE3 domain	0	6
pfam05229	Spore Coat Protein U domain	0	3
pfam05231	MASE1	1	4
pfam05232	Chlorhexidine efflux transporter	1	1
pfam05233	PHB accumulation regulatory domain	0	1
pfam05235	CHAD domain	0	1
pfam05239	PRC-barrel domain	2	3
pfam05247	Flagellar transcriptional activator (FlhD)	0	1
pfam05258	Protein of unknown function (DUF721)	1	1
pfam05280	Flagellar transcriptional activator (FlhC)	0	1
pfam05299	M61 glycyl aminopeptidase	2	4
pfam05309	TraE protein	1	0
pfam05345	Putative Ig domain	1	1
pfam05359	Domain of Unknown Function (DUF748)	1	1
pfam05360	yiaA/B two helix domain	0	1
pfam05362	Lon protease (S16) C-terminal proteolytic domain	3	1
pfam05368	NmrA-like family	0	2
pfam05378	Hydantoinase/oxoprolinase N-terminal region	0	1
pfam05400	Flagellar protein FliT	0	2
pfam05401	Nodulation protein S (NodS)	0	1
pfam05426	Alginate lyase	0	3
pfam05430	S-adenosyl-L-methionine-dependent methyltransferase	0	1
pfam05433	Glycine zipper 2TM domain	1	7
pfam05437	Branched-chain amino acid transport protein (AzlD)	2	1
pfam05488	PAAR motif	0	3
pfam05491	Holliday junction DNA helicase ruvB C-terminus	1	1
pfam05494	MlaC protein	1	1
pfam05496	Holliday junction DNA helicase ruvB N-terminus	1	1

pfam05506	Domain of unknown function (DUF756)	0	1
pfam05509	TraY domain	2	0
pfam05523	WxcM-like, C-terminal	0	2
pfam05524	PEP-utilising enzyme, N-terminal	2	1
pfam05525	Branched-chain amino acid transport protein	1	0
pfam05532	CsbD-like	1	1
pfam05534	HicB family	0	1
pfam05544	Proline racemase	0	1
pfam05545	Cbb3-type cytochrome oxidase component FixQ	1	1
pfam05547	Immune inhibitor A peptidase M6	1	0
pfam05552	Conserved TM helix	2	0
pfam05567	Neisseria PilC beta-propeller domain	1	2
pfam05569	BlaR1 peptidase M56	0	5
pfam05591	Type VI secretion system, VipA, VC_A0107 or Hcp2	0	2
pfam05593	RHS Repeat	0	1
pfam05597	Poly(hydroxyalcanoate) granule associated protein (phasin)	0	1
pfam05618	Putative ATP-dependant zinc protease	1	0
pfam05635	23S rRNA-intervening sequence protein	1	0
pfam05638	Type VI secretion system effector, Hcp	0	2
pfam05643	Putative bacterial lipoprotein (DUF799)	0	1
pfam05649	Peptidase family M13	3	4
pfam05651	Putative sugar diacid recognition	0	1
pfam05661	Protein of unknown function (DUF808)	1	1
pfam05673	Protein of unknown function (DUF815)	0	1
pfam05675	Protein of unknown function (DUF817)	0	1
pfam05681	Fumarate hydratase (Fumerase)	1	1
pfam05683	Fumarase C-terminus	1	1
pfam05684	Protein of unknown function (DUF819)	1	0
pfam05690	Thiazole biosynthesis protein ThiG	1	1
pfam05697	Bacterial trigger factor protein (TF)	1	1
pfam05698	Bacterial trigger factor protein (TF) C-terminus	1	1
pfam05707	Zonular occludens toxin (Zot)	3	0
pfam05708	Permuted papain-like amidase enzyme, YaeF/YiiX, C92 family	0	1
pfam05717	IS66 Orf2 like protein	4	0

pfam05721	Phytanoyl-CoA dioxygenase (PhyH)	1	3
pfam05724	Thiopurine S-methyltransferase (TPMT)	1	1
pfam05726	Pirin C-terminal cupin domain	2	3
pfam05728	Uncharacterised protein family (UPF0227)	2	1
pfam05742	Transport and Golgi organisation 2	1	1
pfam05746	DALR anticodon binding domain	2	2
pfam05751	FixH	1	1
pfam05762	VWA domain containing CoxE-like protein	0	1
pfam05768	Glutaredoxin-like domain (DUF836)	1	1
pfam05785	Rho-activating domain of cytotoxic necrotizing factor	1	0
pfam05787	Bacterial protein of unknown function (DUF839)	1	1
pfam05791	Bacillus haemolytic enterotoxin (HBL)	0	1
pfam05802	Enterobacterial EspB protein	1	0
pfam05840	Bacteriophage replication gene A protein (GPA)	1	0
pfam05860	haemagglutination activity domain	0	1
pfam05872	Bacterial protein of unknown function (DUF853)	1	0
pfam05876	Phage terminase large subunit (GpA)	1	1
pfam05896	Na(+)-translocating NADH-quinone reductase subunit A (NQRA)	2	0
pfam05899	Protein of unknown function (DUF861)	1	1
pfam05901	Excalibur calcium-binding domain	2	1
pfam05922	Peptidase inhibitor I9	4	0
pfam05926	Phage head completion protein (GPL)	1	0
pfam05929	Phage capsid scaffolding protein (GPO) serine peptidase	1	0
pfam05932	Tir chaperone protein (CesT) family	1	0
pfam05936	Bacterial Type VI secretion, VC_A0110, EvfL, ImpJ, VasE	0	2
pfam05940	NnrS protein	1	0
pfam05943	Type VI secretion protein, EvpB/VC_A0108, tail sheath	0	2
pfam05947	Type VI secretion system, TssF	0	2
pfam05951	Bacterial protein of unknown function (DUF882)	1	1
pfam05954	Phage late control gene D protein (GPD)	0	6
pfam05957	Bacterial protein of unknown function (DUF883)	1	2
pfam05958	tRNA (Uracil-5-)-methyltransferase	3	1
pfam05960	Bacterial protein of unknown function (DUF885)	5	6
pfam05962	HutD	1	1

pfam05971	Protein of unknown function (DUF890)	1	0
pfam05973	Phage derived protein Gp49-like (DUF891)	1	1
pfam05977	Transmembrane secretion effector	0	2
pfam05981	CreA protein	1	0
pfam05982	Na+-dependent bicarbonate transporter superfamily	1	0
pfam05985	Ethanolamine ammonia-lyase light chain (EutC)	0	1
pfam05995	Cysteine dioxygenase type I	0	1
pfam06005	Cell division protein ZapB	1	0
pfam06026	Ribose 5-phosphate isomerase A (phosphoriboisomerase A)	1	1
pfam06029	AlkA N-terminal domain	1	0
pfam06035	Bacterial transglutaminase-like cysteine proteinase BTLCP	1	0
pfam06039	Malate:quinone oxidoreductase (Mqo)	0	2
pfam06041	Bacterial protein of unknown function (DUF924)	1	1
pfam06042	Nucleotidyltransferase	1	2
pfam06056	Putative ATPase subunit of terminase (gpP-like)	1	0
pfam06057	Bacterial virulence protein (VirJ)	1	0
pfam06062	Uncharacterised protein family (UPF0231)	1	0
pfam06071	Protein of unknown function (DUF933)	1	1
pfam06073	Bacterial protein of unknown function (DUF934)	0	1
pfam06080	Protein of unknown function (DUF938)	1	0
pfam06082	Exopolysaccharide biosynthesis protein YbjH	1	0
pfam06097	Bacterial protein of unknown function (DUF945)	0	1
pfam06100	MCRA family	0	1
pfam06114	Domain of unknown function (DUF955)	2	0
pfam06122	Conjugative relaxosome accessory transposon protein	1	0
pfam06123	Inner membrane protein CreD	1	0
pfam06127	Protein of unknown function (DUF962)	1	1
pfam06144	DNA polymerase III, delta subunit	1	1
pfam06146	Phosphate-starvation-inducible E	1	0
pfam06149	Protein of unknown function (DUF969)	0	1
pfam06155	Protein of unknown function (DUF971)	1	1
pfam06165	Glycosyltransferase family 36	0	3
pfam06166	Protein of unknown function (DUF979)	0	1
pfam06167	Glucose-regulated metallo-peptidase M90	1	1

pfam06172	Cupin superfamily (DUF985)	1	0
pfam06175	tRNA-(MS[2]IO[6]A)-hydroxylase (MiaE)	1	0
pfam06181	Urate oxidase N-terminal	0	1
pfam06185	YecM protein	1	0
pfam06189	5'-nucleotidase	0	1
pfam06224	Winged helix DNA-binding domain	1	1
pfam06228	Haem utilisation ChuX/HutX	1	1
pfam06243	Phenylacetic acid degradation B	0	1
pfam06250	Protein of unknown function (DUF1016)	2	0
pfam06251	Capsule biosynthesis GfcC	2	0
pfam06271	RDD family	2	2
pfam06276	Ferric iron reductase FhuF-like transporter	2	0
pfam06283	Trehalose utilisation	0	1
pfam06287	Protein of unknown function (DUF1039)	1	0
pfam06293	Lipopolysaccharide kinase (Kdo/WaaP) family	1	0
pfam06305	Protein of unknown function (DUF1049)	1	1
pfam06306	Beta-1,4-N-acetylgalactosaminyltransferase (CgtA)	1	0
pfam06314	Acetoacetate decarboxylase (ADC)	1	1
pfam06325	Ribosomal protein L11 methyltransferase (PrmA)	1	1
pfam06347	Bacterial SH3 domain	0	1
pfam06406	StbA protein	1	0
pfam06415	BPG-independent PGAM N-terminus (iPGM_N)	1	0
pfam06418	CTP synthase N-terminus	1	1
pfam06421	GTP-binding protein LepA C-terminus	1	1
pfam06426	Serine acetyltransferase, N-terminal	0	1
pfam06429	Flagellar basal body rod FlgEFG protein C-terminal	10	11
pfam06434	Aconitate hydratase 2 N-terminus	1	1
pfam06439	Domain of Unknown Function (DUF1080)	0	1
pfam06441	Epoxide hydrolase N terminus	0	2
pfam06445	GyrI-like small molecule binding domain	0	2
pfam06450	Bacterial Na+/H+ antiporter B (NhaB)	1	0
pfam06463	Molybdenum Cofactor Synthesis C	2	1
pfam06470	SMC proteins Flexible Hinge Domain	0	1
pfam06476	Protein of unknown function (DUF1090)	1	0

pfam06480	FtsH Extracellular	1	1
pfam06481	COX Aromatic Rich Motif	1	1
pfam06483	**Chitinase C**	**1**	**0**
pfam06490	Flagellar regulatory protein FleQ	1	0
pfam06496	Protein of unknown function (DUF1097)	1	0
pfam06506	Propionate catabolism activator	0	1
pfam06508	Queuosine biosynthesis protein QueC	1	1
pfam06519	TolA C-terminal	1	0
pfam06526	Protein of unknown function (DUF1107)	1	0
pfam06527	TniQ	2	0
pfam06532	Protein of unknown function (DUF1109)	0	2
pfam06537	Di-haem oxidoreductase, putative peroxidase	0	1
pfam06574	FAD synthetase	1	1
pfam06580	Histidine kinase	2	15
pfam06611	Protein of unknown function (DUF1145)	1	0
pfam06628	Catalase-related immune-responsive	1	4
pfam06629	MltA-interacting protein MipA	3	3
pfam06667	Phage shock protein B	1	0
pfam06674	Protein of unknown function (DUF1176)	0	1
pfam06689	ClpX C4-type zinc finger	1	1
pfam06719	AraC-type transcriptional regulator N-terminus	0	6
pfam06723	MreB/Mbl protein	1	1
pfam06725	3D domain	0	1
pfam06733	DEAD_2	0	1
pfam06736	Protein of unknown function (DUF1211)	0	1
pfam06738	Putative threonine/serine exporter	2	0
pfam06744	Type VI secretion protein IcmF C-terminal	0	2
pfam06745	KaiC	0	2
pfam06750	Bacterial Peptidase A24 N-terminal domain	1	1
pfam06751	Ethanolamine ammonia lyase large subunit (EutB)	0	1
pfam06761	Intracellular multiplication and human macrophage-killing	0	2
pfam06769	YoeB-like toxin of bacterial type II toxin-antitoxin system	1	1
pfam06779	Uncharacterised MFS-type transporter YbfB	0	1
pfam06794	Uncharacterised protein family (UPF0270)	1	0

pfam06796	**Periplasmic nitrate reductase protein NapE**	**2**	**0**
pfam06798	PrkA serine protein kinase C-terminal domain	1	1
pfam06803	Protein of unknown function (DUF1232)	1	0
pfam06804	NlpB/DapX lipoprotein	1	1
pfam06806	Putative excisionase (DUF1233)	1	0
pfam06808	Tripartite ATP-independent periplasmic transporter, DctM component	2	2
pfam06812	ImpA, N-terminal, type VI secretion system	0	2
pfam06821	Serine hydrolase	0	1
pfam06826	Predicted Permease Membrane Region	0	1
pfam06827	Zinc finger found in FPG and IleRS	2	2
pfam06831	Formamidopyrimidine-DNA glycosylase H2TH domain	1	2
pfam06832	Penicillin-Binding Protein C-terminus Family	1	1
pfam06833	Malonate decarboxylase gamma subunit (MdcE)	0	1
pfam06834	TraU protein	2	0
pfam06835	Lipopolysaccharide-assembly, LptC-related	1	1
pfam06850	PHB de-polymerase C-terminus	0	1
pfam06853	Protein of unknown function (DUF1249)	1	1
pfam06857	Malonate decarboxylase delta subunit (MdcD)	0	1
pfam06865	Protein of unknown function (DUF1255)	1	1
pfam06877	Regulator of ribonuclease activity B	2	2
pfam06890	Bacteriophage Mu Gp45 protein	0	1
pfam06891	P2 phage tail completion protein R (GpR)	1	0
pfam06892	Phage regulatory protein CII (CP76)	1	1
pfam06912	Protein of unknown function (DUF1275)	0	2
pfam06930	Protein of unknown function (DUF1282)	1	0
pfam06934	Fatty acid cis/trans isomerase (CTI)	1	0
pfam06938	Protein of unknown function (DUF1285)	1	0
pfam06940	Domain of unknown function (DUF1287)	1	0
pfam06945	Protein of unknown function (DUF1289)	3	2
pfam06961	Protein of unknown function (DUF1294)	1	1
pfam06964	Alpha-L-arabinofuranosidase C-terminal domain	0	1
pfam06965	Na+/H+ antiporter 1	1	0
pfam06968	Biotin and Thiamin Synthesis associated domain	2	1
pfam06969	HemN C-terminal domain	2	2

pfam06974	Protein of unknown function (DUF1298)	0	1
pfam06980	Protein of unknown function (DUF1302)	1	1
pfam06983	3-demethylubiquinone-9 3-methyltransferase	0	3
pfam06986	Type-1V conjugative transfer system mating pair stabilisation	1	0
pfam06992	Replication protein P	1	0
pfam06993	Protein of unknown function (DUF1304)	0	1
pfam06996	Type VI secretion, TssG	0	2
pfam07007	Protein of unknown function (DUF1311)	0	3
pfam07012	Curlin associated repeat	2	0
pfam07021	Methionine biosynthesis protein MetW	0	1
pfam07022	Bacteriophage CI repressor helix-turn-helix domain	1	0
pfam07023	Protein of unknown function (DUF1315)	1	0
pfam07027	Protein of unknown function (DUF1318)	1	0
pfam07043	Protein of unknown function (DUF1328)	1	0
pfam07044	Protein of unknown function (DUF1329)	1	1
pfam07055	Enoyl reductase FAD binding domain	1	0
pfam07063	Domain of unknown function (DUF1338)	1	0
pfam07072	Protein of unknown function (DUF1342)	1	1
pfam07073	Modulator of Rho-dependent transcription termination (ROF)	1	0
pfam07077	Protein of unknown function (DUF1345)	0	1
pfam07080	Protein of unknown function (DUF1348)	0	2
pfam07085	DRTGG domain	1	0
pfam07087	Protein of unknown function (DUF1353)	0	2
pfam07103	Protein of unknown function (DUF1365)	1	1
pfam07120	Protein of unknown function (DUF1376)	0	1
pfam07126	Cell-division protein ZapC	1	0
pfam07130	YebG protein	1	0
pfam07143	CrtC N-terminal lipocalin domain	1	1
pfam07152	YaeQ protein	1	1
pfam07157	DNA circularisation protein N-terminus	0	1
pfam07167	Poly-beta-hydroxybutyrate polymerase (PhaC) N-terminus	0	1
pfam07178	TraL protein	1	0
pfam07179	SseB protein N-terminal domain	1	0
pfam07195	Flagellar hook-associated protein 2 C-terminus	2	3

pfam07196	Flagellin hook IN motif	1	0
pfam07201	HrpJ-like domain	1	0
pfam07208	Protein of unknown function (DUF1414)	1	0
pfam07209	Protein of unknown function (DUF1415)	1	1
pfam07219	HemY protein N-terminus	1	1
pfam07221	N-acylglucosamine 2-epimerase (GlcNAc 2-epimerase)	0	1
pfam07224	Chlorophyllase	0	1
pfam07228	Stage II sporulation protein E (SpoIIE)	1	2
pfam07233	Protein of unknown function (DUF1425)	1	0
pfam07235	Protein of unknown function (DUF1427)	0	1
pfam07237	Protein of unknown function (DUF1428)	0	1
pfam07238	PilZ domain	4	7
pfam07244	Surface antigen variable number repeat	3	2
pfam07264	Etoposide-induced protein 2.4 (EI24)	1	1
pfam07273	Protein of unknown function (DUF1439)	1	1
pfam07277	SapC	2	1
pfam07290	Protein of unknown function (DUF1449)	1	0
pfam07295	Zinc-ribbon containing domain	1	0
pfam07298	NnrU protein	0	1
pfam07308	Protein of unknown function (DUF1456)	1	0
pfam07317	Flagellar regulator YcgR	0	1
pfam07331	Tripartite tricarboxylate transporter TctB family	1	0
pfam07332	Putative Actinobacterial Holin-X, holin superfamily III	0	1
pfam07348	Syd protein (SUKH-2)	1	0
pfam07366	SnoaL-like polyketide cyclase	0	3
pfam07369	Protein of unknown function (DUF1488)	1	0
pfam07394	Protein of unknown function (DUF1501)	0	1
pfam07396	Phosphate-selective porin O and P	2	0
pfam07399	Putative Na+/H+ antiporter	0	1
pfam07409	Phage protein GP46	0	1
pfam07411	Domain of unknown function (DUF1508)	1	0
pfam07437	YfaZ precursor	1	0
pfam07445	Primosomal replication protein priC	1	0
pfam07452	CHRD domain	0	1

pfam07470	Glycosyl Hydrolase Family 88	0	3
pfam07471	Phage DNA packaging protein Nu1	1	1
pfam07475	HPr Serine kinase C-terminal domain	0	1
pfam07477	Glycosyl hydrolase family 67 C-terminus	0	1
pfam07478	D-ala D-ala ligase C-terminus	1	1
pfam07479	NAD-dependent glycerol-3-phosphate dehydrogenase C-terminus	1	1
pfam07484	Phage Tail Collar Domain	1	2
pfam07488	Glycosyl hydrolase family 67 middle domain	0	1
pfam07494	Two component regulator propeller	3	11
pfam07495	Y_Y_Y domain	2	19
pfam07497	Rho termination factor, RNA-binding domain	1	1
pfam07498	Rho termination factor, N-terminal domain	1	1
pfam07499	RuvA, C-terminal domain	1	1
pfam07503	HypF finger	1	0
pfam07504	Fungalysin/Thermolysin Propeptide Motif	1	1
pfam07508	Recombinase	0	3
pfam07510	Protein of unknown function (DUF1524)	1	0
pfam07514	Putative helicase	3	0
pfam07515	Putative conjugal transfer nickase/helicase TraI C-term	2	0
pfam07516	SecA Wing and Scaffold domain	1	1
pfam07517	SecA DEAD-like domain	1	1
pfam07521	Zn-dependent metallo-hydrolase RNA specificity domain	1	0
pfam07549	SecD/SecF GG Motif	4	2
pfam07559	Flagellar basal body protein FlaE	2	2
pfam07589	PEP-CTERM motif	0	30
pfam07603	Protein of unknown function (DUF1566)	1	0
pfam07610	Protein of unknown function (DUF1573)	0	1
pfam07650	KH domain	2	2
pfam07655	Secretin N-terminal domain	1	1
pfam07660	Secretin and TonB N terminus short domain	2	1
pfam07661	MORN repeat variant	5	0
pfam07662	Na+ dependent nucleoside transporter C-terminus	2	0
pfam07664	Ferrous iron transport protein B C terminus	1	1
pfam07670	Nucleoside recognition	5	2

pfam07676	WD40-like Beta Propeller Repeat	5	7
pfam07681	DoxX	2	2
pfam07683	Cobalamin synthesis protein cobW C-terminal domain	1	2
pfam07685	CobB/CobQ-like glutamine amidotransferase domain	1	2
pfam07687	Peptidase dimerisation domain	4	12
pfam07690	Major Facilitator Superfamily	29	73
pfam07694	5TMR of 5TMR-LYT	3	0
pfam07695	7TM diverse intracellular signalling	2	2
pfam07696	7TMR-DISM extracellular 2	2	1
pfam07702	UTRA domain	1	4
pfam07703	Alpha-2-macroglobulin family N-terminal region	1	1
pfam07714	Protein tyrosine kinase	1	1
pfam07715	TonB-dependent Receptor Plug Domain	33	95
pfam07717	Oligonucleotide/oligosaccharide-binding (OB)-fold	1	1
pfam07719	Tetratricopeptide repeat	1	2
pfam07721	Tetratricopeptide repeat	0	1
pfam07722	Peptidase C26	1	2
pfam07724	AAA domain (Cdc48 subfamily)	4	6
pfam07726	ATPase family associated with various cellular activities (AAA)	2	3
pfam07728	AAA domain (dynein-related subfamily)	1	0
pfam07729	FCD domain	3	6
pfam07730	Histidine kinase	3	25
pfam07731	Multicopper oxidase	0	1
pfam07732	Multicopper oxidase	0	1
pfam07733	Bacterial DNA polymerase III alpha subunit	1	3
pfam07743	HSCB C-terminal oligomerisation domain	1	1
pfam07745	Glycosyl hydrolase family 53	0	1
pfam07746	Aromatic-ring-opening dioxygenase LigAB, LigA subunit	1	1
pfam07751	Abi-like protein	2	0
pfam07769	psiF repeat	0	1
pfam07786	Protein of unknown function (DUF1624)	1	1
pfam07793	Protein of unknown function (DUF1631)	0	1
pfam07805	HipA-like N-terminal domain	0	1
pfam07811	TadE-like protein	0	5

pfam07813	LTXXQ motif family protein	1	1
pfam07819	PGAP1-like protein	0	1
pfam07831	Pyrimidine nucleoside phosphorylase C-terminal domain	1	1
pfam07840	FadR C-terminal domain	1	0
pfam07848	PaaX-like protein	0	2
pfam07853	Protein of unknown function (DUF1648)	0	1
pfam07859	alpha/beta hydrolase fold	2	6
pfam07866	Protein of unknown function (DUF1653)	0	1
pfam07869	Protein of unknown function (DUF1656)	1	2
pfam07879	PHB/PHA accumulation regulator DNA-binding domain	0	1
pfam07883	Cupin domain	2	6
pfam07916	TraG-like protein, N-terminal region	2	0
pfam07940	Heparinase II/III-like protein	0	1
pfam07943	Penicillin-binding protein 5, C-terminal domain	1	1
pfam07944	Beta-L-arabinofuranosidase, GH127	0	2
pfam07963	Prokaryotic N-terminal methylation motif	20	19
pfam07969	Amidohydrolase family	1	4
pfam07971	Glycosyl hydrolase family 92	0	2
pfam07973	Threonyl and Alanyl tRNA synthetase second additional domain	2	2
pfam07977	FabA-like domain	4	1
pfam07978	NIPSNAP	1	1
pfam07991	Acetohydroxy acid isomeroreductase, NADPH-binding domain	1	1
pfam07992	Pyridine nucleotide-disulphide oxidoreductase	8	18
pfam07993	Male sterility protein	0	1
pfam07995	Glucose / Sorbosone dehydrogenase	2	3
pfam08000	Bacterial PH domain	1	0
pfam08003	Protein of unknown function (DUF1698)	1	0
pfam08006	Protein of unknown function (DUF1700)	0	1
pfam08007	Cupin superfamily protein	2	1
pfam08013	Tagatose 6 phosphate kinase	0	1
pfam08014	Domain of unknown function (DUF1704)	1	0
pfam08019	Domain of unknown function (DUF1705)	1	1
pfam08021	Siderophore-interacting FAD-binding domain	1	2
pfam08028	Acyl-CoA dehydrogenase, C-terminal domain	0	1

pfam08029	HisG, C-terminal domain	1	0
pfam08031	Berberine and berberine like	1	0
pfam08032	RNA 2'-O ribose methyltransferase substrate binding	1	1
pfam08085	Entericidin EcnA/B family	0	1
pfam08123	Histone methylation protein DOT1	0	1
pfam08125	Mannitol dehydrogenase C-terminal domain	0	1
pfam08173	Membrane bound YbgT-like protein	1	0
pfam08206	Ribonuclease B OB domain	1	1
pfam08207	Elongation factor P (EF-P) KOW-like domain	1	1
pfam08211	Cytidine and deoxycytidylate deaminase zinc-binding region	1	0
pfam08212	Lipocalin-like domain	1	1
pfam08220	DeoR-like helix-turn-helix domain	1	3
pfam08223	PaaX-like protein C-terminal domain	0	2
pfam08238	Sel1 repeat	5	9
pfam08239	Bacterial SH3 domain	1	2
pfam08240	Alcohol dehydrogenase GroES-like domain	6	11
pfam08241	Methyltransferase domain	5	6
pfam08242	Methyltransferase domain	1	0
pfam08245	Mur ligase middle domain	6	10
pfam08264	Anticodon-binding domain of tRNA	3	4
pfam08267	Cobalamin-independent synthase, N-terminal domain	1	1
pfam08272	Topoisomerase I zinc-ribbon-like	1	0
pfam08274	PhnA Zinc-Ribbon	1	1
pfam08275	DNA primase catalytic core, N-terminal domain	1	1
pfam08278	DNA primase DnaG DnaB-binding	1	1
pfam08279	HTH domain	4	1
pfam08281	Sigma-70, region 4	4	16
pfam08282	haloacid dehalogenase-like hydrolase	1	1
pfam08298	PrkA AAA domain	1	2
pfam08299	Bacterial dnaA protein helix-turn-helix	1	1
pfam08323	Starch synthase catalytic domain	1	0
pfam08327	Activator of Hsp90 ATPase homolog 1-like protein	1	2
pfam08328	Adenylosuccinate lyase C-terminal	1	1
pfam08329	Chitinase A	1	0

pfam08331	Domain of unknown function (DUF1730)	1	1
pfam08334	Type II secretion system (T2SS), protein G	1	2
pfam08335	GlnD PII-uridylyltransferase	2	2
pfam08338	Domain of unknown function (DUF1731)	1	1
pfam08340	Domain of unknown function (DUF1732)	1	1
pfam08345	Flagellar M-ring protein C-terminal	2	3
pfam08346	AntA/AntB antirepressor	0	1
pfam08348	YheO-like PAS domain	1	1
pfam08349	Protein of unknown function (DUF1722)	1	0
pfam08352	Oligopeptide/dipeptide transporter, C-terminal region	1	1
pfam08357	SEFIR domain	0	1
pfam08359	YsiA-like protein, C-terminal region	0	1
pfam08362	YcdC-like protein, C-terminal region	0	1
pfam08364	Bacterial translation initiation factor IF-2 associated region	1	1
pfam08376	Nitrate and nitrite sensing	2	0
pfam08378	Nuclease-related domain	1	0
pfam08379	Bacterial transglutaminase-like N-terminal region	0	1
pfam08386	TAP-like protein	1	1
pfam08388	Group II intron, maturase-specific domain	3	0
pfam08402	TOBE domain	2	5
pfam08411	Exonuclease C-terminal	1	1
pfam08436	1-deoxy-D-xylulose 5-phosphate reductoisomerase C-terminal	1	1
pfam08442	ATP-grasp domain	1	1
pfam08443	RimK-like ATP-grasp domain	3	1
pfam08446	PAS fold	0	2
pfam08447	PAS fold	17	22
pfam08448	PAS fold	5	18
pfam08450	SMP-30/Gluconolaconase/LRE-like region	0	7
pfam08453	Peptidase family M9 N-terminal	1	0
pfam08459	UvrC Helix-hairpin-helix N-terminal	1	1
pfam08461	Ribonuclease R winged-helix domain	1	0
pfam08468	Methyltransferase small domain N-terminal	1	0
pfam08478	POTRA domain, FtsQ-type	1	1
pfam08479	POTRA domain, ShlB-type	0	1

pfam08482	ATP-dependent helicase C-terminal	1	0
pfam08487	Vault protein inter-alpha-trypsin domain	1	1
pfam08494	DEAD/H associated	0	2
pfam08496	Peptidase family S49 N-terminal	1	0
pfam08497	Radical SAM N-terminal	1	1
pfam08501	Shikimate dehydrogenase substrate binding domain	1	1
pfam08502	LeuA allosteric (dimerisation) domain	1	2
pfam08521	**Two-component sensor kinase N-terminal**	**1**	**2**
pfam08525	Opacity-associated protein A N-terminal motif	1	0
pfam08529	NusA N-terminal domain	1	1
pfam08530	X-Pro dipeptidyl-peptidase C-terminal non-catalytic domain	0	3
pfam08534	Redoxin	7	1
pfam08541	3-Oxoacyl-[acyl-carrier-protein (ACP)] synthase III C terminal	3	2
pfam08543	Phosphomethylpyrimidine kinase	1	2
pfam08544	GHMP kinases C terminal	2	1
pfam08545	3-Oxoacyl-[acyl-carrier-protein (ACP)] synthase III	2	2
pfam08546	Ketopantoate reductase PanE/ApbA C terminal	1	1
pfam08547	Complex I intermediate-associated protein 30 (CIA30)	1	0
pfam08548	Peptidase M10 serralysin C terminal	0	2
pfam08645	Polynucleotide kinase 3 phosphatase	1	1
pfam08659	KR domain	1	0
pfam08666	SAF domain	0	4
pfam08668	HDOD domain	7	15
pfam08669	Glycine cleavage T-protein C-terminal barrel domain	1	1
pfam08676	MutL C terminal dimerisation domain	1	1
pfam08681	Protein of unknown function (DUF1778)	1	0
pfam08706	D5 N terminal like	0	1
pfam08708	Primase C terminal 1 (PriCT-1)	0	1
pfam08719	Domain of unknown function (DUF1768)	0	1
pfam08721	TnsA endonuclease C terminal	2	0
pfam08722	TnsA endonuclease N terminal	2	0
pfam08732	HIM1	1	0
pfam08768	Domain of unknown function (DUF1794)	0	1
pfam08770	Sulphur oxidation protein SoxZ	0	1

pfam08774	VRR-NUC domain	0	1
pfam08787	Alginate lyase	0	1
pfam08808	RES domain	0	1
pfam08811	Protein of unknown function (DUF1800)	0	1
pfam08818	Domain of unknown function (DU1801)	2	2
pfam08843	Nucleotidyl transferase AbiEii toxin, Type IV TA system	1	1
pfam08867	FRG domain	1	0
pfam08878	Domain of unknown function (DUF1837)	1	0
pfam08886	Glutamate-cysteine ligase	0	1
pfam08891	YfcL protein	1	0
pfam08895	Domain of unknown function (DUF1840)	0	1
pfam08896	Domain of unknown function (DUF1842)	0	2
pfam08897	Domain of unknown function (DUF1841)	0	1
pfam08900	Domain of unknown function (DUF1845)	2	0
pfam08905	Domain of unknown function (DUF1850)	1	0
pfam08907	Domain of unknown function (DUF1853)	0	1
pfam08908	Domain of unknown function (DUF1852)	1	0
pfam08909	Domain of unknown function (DUF1854)	0	1
pfam08921	Domain of unknown function (DUF1904)	1	0
pfam08924	Domain of unknown function (DUF1906)	0	1
pfam08933	Domain of unknown function (DUF1864)	1	0
pfam08982	Domain of unknown function (DUF1857)	0	2
pfam08985	DP-EP family	1	0
pfam09000	Cytotoxic	0	1
pfam09037	Stf0 sulphotransferase	1	0
pfam09059	TyeA	1	0
pfam09078	CheY binding	0	1
pfam09084	NMT1/THI5 like	0	9
pfam09086	Domain of unknown function (DUF1924)	1	0
pfam09087	Cyclomaltodextrinase, N-terminal	1	1
pfam09106	Elongation factor SelB, winged helix	0	1
pfam09107	Elongation factor SelB, winged helix	0	1
pfam09115	DNA polymerase III, delta subunit, C terminal	0	1
pfam09119	SicP binding	1	0

pfam09127	Leukotriene A4 hydrolase, C-terminal	2	1
pfam09137	Glucodextranase, domain N	1	0
pfam09157	Pseudouridine synthase II TruB, C-terminal	1	1
pfam09163	Formate dehydrogenase N, transmembrane	0	1
pfam09179	TilS substrate binding domain	1	1
pfam09186	Domain of unknown function (DUF1949)	1	1
pfam09190	DALR domain	1	1
pfam09242	Flavocytochrome c sulphide dehydrogenase, flavin-binding	0	1
pfam09250	Bifunctional DNA primase/polymerase, N-terminal	0	1
pfam09278	MerR, DNA binding	2	3
pfam09285	Elongation factor P, C-terminal	1	1
pfam09286	Pro-kumamolisin, activation domain	0	1
pfam09297	NADH pyrophosphatase zinc ribbon domain	0	1
pfam09299	Mu transposase, C-terminal	1	1
pfam09312	SurA N-terminal domain	1	1
pfam09313	Domain of unknown function (DUF1971)	1	0
pfam09317	Domain of unknown function (DUF1974)	2	1
pfam09334	tRNA synthetases class I (M)	1	2
pfam09335	SNARE associated Golgi protein	3	4
pfam09339	IclR helix-turn-helix domain	0	3
pfam09345	Domain of unknown function (DUF1987)	0	1
pfam09349	OHCU decarboxylase	0	1
pfam09351	Domain of unknown function (DUF1993)	1	2
pfam09361	Phasin protein	0	6
pfam09363	XFP C-terminal domain	1	0
pfam09364	XFP N-terminal domain	1	0
pfam09365	Conserved hypothetical protein (DUF2461)	1	1
pfam09371	Tex-like protein N-terminal domain	1	1
pfam09382	RQC domain	1	1
pfam09391	Protein of unknown function (DUF2000)	1	0
pfam09392	Type III secretion needle MxiH, YscF, SsaG, EprI, PscF, EscF	1	0
pfam09397	Ftsk gamma domain	1	2
pfam09411	Lipid A 3-O-deacylase (PagL)	0	1
pfam09413	Putative prokaryotic signal transducing protein	1	0

pfam09423	PhoD-like phosphatase	2	3
pfam09424	Yqey-like protein	1	1
pfam09471	IgA Peptidase M64	0	1
pfam09493	Tryptophan-rich protein (DUF2389)	1	1
pfam09500	Putative thioesterase (yiiD_Cterm)	1	0
pfam09509	Protein of unknown function (Hypoth_ymh)	0	2
pfam09523	Protein of unknown function (DUF2390)	1	0
pfam09526	Probable metal-binding protein (DUF2387)	1	0
pfam09558	Protein of unknown function (DUF2375)	1	0
pfam09601	Protein of unknown function (DUF2459)	0	1
pfam09604	F subunit of K+-transporting ATPase (Potass_KdpF)	0	1
pfam09619	Type III secretion system lipoprotein chaperone (YscW)	2	0
pfam09622	Putative integral membrane protein (DUF2391)	1	0
pfam09626	Dihaem cytochrome c	1	0
pfam09650	Putative polyhydroxyalkanoic acid system protein (PHA_gran_rgn)	0	1
pfam09656	Putative transmembrane protein (PGPGW)	1	0
pfam09673	Type-F conjugative transfer system pilin assembly protein	1	0
pfam09676	Type IV conjugative transfer system lipoprotein (TraV)	1	0
pfam09685	Domain of unknown function (DUF4870)	1	0
pfam09686	Plasmid protein of unknown function (Plasmid_RAQPRD)	1	0
pfam09694	Bacterial protein of unknown function (Gcw_chp)	4	3
pfam09699	Doubled CXXCH motif (Paired_CXXCH_1)	2	0
pfam09721	Transmembrane exosortase (Exosortase_EpsH)	0	1
pfam09722	Protein of unknown function (DUF2384)	0	1
pfam09723	Zinc ribbon domain	0	1
pfam09791	Oxidoreductase-like protein, N-terminal	0	1
pfam09821	C-terminal AAA-associated domain	0	1
pfam09829	Uncharacterized protein conserved in bacteria (DUF2057)	3	0
pfam09831	Uncharacterized protein conserved in bacteria (DUF2058)	1	1
pfam09834	Predicted membrane protein (DUF2061)	1	0
pfam09835	Uncharacterized protein conserved in bacteria (DUF2062)	1	0
pfam09836	Putative DNA-binding domain	2	1
pfam09838	Uncharacterized protein conserved in bacteria (DUF2065)	1	0
pfam09839	Uncharacterized protein conserved in bacteria (DUF2066)	1	0

pfam09842	Predicted membrane protein (DUF2069)	1	1
pfam09850	Type VI secretion system protein DotU	0	2
pfam09851	Short C-terminal domain	0	1
pfam09859	Oxygenase, catalysing oxidative methylation of damaged DNA	0	1
pfam09867	Uncharacterized protein conserved in bacteria (DUF2094)	0	2
pfam09904	Winged helix-turn helix	1	0
pfam09905	DNA-binding protein VF530	2	1
pfam09906	Uncharacterized protein conserved in bacteria (DUF2135)	0	1
pfam09907	HigB_toxin, RelE-like toxic component of a toxin-antitoxin system	1	0
pfam09912	Uncharacterized protein conserved in bacteria (DUF2141)	1	1
pfam09916	Uncharacterized protein conserved in bacteria (DUF2145)	0	1
pfam09917	Uncharacterized protein conserved in bacteria (DUF2147)	0	1
pfam09922	Cell wall-active antibiotics response 4TMS YvqF	0	1
pfam09924	Uncharacterised conserved protein (DUF2156)	1	0
pfam09925	Predicted membrane protein (DUF2157)	1	1
pfam09935	Protein of unknown function (DUF2167)	0	1
pfam09938	Uncharacterized protein conserved in bacteria (DUF2170)	1	0
pfam09945	Predicted membrane protein (DUF2177)	0	1
pfam09956	Uncharacterized conserved protein (DUF2190)	1	0
pfam09976	Tetratricopeptide repeat-like domain	1	1
pfam09983	Uncharacterized protein conserved in bacteria C-term(DUF2220)	0	1
pfam09984	Uncharacterised signal transduction histidine kinase domain (DUF2222)	1	1
pfam09994	Uncharacterized alpha/beta hydrolase domain (DUF2235)	1	2
pfam09995	Uncharacterized protein conserved in bacteria (DUF2236)	0	1
pfam09997	Predicted membrane protein (DUF2238)	1	0
pfam10009	Uncharacterized protein conserved in bacteria (DUF2252)	0	1
pfam10011	Predicted membrane protein (DUF2254)	1	0
pfam10013	Uncharacterized protein conserved in bacteria (DUF2256)	0	1
pfam10014	2OG-Fe dioxygenase	1	1
pfam10017	Histidine-specific methyltransferase, SAM-dependent	0	1
pfam10022	Uncharacterized protein conserved in bacteria (DUF2264)	0	1
pfam10023	Putative aminopeptidase	0	1
pfam10029	Predicted periplasmic protein (DUF2271)	1	1
pfam10048	Predicted integral membrane protein (DUF2282)	0	1

pfam10052	Protein of unknown function (DUF2288)	0	1
pfam10071	Zn-ribbon-containing, possibly nucleic-acid-binding protein (DUF2310)	1	0
pfam10076	Uncharacterised protein conserved in bacteria (DUF2313)	0	1
pfam10077	Uncharacterized protein conserved in bacteria (DUF2314)	0	1
pfam10084	Uncharacterized protein conserved in bacteria (DUF2322)	0	1
pfam10087	Uncharacterized protein conserved in bacteria (DUF2325)	0	2
pfam10091	Putative glucoamylase	0	3
pfam10093	Uncharacterized protein conserved in bacteria (DUF2331)	1	1
pfam10095	Uncharacterized protein conserved in bacteria (DUF2333)	1	0
pfam10099	Anti-sigma-K factor rskA	0	1
pfam10101	Predicted membrane protein (DUF2339)	1	1
pfam10106	Uncharacterized protein conserved in bacteria (DUF2345)	0	5
pfam10109	Phage tail assembly chaperone proteins, E, or 41 or 14	0	1
pfam10110	Membrane domain of glycerophosphoryl diester phosphodiesterase	1	0
pfam10119	Predicted methyltransferase regulatory domain	0	1
pfam10135	Rod binding protein	2	2
pfam10136	Site-specific recombinase	0	1
pfam10144	Bacterial virulence factor haemolysin	1	0
pfam10145	Phage-related minor tail protein	2	0
pfam10150	Ribonuclease E/G family	2	2
pfam10263	SprT-like family	1	0
pfam10276	Zinc-finger domain	0	1
pfam10282	Lactonase, 7-bladed beta-propeller	0	3
pfam10294	Lysine methyltransferase	1	0
pfam10335	Putative nucleotidyltransferase substrate binding domain	1	0
pfam10369	Small subunit of acetolactate synthase	1	1
pfam10385	RNA polymerase beta subunit external 1 domain	1	1
pfam10396	GTP-binding protein TrmE N-terminus	1	1
pfam10399	Ubiquitinol-cytochrome C reductase Fe-S subunit TAT signal	1	0
pfam10410	DnaB-helicase binding domain of primase	0	1
pfam10411	Disulfide bond isomerase protein N-terminus	1	1
pfam10412	Type IV secretion-system coupling protein DNA-binding domain	1	0
pfam10414	Sirohaem synthase dimerisation region	1	0
pfam10415	Fumarase C C-terminus	2	2

pfam10417	C-terminal domain of 1-Cys peroxiredoxin	2	1
pfam10423	Bacterial AMP nucleoside phosphorylase N-terminus	0	1
pfam10431	C-terminal, D2-small domain, of ClpB protein	4	5
pfam10438	Cyclo-malto-dextrinase C-terminal domain	1	1
pfam10458	Valyl tRNA synthetase tRNA binding arm	1	1
pfam10459	Peptidase S46	2	2
pfam10502	Signal peptidase, peptidase S26	3	3
pfam10503	Esterase PHB depolymerase	0	4
pfam10509	Galactokinase galactose-binding signature	1	0
pfam10518	TAT (twin-arginine translocation) pathway signal sequence	4	0
pfam10531	SLBB domain	2	2
pfam10555	Phospho-N-acetylmuramoyl-pentapeptide-transferase signature 1	1	1
pfam10566	Glycoside hydrolase 97	1	3
pfam10576	Iron-sulfur binding domain of endonuclease III	1	0
pfam10588	NADH-ubiquinone oxidoreductase-G iron-sulfur binding region	0	1
pfam10589	NADH-ubiquinone oxidoreductase-F iron-sulfur binding region	0	1
pfam10590	Pyridoxine 5'-phosphate oxidase C-terminal dimerisation region	1	1
pfam10592	AIPR protein	0	1
pfam10604	Polyketide cyclase / dehydrase and lipid transport	1	2
pfam10605	3HB-oligomer hydrolase (3HBOH)	0	1
pfam10609	NUBPL iron-transfer P-loop NTPase	2	1
pfam10614	Type VIII secretion system (T8SS), CsgF protein	1	0
pfam10617	Protein of unknown function (DUF2474)	1	1
pfam10618	Phage tail tube protein	0	1
pfam10620	Phosphoribosyl-dephospho-CoA transferase MdcG	0	1
pfam10627	Curli assembly protein CsgE	1	0
pfam10636	Hemin uptake protein hemP	0	1
pfam10670	Domain of unknown function (DUF4198)	1	1
pfam10672	S-adenosylmethionine-dependent methyltransferase	4	0
pfam10675	Protein of unknown function (DUF2489)	1	0
pfam10678	Protein of unknown function (DUF2492)	1	0
pfam10679	Protein of unknown function (DUF2491)	0	1
pfam10685	Stress-induced bacterial acidophilic repeat motif	0	3
pfam10688	Bacterial inner membrane protein	2	0

pfam10709	Protein of unknown function (DUF2511)	1	0
pfam10719	Late competence development protein ComFB	1	0
pfam10727	Rossmann-like domain	0	1
pfam10728	Domain of unknown function (DUF2520)	0	1
pfam10734	Protein of unknown function (DUF2523)	3	0
pfam10758	Protein of unknown function (DUF2586)	1	0
pfam10761	Protein of unknown function (DUF2590)	1	0
pfam10772	Protein of unknown function (DUF2597)	1	0
pfam10795	Protein of unknown function (DUF2607)	1	0
pfam10816	Domain of unknown function (DUF2760)	0	1
pfam10881	Protein of unknown function (DUF2726)	2	1
pfam10882	Bacterial PH domain	1	0
pfam10928	Protein of unknown function (DUF2810)	1	0
pfam10932	Protein of unknown function (DUF2783)	0	1
pfam10940	Protein of unknown function (DUF2618)	1	0
pfam10947	Protein of unknown function (DUF2628)	0	1
pfam10948	Protein of unknown function (DUF2635)	0	1
pfam10963	Phage tail assembly chaperone	1	0
pfam10972	Peptidoglycan-binding protein, CsiV	1	0
pfam10973	Protein of unknown function (DUF2799)	1	1
pfam10974	Protein of unknown function (DUF2804)	1	1
pfam10975	Protein of unknown function (DUF2802)	1	0
pfam10977	Protein of unknown function (DUF2797)	1	0
pfam10980	Protein of unknown function (DUF2787)	1	0
pfam10981	Protein of unknown function (DUF2788)	1	0
pfam10982	Protein of unknown function (DUF2789)	1	0
pfam10987	Protein of unknown function (DUF2806)	1	0
pfam10988	Putative auto-transporter adhesin, head GIN domain	0	7
pfam10990	Protein of unknown function (DUF2809)	1	1
pfam10993	Protein of unknown function (DUF2818)	0	1
pfam10996	Beta-Casp domain	1	0
pfam10997	Protein of unknown function (DUF2837)	0	1
pfam11005	Protein of unknown function (DUF2844)	0	1
pfam11006	Protein of unknown function (DUF2845)	1	0

pfam11026	Protein of unknown function (DUF2721)	1	1
pfam11042	Protein of unknown function (DUF2750)	2	0
pfam11047	Salmonella outer protein D	1	0
pfam11062	Protein of unknown function (DUF2863)	0	2
pfam11066	Protein of unknown function (DUF2867)	0	1
pfam11067	Protein of unknown function (DUF2868)	0	1
pfam11072	Protein of unknown function (DUF2859)	1	0
pfam11075	Protein of unknown function VcgC/VcgE (DUF2780)	1	0
pfam11086	Protein of unknown function (DUF2878)	1	0
pfam11101	Protein of unknown function (DUF2884)	1	0
pfam11102	Group 4 capsule polysaccharide lipoprotein gfcB, YjbF	1	0
pfam11104	Type IV pilus assembly protein PilM;	1	1
pfam11112	Pyocin activator protein PrtN	1	0
pfam11127	Protein of unknown function (DUF2892)	1	0
pfam11130	F pilus assembly Type-IV secretion system for plasmid transfer	2	0
pfam11137	Protein of unknown function (DUF2909)	1	1
pfam11140	Protein of unknown function (DUF2913)	4	0
pfam11143	Protein of unknown function (DUF2919)	1	0
pfam11150	Protein of unknown function (DUF2927)	1	0
pfam11157	Protein of unknown function (DUF2937)	1	0
pfam11159	Protein of unknown function (DUF2939)	0	1
pfam11161	Protein of unknown function (DUF2946)	0	1
pfam11162	Protein of unknown function (DUF2946)	0	4
pfam11163	Protein of unknown function (DUF2947)	1	0
pfam11168	Protein of unknown function (DUF2955)	1	0
pfam11169	Protein of unknown function (DUF2956)	1	0
pfam11172	Protein of unknown function (DUF2959)	1	0
pfam11173	Protein of unknown function (DUF2960)	1	0
pfam11174	Protein of unknown function (DUF2970)	1	1
pfam11185	Protein of unknown function (DUF2971)	1	1
pfam11188	Protein of unknown function (DUF2975)	0	1
pfam11190	Protein of unknown function (DUF2976)	1	0
pfam11193	Protein of unknown function (DUF2812)	0	1
pfam11197	Protein of unknown function (DUF2835)	1	0

pfam11198	Protein of unknown function (DUF2857)	2	0
pfam11201	Protein of unknown function (DUF2982)	1	0
pfam11205	Protein of unknown function (DUF2987)	1	0
pfam11207	Protein of unknown function (DUF2989)	1	0
pfam11212	Protein of unknown function (DUF2999)	1	0
pfam11215	Protein of unknown function (DUF3010)	1	0
pfam11216	Protein of unknown function (DUF3012)	1	0
pfam11219	Protein of unknown function (DUF3014)	1	0
pfam11227	Protein of unknown function (DUF3025)	0	1
pfam11230	Protein of unknown function (DUF3029)	1	0
pfam11231	Protein of unknown function (DUF3034)	1	1
pfam11269	Protein of unknown function (DUF3069)	1	0
pfam11281	Protein of unknown function (DUF3083)	1	0
pfam11286	Protein of unknown function (DUF3087)	1	0
pfam11301	Protein of unknown function (DUF3103)	1	0
pfam11304	Protein of unknown function (DUF3106)	0	1
pfam11306	Protein of unknown function (DUF3108)	1	2
pfam11335	Protein of unknown function (DUF3137)	3	0
pfam11339	Protein of unknown function (DUF3141)	0	1
pfam11340	Protein of unknown function (DUF3142)	1	0
pfam11342	Protein of unknown function (DUF3144)	2	0
pfam11346	Protein of unknown function (DUF3149)	1	0
pfam11348	Protein of unknown function (DUF3150)	1	0
pfam11355	Protein of unknown function (DUF3157)	1	0
pfam11356	Type II secretion system protein C	1	1
pfam11391	Protein of unknown function (DUF2798)	1	0
pfam11399	Protein of unknown function (DUF3192)	2	0
pfam11412	Disulphide bond corrector protein DsbC	3	2
pfam11426	Tn7 transposition regulator TnsC	1	0
pfam11444	Protein of unknown function (DUF2895)	1	0
pfam11446	Protein of unknown function (DUF2897)	1	0
pfam11454	Protein of unknown function (DUF3016)	1	1
pfam11456	Protein of unknown function (DUF3019)	2	0
pfam11528	Protein of unknown function (DUF3224)	1	0

pfam11557	Solitary outer membrane autotransporter beta-barrel domain	1	0
pfam11563	Protoglobin	1	1
pfam11575	FhuF 2Fe-2S C-terminal domain	1	0
pfam11582	Protein of unknown function (DUF3240)	1	0
pfam11612	Type II secretion system (T2SS), protein J	1	0
pfam11614	IG-like fold at C-terminal of FixG, putative oxidoreductase	2	1
pfam11638	DnaA N-terminal domain	1	1
pfam11659	Protein of unknown function (DUF3261)	1	0
pfam11660	Protein of unknown function (DUF3262)	1	0
pfam11661	Protein of unknown function (DUF2986)	2	0
pfam11679	Protein of unknown function (DUF3275)	1	0
pfam11697	Protein of unknown function (DUF3293)	1	0
pfam11730	Protein of unknown function (DUF3297)	1	1
pfam11731	Pathogenicity locus	1	0
pfam11734	TilS substrate C-terminal domain	1	1
pfam11737	Protein of unknown function (DUF3300)	1	1
pfam11739	Dicarboxylate transport	1	0
pfam11740	Plasmid replication region DNA-binding N-term	1	2
pfam11741	AMIN domain	1	2
pfam11742	Protein of unknown function (DUF3302)	1	0
pfam11743	Protein of unknown function (DUF3301)	1	0
pfam11745	Protein of unknown function (DUF3304)	0	4
pfam11748	Protein of unknown function (DUF3306)	1	0
pfam11749	Protein of unknown function (DUF3305)	1	0
pfam11752	Protein of unknown function (DUF3309)	1	1
pfam11755	Protein of unknown function (DUF3311)	0	1
pfam11756	Nitrous oxide-stimulated promoter	1	0
pfam11760	Cobalamin synthesis G N-terminal	0	1
pfam11761	Cobalamin biosynthesis central region	0	1
pfam11776	Nickel/cobalt transporter regulator	0	1
pfam11782	Protein of unknown function (DUF3319)	1	0
pfam11791	Aconitate B N-terminal domain	1	1
pfam11798	IMS family HHH motif	1	1
pfam11799	impB/mucB/samB family C-terminal domain	3	1

pfam11804	Protein of unknown function (DUF3325)	3	2
pfam11808	Domain of unknown function (DUF3329)	1	1
pfam11813	Protein of unknown function (DUF3334)	1	1
pfam11818	C-terminal domain of tail specific protease (DUF3340)	1	1
pfam11838	ERAP1-like C-terminal domain	1	3
pfam11842	Domain of unknown function (DUF3362)	1	1
pfam11849	Domain of unknown function (DUF3369)	5	3
pfam11852	Domain of unknown function (DUF3372)	0	2
pfam11854	Putative outer membrane beta-barrel porin, MtrB/PioB	2	0
pfam11859	Protein of unknown function (DUF3379)	1	0
pfam11867	Domain of unknown function (DUF3387)	1	0
pfam11869	Protein of unknown function (DUF3389)	1	0
pfam11871	Domain of unknown function (DUF3391)	3	1
pfam11872	Protein of unknown function (DUF3392)	1	0
pfam11880	Domain of unknown function (DUF3400)	0	1
pfam11890	Domain of unknown function (DUF3410)	1	0
pfam11892	Domain of unknown function (DUF3412)	1	1
pfam11893	Domain of unknown function (DUF3413)	1	0
pfam11898	Domain of unknown function (DUF3418)	1	1
pfam11902	Protein of unknown function (DUF3422)	0	1
pfam11903	ParD-like antitoxin of type II bacterial toxin-antitoxin system	0	2
pfam11906	Protein of unknown function (DUF3426)	0	1
pfam11907	Domain of unknown function (DUF3427)	1	0
pfam11918	N-terminal domain of Peptidase_S41 in eukaryotic IRBP	0	1
pfam11920	Protein of unknown function (DUF3438)	1	0
pfam11925	Protein of unknown function (DUF3443)	0	1
pfam11932	Protein of unknown function (DUF3450)	1	0
pfam11940	Domain of unknown function (DUF3458)	1	1
pfam11943	Protein of unknown function (DUF3460)	0	1
pfam11949	Protein of unknown function (DUF3466)	1	0
pfam11953	Domain of unknown function (DUF3470)	0	1
pfam11954	Domain of unknown function (DUF3471)	0	1
pfam11964	SpoIIAA-like	3	0
pfam11967	Recombination protein O N terminal	1	1

pfam11969	Scavenger mRNA decapping enzyme C-term binding	1	0
pfam11973	NQRA C-terminal domain	2	0
pfam11974	Alpha-2-macroglobulin MG1 domain	1	1
pfam11981	Domain of unknown function (DUF3482)	0	1
pfam11984	Protein of unknown function (DUF3485)	0	1
pfam11987	Translation-initiation factor 2	1	1
pfam11990	Protein of unknown function (DUF3487)	1	0
pfam11992	Domain of unknown function (DUF3488)	1	1
pfam11993	Ribosomal S4P (gammaproteobacterial)	1	0
pfam12002	MgsA AAA+ ATPase C terminal	1	1
pfam12006	Protein of unknown function (DUF3500)	0	1
pfam12008	Type I restriction and modification enzyme - subunit R C terminal	4	0
pfam12019	Type II transport protein GspH	3	2
pfam12034	Domain of unknown function (DUF3520)	1	0
pfam12048	Protein of unknown function (DUF3530)	1	0
pfam12065	Protein of unknown function (DUF3545)	1	0
pfam12069	Protein of unknown function (DUF3549)	1	0
pfam12083	Domain of unknown function (DUF3560)	1	0
pfam12091	Protein of unknown function (DUF3567)	0	1
pfam12094	Protein of unknown function (DUF3570)	1	1
pfam12101	Protein of unknown function (DUF3577)	2	0
pfam12112	Protein of unknown function (DUF3579)	0	1
pfam12118	SprA-related family	1	1
pfam12119	Protein of unknown function (DUF3581)	1	0
pfam12122	Cytoplasmic N-terminal domain of rhomboid serine protease	1	0
pfam12128	Protein of unknown function (DUF3584)	1	0
pfam12137	RNA polymerase recycling family C-terminal	1	0
pfam12146	Serine aminopeptidase, S33	3	8
pfam12156	Putative metal-binding domain of cation transport ATPase	1	1
pfam12161	HsdM N-terminal domain	5	0
pfam12167	Domain of unknown function (DUF3596)	1	0
pfam12169	DNA polymerase III subunits gamma and tau domain III	1	1
pfam12170	DNA polymerase III tau subunit V interacting with alpha	1	0
pfam12200	Domain of unknown function (DUF3597)	0	1

pfam12221	Bacterial membrane protein N terminal	1	1
pfam12241	Trans-2-enoyl-CoA reductase catalytic region	1	0
pfam12242	NAD(P)H binding domain of trans-2-enoyl-CoA reductase	1	0
pfam12244	Protein of unknown function (DUF3606)	0	1
pfam12245	Bacterial Ig-like domain (group 3)	2	0
pfam12262	Bacterial virulence factor lipase N-terminal	1	0
pfam12268	Protein of unknown function (DUF3612)	1	0
pfam12276	Protein of unknown function (DUF3617)	0	1
pfam12279	Protein of unknown function (DUF3619)	0	1
pfam12286	Protein of unknown function (DUF3622)	1	0
pfam12290	Protein of unknown function (DUF3802)	1	0
pfam12292	Protein of unknown function (DUF3624)	1	0
pfam12294	Protein of unknown function (DUF3626)	1	0
pfam12305	Protein of unknown function (DUF3630)	1	0
pfam12306	Inclusion body protein	1	0
pfam12320	Type 5 capsule protein repressor C-terminal domain	1	1
pfam12321	Protein of unknown function (DUF3634)	1	0
pfam12327	FtsZ family, C-terminal domain	1	1
pfam12339	DNA-J related protein	1	0
pfam12344	Ultra-violet resistance protein B	1	1
pfam12365	Protein of unknown function (DUF3649)	1	2
pfam12392	Collagenase	1	1
pfam12399	Branched-chain amino acid ATP-binding cassette transporter	1	7
pfam12404	Peptidase	1	0
pfam12412	Protein of unknown function (DUF3667)	0	1
pfam12418	Acyl-CoA dehydrogenase N terminal	2	3
pfam12434	Malate dehydrogenase enzyme	0	1
pfam12447	Protein of unknown function (DUF3683)	0	1
pfam12450	von Willebrand factor	1	0
pfam12464	Maltose acetyltransferase	2	0
pfam12513	Mitochondrial degradasome RNA helicase subunit C terminal	0	1
pfam12514	Protein of unknown function (DUF3718)	1	0
pfam12531	DNA-K related protein	0	1
pfam12532	Protein of unknown function (DUF3732)	0	1

pfam12536	Patatin phospholipase	0	2
pfam12557	Cob(I)alamin adenosyltransferase N terminal	1	1
pfam12564	Type III restriction/modification enzyme methylation subunit	1	0
pfam12571	Phage tail-collar fibre protein	1	0
pfam12614	Ribosome recycling factor	1	0
pfam12625	Arabinose-binding domain of AraC transcription regulator, N-term	0	5
pfam12626	Polymerase A arginine-rich C-terminus	1	1
pfam12627	Probable RNA and SrmB- binding site of polymerase A	2	2
pfam12631	MnmE helical domain	1	1
pfam12633	Adenylate cyclase NT domain	1	0
pfam12643	MazG-like family	0	1
pfam12679	ABC-2 family transporter protein	1	0
pfam12680	SnoaL-like domain	5	4
pfam12681	Glyoxalase-like domain	0	1
pfam12682	Flavodoxin	0	1
pfam12684	PDDEXK-like domain of unknown function (DUF3799)	1	0
pfam12690	Intracellular proteinase inhibitor	1	0
pfam12693	GspL periplasmic domain	1	1
pfam12696	TraM recognition site of TraD and TraG	2	0
pfam12697	Alpha/beta hydrolase family	2	18
pfam12698	ABC-2 family transporter protein	4	2
pfam12704	MacB-like periplasmic core domain	5	12
pfam12705	PD-(D/E)XK nuclease superfamily	0	1
pfam12706	Beta-lactamase superfamily domain	0	3
pfam12708	Pectate lyase superfamily protein	0	1
pfam12710	haloacid dehalogenase-like hydrolase	2	2
pfam12724	Flavodoxin domain	3	0
pfam12727	PBP superfamily domain	1	0
pfam12728	Helix-turn-helix domain	4	0
pfam12729	Four helix bundle sensory module for signal transduction	6	29
pfam12730	ABC-2 family transporter protein	1	1
pfam12740	Chlorophyllase enzyme	0	1
pfam12765	HEAT repeat associated with sister chromatid cohesion	0	1
pfam12769	4TM region of pyridine nucleotide transhydrogenase, mitoch	1	1

pfam12770	CHAT domain	0	1
pfam12773	Double zinc ribbon	0	1
pfam12779	YXWGXW repeat (2 copies)	0	3
pfam12787	EcsC protein family	0	1
pfam12792	CSS motif domain associated with EAL	1	0
pfam12794	Mechanosensitive ion channel inner membrane domain 1	1	0
pfam12796	Ankyrin repeats (3 copies)	1	1
pfam12800	4Fe-4S binding domain	0	1
pfam12801	4Fe-4S binding domain	3	1
pfam12802	MarR family	1	7
pfam12804	MobA-like NTP transferase domain	4	3
pfam12805	FUSC-like inner membrane protein yccS	0	1
pfam12806	Acetyl-CoA dehydrogenase C-terminal like	2	3
pfam12821	Threonine/Serine exporter, ThrE	2	0
pfam12826	Helix-hairpin-helix motif	0	1
pfam12832	MFS_1 like family	1	1
pfam12833	Helix-turn-helix domain	16	52
pfam12836	Helix-hairpin-helix motif	2	2
pfam12837	4Fe-4S binding domain	1	0
pfam12838	4Fe-4S dicluster domain	3	3
pfam12840	Helix-turn-helix domain	1	6
pfam12843	Putative quorum-sensing-regulated virulence factor	1	1
pfam12844	Helix-turn-helix domain	1	1
pfam12847	Methyltransferase domain	1	0
pfam12848	**ABC transporter**	**6**	**4**
pfam12849	PBP superfamily domain	2	1
pfam12850	Calcineurin-like phosphoesterase superfamily domain	0	1
pfam12852	Cupin	0	6
pfam12857	TOBE-like domain	1	1
pfam12860	PAS fold	2	0
pfam12867	DinB superfamily	1	1
pfam12872	OST-HTH/LOTUS domain	0	1
pfam12893	Putative lumazine-binding	2	0
pfam12897	Alanine-glyoxylate amino-transferase	0	1

pfam12900	Pyridoxamine 5'-phosphate oxidase	0	1
pfam12911	N-terminal TM domain of oligopeptide transport permease C	1	1
pfam12915	Protein of unknown function (DUF3833)	1	1
pfam12945	Flagellar protein YcgR	0	1
pfam12951	Passenger-associated-transport-repeat	0	1
pfam12969	Domain of Unknown Function with PDB structure (DUF3857)	0	2
pfam12973	ChrR Cupin-like domain	2	1
pfam12974	ABC transporter, phosphonate, periplasmic substrate-binding protein	2	3
pfam13000	Acetyl-coenzyme A transporter 1	1	0
pfam13006	Insertion element 4 transposase N-terminal	5	0
pfam13007	Transposase C of IS166 homeodomain	5	0
pfam13021	Domain of unknown function (DUF3885)	0	1
pfam13023	HD domain	1	1
pfam13036	Peptidoglycan-synthase activator LpoB	1	0
pfam13085	2Fe-2S iron-sulfur cluster binding domain	3	1
pfam13086	AAA domain	1	1
pfam13087	AAA domain	1	1
pfam13088	BNR repeat-like domain	0	1
pfam13089	Polyphosphate kinase N-terminal domain	1	1
pfam13090	Polyphosphate kinase C-terminal domain	1	1
pfam13091	PLD-like domain	2	3
pfam13098	Thioredoxin-like domain	1	1
pfam13103	TonB C terminal	0	2
pfam13116	Protein of unknown function	1	1
pfam13144	Chaperone for flagella basal body P-ring formation	2	2
pfam13145	PPIC-type PPIASE domain	0	2
pfam13155	Toprim-like	1	1
pfam13163	Protein of unknown function (DUF3999)	0	1
pfam13166	AAA domain	2	0
pfam13167	GTP-binding GTPase N-terminal	1	1
pfam13174	Tetratricopeptide repeat	1	1
pfam13175	AAA ATPase domain	1	2
pfam13176	Tetratricopeptide repeat	0	1
pfam13177	DNA polymerase III, delta subunit	2	2

pfam13180	PDZ domain	6	8
pfam13181	Tetratricopeptide repeat	4	3
pfam13183	4Fe-4S dicluster domain	5	1
pfam13184	NusA-like KH domain	1	1
pfam13185	GAF domain	7	14
pfam13186	Iron-sulfur cluster-binding domain	0	1
pfam13187	4Fe-4S dicluster domain	1	0
pfam13188	PAS domain	3	7
pfam13191	AAA ATPase domain	0	3
pfam13192	Thioredoxin domain	2	1
pfam13193	AMP-binding enzyme C-terminal domain	6	9
pfam13195	Protein of unknown function (DUF4011)	0	1
pfam13202	EF hand	0	1
pfam13211	Protein of unknown function (DUF4019)	0	1
pfam13229	Right handed beta helix region	0	4
pfam13230	Glutamine amidotransferases class-II	1	2
pfam13231	Dolichyl-phosphate-mannose-protein mannosyltransferase	0	5
pfam13237	4Fe-4S dicluster domain	2	0
pfam13240	zinc-ribbon domain	0	2
pfam13241	Putative NAD(P)-binding	1	0
pfam13242	HAD-hyrolase-like	2	0
pfam13245	AAA domain	1	2
pfam13247	4Fe-4S dicluster domain	6	1
pfam13274	Protein of unknown function (DUF4065)	0	2
pfam13275	S4 domain	1	1
pfam13276	HTH-like domain	25	1
pfam13279	Thioesterase-like superfamily	4	4
pfam13280	WYL domain	1	2
pfam13286	Phosphohydrolase-associated domain	1	1
pfam13288	DXP reductoisomerase C-terminal domain	1	1
pfam13291	ACT domain	2	2
pfam13292	1-deoxy-D-xylulose-5-phosphate synthase	2	1
pfam13296	Putative type VI secretion system Rhs element Vgr	0	5
pfam13298	DNA polymerase Ligase (LigD)	0	1

pfam13302	Acetyltransferase (GNAT) domain	8	10
pfam13304	AAA domain, putative AbiEii toxin, Type IV TA system	1	2
pfam13305	WHG domain	1	0
pfam13307	Helicase C-terminal domain	2	2
pfam13309	HTH domain	1	1
pfam13311	Protein of unknown function (DUF4080)	0	1
pfam13327	Type III secretion system subunit	1	0
pfam13328	HD domain	2	3
pfam13333	Integrase core domain	1	0
pfam13335	Magnesium chelatase, subunit ChlI C-terminal	1	1
pfam13336	Acetyl-CoA hydrolase/transferase C-terminal domain	1	1
pfam13342	C-terminal repeat of topoisomerase	0	1
pfam13343	Bacterial extracellular solute-binding protein	1	0
pfam13344	Haloacid dehalogenase-like hydrolase	1	0
pfam13347	MFS/sugar transport protein	1	3
pfam13349	Putative adhesin	1	0
pfam13353	4Fe-4S single cluster domain	5	2
pfam13354	Beta-lactamase enzyme family	0	1
pfam13356	Domain of unknown function (DUF4102)	0	1
pfam13358	DDE superfamily endonuclease	1	0
pfam13360	PQQ-like domain	1	1
pfam13361	UvrD-like helicase C-terminal domain	5	5
pfam13362	Toprim domain	1	0
pfam13365	Trypsin-like peptidase domain	2	4
pfam13369	Transglutaminase-like superfamily	1	0
pfam13371	Tetratricopeptide repeat	1	0
pfam13372	Alginate export	0	1
pfam13374	Tetratricopeptide repeat	0	2
pfam13375	RnfC Barrel sandwich hybrid domain	1	0
pfam13376	Bacteriocin-protection, YdeI or OmpD-Associated	0	2
pfam13377	Periplasmic binding protein-like domain	4	17
pfam13378	Enolase C-terminal domain-like	1	4
pfam13379	NMT1-like family	0	4
pfam13380	CoA binding domain	1	1

pfam13384	Homeodomain-like domain	1	0
pfam13385	Concanavalin A-like lectin/glucanases superfamily	2	0
pfam13386	Cytochrome C biogenesis protein transmembrane region	1	1
pfam13387	Domain of unknown function (DUF4105)	0	1
pfam13392	HNH endonuclease	0	1
pfam13393	Histidyl-tRNA synthetase	1	2
pfam13394	4Fe-4S single cluster domain	2	1
pfam13395	HNH endonuclease	1	0
pfam13398	Peptidase M50B-like	1	0
pfam13399	LytR cell envelope-related transcriptional attenuator	0	2
pfam13400	Putative Flp pilus-assembly TadE/G-like	0	3
pfam13401	AAA domain	5	3
pfam13402	Peptidase M60, enhancin and enhancin-like	1	0
pfam13404	AsnC-type helix-turn-helix domain	2	3
pfam13406	Transglycosylase SLT domain	2	1
pfam13407	Periplasmic binding protein domain	2	5
pfam13408	Recombinase zinc beta ribbon domain	0	2
pfam13410	Glutathione S-transferase, C-terminal domain	1	2
pfam13411	MerR HTH family regulatory protein	4	8
pfam13412	Winged helix-turn-helix DNA-binding	3	5
pfam13413	Helix-turn-helix domain	1	1
pfam13414	TPR repeat	0	3
pfam13416	Bacterial extracellular solute-binding protein	1	5
pfam13417	Glutathione S-transferase, N-terminal domain	7	10
pfam13419	Haloacid dehalogenase-like hydrolase	8	11
pfam13420	Acetyltransferase (GNAT) domain	0	2
pfam13421	SPFH domain-Band 7 family	0	1
pfam13424	Tetratricopeptide repeat	3	4
pfam13426	PAS domain	16	20
pfam13432	Tetratricopeptide repeat	3	11
pfam13433	Periplasmic binding protein domain	0	1
pfam13434	L-lysine 6-monooxygenase (NADPH-requiring)	1	0
pfam13437	HlyD family secretion protein	7	8
pfam13438	Domain of unknown function (DUF4113)	2	0

pfam13439	Glycosyltransferase Family 4	1	3
pfam13440	Polysaccharide biosynthesis protein	0	1
pfam13441	YMGG-like Gly-zipper	1	0
pfam13442	Cytochrome C oxidase, cbb3-type, subunit III	5	11
pfam13443	Cro/C1-type HTH DNA-binding domain	2	2
pfam13444	Acetyltransferase (GNAT) domain	1	1
pfam13449	Esterase-like activity of phytase	1	2
pfam13450	NAD(P)-binding Rossmann-like domain	3	6
pfam13454	FAD-NAD(P)-binding	0	1
pfam13456	Reverse transcriptase-like	0	1
pfam13458	Periplasmic binding protein	0	7
pfam13460	NAD(P)H-binding	3	4
pfam13462	Thioredoxin	2	2
pfam13463	Winged helix DNA-binding domain	1	1
pfam13464	Domain of unknown function (DUF4115)	1	1
pfam13466	STAS domain	1	3
pfam13468	Glyoxalase-like domain	0	1
pfam13470	PIN domain	1	1
pfam13472	GDSL-like Lipase/Acylhydrolase family	1	9
pfam13474	SnoaL-like domain	2	2
pfam13476	AAA domain	0	4
pfam13478	XdhC Rossmann domain	1	2
pfam13481	AAA domain	1	1
pfam13483	Beta-lactamase superfamily domain	0	1
pfam13484	4Fe-4S double cluster binding domain	1	1
pfam13487	HD domain	6	5
pfam13488	Glycine zipper	1	2
pfam13489	Methyltransferase domain	4	3
pfam13490	Putative zinc-finger	0	1
pfam13491	4TM region of DNA translocase FtsK/SpoIIIE	1	1
pfam13492	GAF domain	1	3
pfam13493	Domain of unknown function (DUF4118)	1	1
pfam13495	Phage integrase, N-terminal SAM-like domain	1	0
pfam13500	AAA domain	2	1

pfam13501	Sulfur oxidation protein SoxY	0	1
pfam13502	AsmA-like C-terminal region	1	1
pfam13503	Domain of unknown function (DUF4123)	0	2
pfam13505	Outer membrane protein beta-barrel domain	6	5
pfam13507	CobB/CobQ-like glutamine amidotransferase domain	1	1
pfam13508	Acetyltransferase (GNAT) domain	5	0
pfam13509	S1 domain	1	0
pfam13510	2Fe-2S iron-sulfur cluster binding domain	1	2
pfam13511	Domain of unknown function (DUF4124)	2	4
pfam13515	Fusaric acid resistance protein-like	1	2
pfam13517	Repeat domain in Vibrio, Colwellia, Bradyrhizobium and Shewanella	0	1
pfam13519	von Willebrand factor type A domain	1	1
pfam13520	Amino acid permease	4	5
pfam13521	AAA domain	0	2
pfam13522	Glutamine amidotransferase domain	1	2
pfam13523	Acetyltransferase (GNAT) domain	1	0
pfam13525	Outer membrane lipoprotein	1	2
pfam13528	Glycosyl transferase family 1	1	0
pfam13531	Bacterial extracellular solute-binding protein	4	4
pfam13532	2OG-Fe(II) oxygenase superfamily	1	1
pfam13533	Biotin-lipoyl like	6	9
pfam13534	4Fe-4S dicluster domain	1	1
pfam13537	Glutamine amidotransferase domain	2	0
pfam13538	UvrD-like helicase C-terminal domain	1	2
pfam13539	D-alanyl-D-alanine carboxypeptidase	0	2
pfam13541	Subunit ChlI of Mg-chelatase	2	1
pfam13545	Crp-like helix-turn-helix domain	2	11
pfam13549	ATP-grasp domain	1	2
pfam13555	P-loop containing region of AAA domain	0	2
pfam13556	PucR C-terminal helix-turn-helix domain	0	1
pfam13557	Putative MetA-pathway of phenol degradation	0	2
pfam13558	Putative exonuclease SbcCD, C subunit	1	2
pfam13559	Domain of unknown function (DUF4129)	1	1
pfam13560	Helix-turn-helix domain	2	2

pfam13561	Enoyl-(Acyl carrier protein) reductase	11	34
pfam13563	2'-5' RNA ligase superfamily	0	1
pfam13564	DoxX-like family	1	0
pfam13566	Domain of unknown function (DUF4130	0	1
pfam13567	Domain of unknown function (DUF4131)	1	1
pfam13569	Domain of unknown function (DUF4132)	0	1
pfam13577	SnoaL-like domain	0	1
pfam13580	SIS domain	1	1
pfam13581	Histidine kinase-like ATPase domain	1	2
pfam13582	Metallo-peptidase family M12B Reprolysin-like	0	1
pfam13584	Oxygen tolerance	1	0
pfam13588	Type I restriction enzyme R protein N terminus (HSDR_N)	2	0
pfam13589	Histidine kinase-, DNA gyrase B-, and HSP90-like ATPase	2	1
pfam13590	Domain of unknown function (DUF4136)	3	1
pfam13591	MerR HTH family regulatory protein	0	1
pfam13593	SBF-like CPA transporter family (DUF4137)	0	1
pfam13596	PAS domain	0	1
pfam13597	Anaerobic ribonucleoside-triphosphate reductase	1	0
pfam13599	Pentapeptide repeats (9 copies)	1	2
pfam13602	Zinc-binding dehydrogenase	1	3
pfam13603	Leucyl-tRNA synthetase, Domain 2	1	1
pfam13604	AAA domain	1	1
pfam13607	Succinyl-CoA ligase like flavodoxin domain	1	2
pfam13609	Gram-negative porin	6	10
pfam13612	Transposase DDE domain	1	0
pfam13614	AAA domain	5	4
pfam13616	PPIC-type PPIASE domain	3	3
pfam13617	YnbE-like lipoprotein	1	0
pfam13618	Gluconate 2-dehydrogenase subunit 3	0	4
pfam13619	KTSC domain	0	1
pfam13620	Carboxypeptidase regulatory-like domain	1	3
pfam13621	Cupin-like domain	1	5
pfam13622	Thioesterase-like superfamily	1	0
pfam13624	SurA N-terminal domain	1	2

pfam13627	Prokaryotic lipoprotein-attachment site	1	1
pfam13628	Domain of unknown function (DUF4142)	0	1
pfam13629	Pilus formation protein N terminal region	0	3
pfam13630	SdpI/YhfL protein family	0	1
pfam13632	Glycosyl transferase family group 2	1	0
pfam13636	RNA-binding PUA-like domain of methyltransferase RsmF	1	0
pfam13638	PIN domain	1	1
pfam13640	2OG-Fe(II) oxygenase superfamily	3	6
pfam13641	Glycosyltransferase like family 2	0	1
pfam13642	protein structure with unknown function	1	0
pfam13643	Domain of unknown function (DUF4145)	1	0
pfam13649	Methyltransferase domain	5	5
pfam13654	AAA domain	2	0
pfam13655	N-terminal domain of reverse transcriptase	1	0
pfam13657	HipA N-terminal domain	1	0
pfam13660	Domain of unknown function (DUF4147)	0	1
pfam13662	Toprim domain	1	1
pfam13663	Domain of unknown function (DUF4148)	0	1
pfam13664	Domain of unknown function (DUF4149)	0	1
pfam13667	ThiC-associated domain	0	1
pfam13669	Glyoxalase/Bleomycin resistance protein/Dioxygenase superfamily	1	1
pfam13670	Peptidase propeptide and YPEB domain	1	0
pfam13671	AAA domain	2	0
pfam13672	Protein phosphatase 2C	0	3
pfam13673	Acetyltransferase (GNAT) domain	7	5
pfam13675	Type IV pili methyl-accepting chemotaxis transducer N-term	1	1
pfam13676	TIR domain	0	1
pfam13677	Membrane MotB of proton-channel complex MotA/MotB	2	3
pfam13679	Methyltransferase domain	2	1
pfam13681	Type IV pilus assembly protein PilX C-term	0	1
pfam13682	Chemoreceptor zinc-binding domain	1	0
pfam13683	Integrase core domain	2	0
pfam13687	Domain of unknown function (DUF4153)	0	1
pfam13690	Chemotaxis phosphatase CheX	1	0

pfam13692	Glycosyl transferases group 1	0	2
pfam13700	Domain of unknown function (DUF4158)	4	0
pfam13708	Domain of unknown function (DUF4942)	1	0
pfam13709	Domain of unknown function (DUF4159)	0	1
pfam13710	ACT domain	1	1
pfam13714	Phosphoenolpyruvate phosphomutase	3	2
pfam13720	Udp N-acetylglucosamine O-acyltransferase; Domain 2	1	1
pfam13721	SecD export protein N-terminal TM region	2	1
pfam13722	5TM C-terminal transporter carbon starvation CstA	2	1
pfam13723	Beta-ketoacyl synthase, N-terminal domain	1	0
pfam13726	Na+-H+ antiporter family	1	0
pfam13727	CoA-binding domain	1	1
pfam13728	F plasmid transfer operon protein	1	0
pfam13730	Helix-turn-helix domain	1	0
pfam13737	Transposase DDE domain	11	0
pfam13740	ACT domain	2	0
pfam13742	OB-fold nucleic acid binding domain	1	1
pfam13744	Helix-turn-helix domain	1	0
pfam13746	4Fe-4S dicluster domain	2	1
pfam13750	Bacterial Ig-like domain (group 3)	2	0
pfam13752	Domain of unknown function (DUF4165)	2	0
pfam13761	Domain of unknown function (DUF4166)	0	1
pfam13768	von Willebrand factor type A domain	1	0
pfam13776	Domain of unknown function (DUF4172)	1	0
pfam13779	Domain of unknown function (DUF4175)	0	1
pfam13785	Domain of unknown function (DUF4178)	0	1
pfam13793	N-terminal domain of ribose phosphate pyrophosphokinase	1	1
pfam13795	HupE / UreJ protein	0	2
pfam13801	Heavy-metal resistance	0	1
pfam13802	Galactose mutarotase-like	0	2
pfam13806	Rieske-like [2Fe-2S] domain	0	1
pfam13807	G-rich domain on putative tyrosine kinase	1	1
pfam13814	Replication-relaxation	0	1
pfam13817	IS66 C-terminal element	5	0

pfam13835	Domain of unknown function (DUF4194)	0	1
pfam13840	ACT domain	2	2
pfam13844	Glycosyl transferase family 41	0	1
pfam13847	Methyltransferase domain	3	0
pfam13852	Protein of unknown function (DUF4197)	0	1
pfam13855	Leucine rich repeat	1	1
pfam13860	FlgD Ig-like domain	2	2
pfam13861	FlgD Tudor-like domain	2	1
pfam13899	Thioredoxin-like	4	3
pfam13924	Lipocalin-like domain	0	1
pfam13932	GidA associated domain	1	1
pfam13936	Helix-turn-helix domain	29	0
pfam13937	Domain of unknown function (DUF4212)	1	1
pfam13946	Domain of unknown function (DUF4214)	0	3
pfam13953	PapC C-terminal domain	0	1
pfam13972	Bacterial transcriptional repressor	1	1
pfam13977	Bacterial transcriptional repressor	1	1
pfam13986	Domain of unknown function (DUF4224)	0	1
pfam14018	Domain of unknown function (DUF4234)	1	0
pfam14024	Protein of unknown function (DUF4240)	1	0
pfam14031	Putative serine dehydratase domain	0	1
pfam14052	Capsule assembly protein Wzi	1	0
pfam14070	Putative motility protein	0	1
pfam14076	Domain of unknown function (DUF4258)	1	0
pfam14078	Domain of unknown function (DUF4259)	0	1
pfam14086	Domain of unknown function (DUF4266)	1	1
pfam14094	Domain of unknown function (DUF4272)	0	1
pfam14113	Type VI secretion system (T6SS), amidase effector protein 4	0	1
pfam14117	Domain of unknown function (DUF4287)	1	0
pfam14163	Super-infection exclusion protein B	2	0
pfam14224	Domain of unknown function (DUF4331)	0	1
pfam14226	non-haem dioxygenase in morphine synthesis N-terminal	2	0
pfam14234	Domain of unknown function (DUF4336)	1	0
pfam14235	Domain of unknown function (DUF4337)	0	2

pfam14246	AefR-like transcriptional repressor, C-terminal region	0	1
pfam14252	Domain of unknown function (DUF4347)	0	1
pfam14255	Cysteine-rich CPXCG	1	0
pfam14257	Domain of unknown function (DUF4349)	0	1
pfam14261	Domain of unknown function (DUF4351)	0	4
pfam14280	Domain of unknown function (DUF4365)	1	0
pfam14282	FlxA-like protein	1	1
pfam14284	PcfJ-like protein	1	0
pfam14310	Fibronectin type III-like domain	0	4
pfam14316	Domain of unknown function (DUF4381)	1	0
pfam14317	YcxB-like protein	1	0
pfam14331	ImcF-related N-terminal domain	0	2
pfam14334	Domain of unknown function (DUF4390)	0	1
pfam14341	PilX N-terminal	1	0
pfam14345	GDYXXLXY protein	1	1
pfam14346	Domain of unknown function (DUF4398)	0	2
pfam14347	Domain of unknown function (DUF4399)	0	1
pfam14348	Domain of unknown function (DUF4400)	2	0
pfam14351	Domain of unknown function (DUF4401)	1	1
pfam14355	Abortive infection C-terminus	0	1
pfam14357	Domain of unknown function (DUF4404)	0	1
pfam14361	RsbT co-antagonist protein rsbRD N-terminal domain	0	1
pfam14375	Cysteine-rich CWC	1	1
pfam14384	BrnA antitoxin of type II toxin-antitoxin system	0	1
pfam14397	Sugar-transfer associated ATP-grasp	1	0
pfam14400	Inactive transglutaminase fused to 7 transmembrane helices	1	0
pfam14402	7 transmembrane helices usually fused to an inactive transglutaminase	1	0
pfam14403	Circularly permuted ATP-grasp type 2	0	1
pfam14415	Domain of unknown function (DUF4424)	0	1
pfam14437	MafB19-like deaminase	1	1
pfam14450	Cell division protein FtsA	1	1
pfam14467	Domain of unknown function (DUF4426)	1	0
pfam14489	QueF-like protein	1	1
pfam14492	Elongation Factor G, domain II	2	2

pfam14497	Glutathione S-transferase, C-terminal domain	2	2
pfam14498	Glycosyl hydrolase family 65, N-terminal domain	0	1
pfam14501	GHKL domain	0	1
pfam14508	Glycosyl-hydrolase 97 N-terminal	1	3
pfam14509	Glycosyl-hydrolase 97 C-terminal, oligomerisation	1	3
pfam14518	Iron-containing redox enzyme	0	1
pfam14520	Helix-hairpin-helix domain	4	2
pfam14524	Wzt C-terminal domain	0	1
pfam14525	AraC-binding-like domain	0	1
pfam14526	Integron-associated effector binding protein	1	0
pfam14534	Domain of unknown function (DUF4440)	2	5
pfam14535	AMP-binding enzyme C-terminal domain	0	1
pfam14537	Cytochrome c3	3	0
pfam14539	Domain of unknown function (DUF4442)	1	0
pfam14549	DNA-binding transcriptional regulator Cro	1	0
pfam14552	Tautomerase enzyme	0	1
pfam14559	Tetratricopeptide repeat	2	6
pfam14561	Tetratricopeptide repeat	1	0
pfam14567	SMI1-KNR4 cell-wall	1	0
pfam14568	SMI1-KNR4 cell-wall	1	0
pfam14572	Phosphoribosyl synthetase-associated domain	1	1
pfam14579	Helix-hairpin-helix motif	1	3
pfam14581	SseB protein C-terminal domain	1	0
pfam14588	YjgF/chorismate_mutase-like, putative endoribonuclease	0	1
pfam14590	Domain of unknown function (DUF4447)	1	0
pfam14595	Thioredoxin	1	0
pfam14600	Cellulose-binding domain	1	0
pfam14602	Hexapeptide repeat of succinyl-transferase	4	4
pfam14606	GDSL-like Lipase/Acylhydrolase family	0	1
pfam14622	Ribonuclease-III-like	1	1
pfam14659	Phage integrase, N-terminal SAM-like domain	4	1
pfam14681	Uracil phosphoribosyltransferase	1	1
pfam14684	Tricorn protease C1 domain	1	1
pfam14685	Tricorn protease PDZ domain	1	1

pfam14689	Sensor_kinase_SpoOB-type, alpha-helical domain	1	0
pfam14691	Dihydroprymidine dehydrogenase domain II, 4Fe-4S cluster	2	2
pfam14693	Ribosomal protein TL5, C-terminal domain	0	1
pfam14696	Hydroxyphenylpyruvate dioxygenase, HPPD, N-terminal	1	1
pfam14697	4Fe-4S dicluster domain	1	2
pfam14698	Argininosuccinate lyase C-terminal	1	1
pfam14714	KH-domain-like of EngA bacterial GTPase enzymes, C-terminal	1	1
pfam14715	N-terminal domain of cytochrome oxidase-cbb3, FixP	1	1
pfam14718	Soluble lytic murein transglycosylase L domain	1	0
pfam14720	NiFe/NiFeSe hydrogenase small subunit C-terminal	1	0
pfam14743	DNA ligase OB-like domain	1	0
pfam14748	Pyrroline-5-carboxylate reductase dimerisation	1	1
pfam14760	Rnk N-terminus	1	0
pfam14765	Polyketide synthase dehydratase	1	0
pfam14793	Domain of unknown function (DUF4478)	1	1
pfam14803	Nudix N-terminal	0	1
pfam14805	Tetrahydrodipicolinate N-succinyltransferase N-terminal	1	1
pfam14814	Bifunctional transglycosylase second domain	1	0
pfam14815	NUDIX domain	1	1
pfam14819	Nitrile reductase, 7-cyano-7-deazaguanine-reductase N-term	1	1
pfam14821	Threonine synthase N terminus	1	1
pfam14824	Sirohaem biosynthesis protein central	1	0
pfam14827	Double sensory domain of two-component sensor kinase	2	1
pfam14833	NAD-binding of NADP-dependent 3-hydroxyisobutyrate dehydrogenase	2	2
pfam14834	Glutathione S-transferase, C-terminal domain	0	1
pfam14840	Processivity clamp loader gamma complex DNA pol III C-term	1	1
pfam14841	FliG middle domain	2	2
pfam14842	FliG N-terminal domain	2	2
pfam14849	YidC periplasmic domain	1	1
pfam14850	DNA-binding domain of Proline dehydrogenase	1	1
pfam14863	Alkyl sulfatase dimerisation	1	0
pfam14864	Alkyl sulfatase C-terminal	1	0
pfam14870	Photosynthesis system II assembly factor YCF48	1	1
pfam14882	Phage-integrase repeat unit	1	0

pfam14891	Effector protein	0	1
pfam14897	EpsG family	1	0
pfam14905	Outer membrane protein beta-barrel family	0	9
pfam15575	Immunity protein 49	3	0
pfam15644	Papain fold toxin 1, glutamine deamidase	1	0
pfam15780	Abnormal spindle-like microcephaly-assoc'd, ASPM-SPD-2-Hydin	0	1
pfam15781	ParE-like toxin of type II bacterial toxin-antitoxin system	0	1
pfam15892	BNR repeat-containing family member	0	1
pfam15919	HicB_like antitoxin of bacterial toxin-antitoxin system	0	1
pfam15956	Domain of unknown function (DUF4760)	0	1
pfam15975	Flotillin	1	0
pfam15978	Tn7-like transposition protein D	2	0
pfam15979	Glycosyl hydrolase family 115	0	1
pfam16024	Domain of unknown function (DUF4785)	1	0
pfam16036	Chalcone isomerase-like	2	2
pfam16068	Domain of unknown function (DUF4810)	0	1
pfam16074	Type IV Pilus-assembly protein W	1	2
pfam16078	2-oxoglutarate dehydrogenase N-terminus	1	1
pfam16080	Bacteriophage holin family HP1	2	0
pfam16108	Domain of unknown function (DUF4826)	1	0
pfam16113	Enoyl-CoA hydratase/isomerase	1	1
pfam16123	Hydroxyacylglutathione hydrolase C-terminus	1	1
pfam16124	RecQ zinc-binding	2	2
pfam16133	Domain of unknown function (DUF4844)	0	2
pfam16137	Domain of unknown function (DUF4845)	0	1
pfam16153	Domain of unknown function (DUF4861)	0	2
pfam16157	Domain of unknown function (DUF4865)	0	1
pfam16177	Acetyl-coenzyme A synthetase N-terminus	2	2
pfam16184	Cadherin-like	1	0
pfam16187	Middle or third domain of peptidase_M16	1	0
pfam16188	C-terminal region of peptidase_M24	1	1
pfam16189	Creatinase/Prolidase N-terminal domain	1	1
pfam16193	AAA C-terminal domain	1	1
pfam16197	Ketoacyl-synthetase C-terminal extension	1	0

pfam16198	tRNA pseudouridylate synthase B C-terminal domain	1	1
pfam16199	Radical_SAM C-terminal domain	1	0
pfam16200	C-terminal region of band_7	2	1
pfam16217	Peptidase M64 N-terminus	0	1
pfam16220	Domain of unknown function (DUF4880)	1	3
pfam16234	Domain of unknown function (DUF4892)	1	1
pfam16242	Pyridoxamine 5'-phosphate oxidase like	0	2
pfam16263	Domain of unknown function (DUF4917)	1	0
pfam16290	Domain of unknown function (DUF4936)	0	1
pfam16313	Met-zincin	1	2
pfam16320	Ribosomal protein L7/L12 dimerisation domain	1	1
pfam16325	Peptidase family U32 C-terminal domain	1	1
pfam16326	ABC transporter C-terminal domain	1	1
pfam16327	Cytochrome c-type biogenesis protein CcmF C-terminal	1	0
pfam16331	TolA binding protein trimerisation	1	1
pfam16332	Domain of unknown function (DUF4962)	0	1
pfam16338	Domain of unknown function (DUF4968)	0	1
pfam16355	Domain of unknown function (DUF4982)	0	2
pfam16357	Putative PepSY_TM-like	1	1
pfam16358	RcsF lipoprotein	1	0
pfam16360	GTP-binding GTPase Middle Region	1	1
pfam16363	GDP-mannose 4,6 dehydratase	3	5
pfam16369	C-terminal of Glycosyl hydrolases family 43	0	1
pfam16375	Domain of unknown function	0	1
pfam16401	Domain of unknown function (DUF5009)	1	0
pfam16448	LapD/MoxY periplasmic domain	1	0
pfam16452	Bacteriophage CI repressor C-terminal domain	1	0
pfam16491	CAAX prenyl protease N-terminal, five membrane helices	0	1
pfam16499	Alpha galactosidase A	0	1
pfam16524	Periplasmic domain of Sensor histidine kinase RisS	0	1
pfam16537	Type II secretion system protein B	1	1
pfam16538	Flagellar assembly protein T, C-terminal domain	1	0
pfam16539	Flagellar assembly protein T, middle domain	1	0
pfam16548	Flagellar assembly protein T, N-terminal domain	1	0

pfam16576	Barrel-sandwich domain of CusB or HlyD membrane-fusion	15	17
pfam16582	Middle domain of thiamine pyrophosphate	1	0
pfam16591	Helical bimodular sensor domain	1	0
pfam16655	PhoD-like phosphatase, N-terminal domain	1	3
pfam16657	Maltogenic Amylase, C-terminal domain	0	1
pfam16658	Class II release factor RF3, C-terminal domain	1	1
pfam16661	Metallo-beta-lactamase superfamily domain	1	0
pfam16691	Domain of unknown function (DUF5062)	1	0
pfam16693	Inner membrane component of T3SS, periplasmic domain	1	0
pfam16697	Inner membrane component of T3SS, cytoplasmic domain	0	1
pfam16732	Type IV minor pilin ComP, DNA uptake sequence receptor	1	2
pfam16859	Bacterial transcriptional repressor C-terminal	0	2
pfam16868	NMT1-like family	1	1
pfam16870	2-oxoglutarate dehydrogenase C-terminal	1	1
pfam16872	Putative phage abortive infection protein	1	0
pfam16881	N-terminal domain of lipoyl synthase of Radical_SAM family	0	1
pfam16884	N-terminal domain of oxidoreductase	1	2
pfam16901	C-terminal domain of alpha-glycerophosphate oxidase	0	2
pfam16916	Dimerisation domain of Zinc Transporter	1	0
pfam16921	Tex protein YqgF-like domain	1	1
pfam16925	Bacterial transcriptional repressor C-terminal	2	2
pfam16927	N-terminal 7TM region of histidine kinase	0	1
pfam16956	Putative general bacterial porin	1	0
pfam16957	Malonate decarboxylase, alpha subunit, transporter	0	1
pfam16976	Flp pilus assembly protein RcpC/CpaB	0	3
pfam16983	Molybdate transporter of MFS superfamily	1	0
pfam16986	Heavy-metal resistance protein CzcE	0	3
pfam17001	Type III secretion basal body protein I, YscI, HrpB, PscI	1	0
pfam17092	Penicillin-binding protein OB-like domain	1	2
pfam17113	Regulatory signalling modulator protein AmpE	1	0
pfam17125	N-terminal domain of 16S rRNA methyltransferase RsmF	1	1
pfam17136	Ribosomal proteins 50S L24/mitochondrial 39S L24	1	1
pfam17137	Domain of unknown function (DUF5110)	0	3
pfam17148	Domain of unknown function (DUF5117)	1	1

pfam17149		Periplasmic sensor domain found in signal transduction proteins	1	0
pfam17152		Periplasmic sensor domain	0	1
pfam17158		Membrane-associated sensor, integral membrane domain	1	0
pfam17162		Domain of unknown function (DUF5118)	1	0
pfam17167		Glycosyl hydrolase 36 superfamily, catalytic domain	0	3
pfam17186		Lipocalin-like domain	1	1
pfam17188		MucB/RseB C-terminal domain	1	0
pfam17189		Glycosyl hydrolase family 30 beta sandwich domain	0	1
pfam17191		RecG wedge domain	1	1
pfam17200		Single Cache domain 2	4	6
pfam17201		Cache 3/Cache 2 fusion domain	1	1
pfam17202		Single cache domain 3	1	0
pfam17203		Single cache domain 3	1	0
pfam17206		SeqA protein N-terminal domain	1	0
pfam17209		Hfq protein	1	1
pfam17210		SdrD B-like domain	0	1
pfam00072	*WP	Two-component system response regulator ArcA	1	0
pfam00775	*WP	Protocatechuate 3,4-Dioxygenase	0	1
pfam05448	*WP	Xylan esterase	0	1

*Accession number based on NCBI annotation results.
